# Signaling downstream of tumor–stroma interaction regulates mucinous colorectal adenocarcinoma apicobasal polarity

**DOI:** 10.1038/s41467-026-75127-0

**Published:** 2026-07-04

**Authors:** Nicolas Pasquier, Meri Pelkonen, Elise Carraz-Billat, Aleksi Isomursu, Raphaël Merand, Hellyeh Hamidi, Jacques R. R. Mathieu, Jouni Härkönen, Gautier Follain, Christophe Desterke, Zoé Fusilier, Junel Solis, Irina Belaya, Pasi Kankaanpää, Valeria Barresi, Mohamed-Amine Bani, Johanna Protin, Jérôme Cartry, Sabrina Bedja, Olav M. Andersen, Klaus Elenius, Florent Peglion, Fanny Jaulin, Johanna Ivaska

**Affiliations:** 1 Turku Bioscience Centre, University of Turku and Åbo Akademi University, Turku, Finland; 2 Université Paris-Saclay, Gustave Roussy, Inserm, UMR 1279 Tumor Cell Dynamics, 94805 Villejuif, France; 3 Department of Pathology, Hospital Nova, Wellbeing Services County of Central Finland, Jyväskylä, Finland; 4 Faculty of Science and Engineering, Cell Biology, Åbo Akademi University, Turku, 20520 Finland; 5 Turku Collegium for Science, Medicine and Technology (TCSMT), Turku, 20520 Finland; 6 INSERM UMR-S1310, Hôpital Paul Brousse, Université Paris-Saclay, Villejuif, France; 7 INSERM-U932, Immunity and Cancer, Institut Curie, Paris-Cité University, Paris, France; 8 INSERM-U932, Immunity and Cancer, Institut Curie, PSL University, Paris, France; 9 Turku Bioimaging, Åbo Akademi University and University of Turku, Turku, Finland; 10 Department of Diagnostics and Public Health, University of Verona, Verona, 37134 Italy; 11 Gustave Roussy, Département de Pathologie Morphologique, 94805 Villejuif, France; 12 Department of Biomedicine, Aarhus University, Aarhus, Denmark; 13 Institute of Biomedicine, and MediCity Research Laboratory, University of Turku, Turku, Finland; 14 Department of Oncology, Turku University Hospital, Turku, Finland; 15 InFLAMES Research Flagship Center, University of Turku, Turku, Finland; 16 Department of Life Technologies, University of Turku, Turku, Finland; 17 Foundation for the Finnish Cancer Institute, Helsinki, Finland; 18 Western Finnish Cancer Center, University of Turku, Turku, Finland; 19 Present Address: Cell Polarity, Migration and Cancer Unit, Institut Pasteur, CNRS UMR3691, Université Paris Cité, Paris, France

**Keywords:** Apicobasal polarity, Colorectal cancer

## Abstract

Mucinous colorectal carcinoma (MUC CRC) metastasis to multiple organs, and to the peritoneum, is associated with poor prognosis. Disseminating MUC CRCs exhibit either conventional (apical-in) or inverted (apical-out) polarity that influence patient outcomes. Therefore, it is critical to identify how MUC CRC polarity is regulated. Here, we analyze patient-derived MUC CRC xenografts with either apical-in or apical-out polarity. Single-cell analyses reveal α2β1-integrin as a key collagen-binding receptor in these models. Collagen–α2β1-integrin interaction activates Src and upregulates SorLA, an endosomal sorting receptor. SorLA supports apical-in polarity by promoting integrin recycling and HER2/HER3 expression. We observe positive correlation between HER2, HER3 and SorLA in patient samples and higher HER2 expression in apical-in-presenting tissues. Clinically relevant HER2/HER3-targeting antibodies revert tumor sphere polarity, and impede collagen remodeling and adhesion to mouse peritoneum. This SorLA—integrin—HER2/HER3 axis could represent a MUC CRC-patient stratification approach and be relevant for other carcinomas with apical-out phenotypes.

## Abstract (web summary)

Mucinous colorectal cancers form cell clusters whose apicobasal polarity influences tissue interaction and disease progression. Here, the authors show that collagen engagement activates a HER2/HER3/SorLA-β1-integrin signaling axis that promotes integrin recycling, polarity reversion, and tumor–stroma interaction.

## Introduction

Apicobasal polarization is a critical cellular process that is essential for normal tissue function. Its disruption is implicated in various pathological conditions, including cancer^1–3^. While the loss of polarity has been extensively studied, the phenomenon of inverted polarity (also referred to as apical-out polarity), where the apical and basolateral domains are reversed, is less understood.

Apical-out topology, characterized by the mislocalization of apical and basolateral markers^4–7^, is a hallmark of several aggressive cancer subtypes^8^, including micropapillary carcinomas and mucinous colorectal adenocarcinomas (MUC CRC). Micropapillary carcinomas exhibit a fully inverted apicobasal polarity^9^. This type of cancer is diagnosed upon detection of an apical-out polarity pattern. MUC CRC represents a distinct subtype of colorectal carcinoma (CRC), characterized by abundant mucinous components constituting at least 50% of the tumor volume. CRC is the third most common cancer globally and the second leading cause of cancer-related deaths^10^. MUC CRC constitutes 10–15% of CRC cases, predominantly affecting young women and the right colon. It carries a poor prognosis, particularly in metastatic disease, with a high incidence of peritoneal metastasis^11–16^. Therefore, there is an urgent need to better understand the mechanisms contributing to the acquisition of inverted polarity in cancer.

Histological analyses have revealed that MUC CRCs disseminate not as individual cells but rather as clusters of hundreds of cells called tumor spheres with inverted polarity (TSIPs)^17^. These structures, first identified in patients’ peritoneal effusions, act as tumor intermediates arising from the primary tumor and invading tissues to form metastasis in the peritoneum, facilitating cancer motility and dissemination^18^.

While these TSIPs maintain an inverted (apical-out) polarity in suspension, they display distinct responses within the tumor stroma. Once in contact with the extracellular matrix (ECM), TSIPs either revert to a normal (apical-in) polarity or remain inverted (apical-out). The mechanisms underpinning these differential polarity responses to the stromal environment are not fully understood, although the TGF-β and Src pathways have been implicated^19–21^. The apical-out polarity phenotype has recently been shown to have clinically relevant manifestations. It is supportive of a collective ameboid mode of migration, enhancing tumor invasion efficiency^18^, and significantly decreases tumor sensitivity to anti-cancer drugs^22^.

There is growing evidence from different cancer types that tumor–ECM interactions regulate cancer progression in numerous ways^23,24^. Cancer cell–ECM interactions and the mechanical properties of the ECM, orchestrated strongly by cancer-associated fibroblasts^25,26^ and cancer cells via matrix remodeling, regulate cancer cell proliferation, migration, and invasion through integrin-mediated signaling and integrin and actin-mediated force transduction to the nucleus^27^. While integrins are well-established mediators of mechanotransduction, especially in invasive cancer, their role in regulation of cancer cell polarity and how this may be regulated by endosomal receptor traffic is less well understood and has only recently been investigated in prostate cancer^28^.

Here, using patient-derived MUC CRC tumor spheres, we uncovered a previously uncharacterized mechanism of TSIP polarity orientation. Tumor sphere contact with stromal collagen induces α2β1-integrin signaling to Src, upregulates the HER2/HER3/SorLA complex activity, and increases SorLA-dependent β1-integrin recycling to the plasma membrane. This maintains tumor–stroma adhesion to support apical-in polarity. Increased ligand-induced HER3 signaling is sufficient to trigger apical-in topology in inherently apical-out tumors. Conversely, clinically used HER2/HER3-targeting antibodies inhibit apical-in phenotype. In clinical specimens, SorLA/HER2/HER3 levels are positively correlated and highest in apical-in tumors. Our study demonstrates the central role of SorLA in the regulation of integrin and HER-family receptor function in CRC, significantly contributing to our understanding of polarity regulation in this aggressive cancer type and paving the way for further investigations into the relevance of this signaling axis in MUC CRC progression. Moreover, as inverted polarity is characteristic to a number of aggressive cancers, these findings have broader implications beyond MUC CRC.

## Results

### Mucinous CRC polarity is regulated by the cells’ proximity to the ECM

**Fig. 1:**
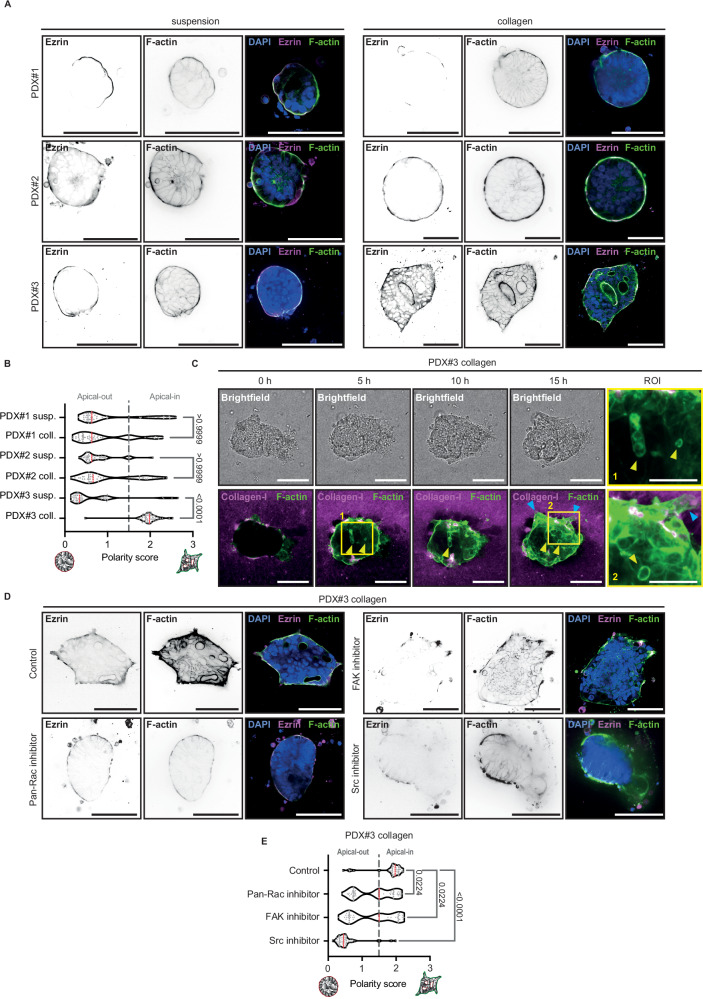
Apicobasal polarity of mucinous CRC PDXs is controlled by the focal adhesion signaling pathway. **A**, **B** Representative immunofluorescence images of PDX#1-, PDX#2-, and PDX#3-generated tumoroids, fixed in suspension or embedded in collagen and stained for Ezrin (polarity marker), F-actin, and DAPI (**A**). Comparison of polarity scores amongst the models is shown (**B**). Data are displayed as violin plots with mean indicated by a red line. Dashed line (at polarity score 1.5) indicates the mid-point between apical-in and apical-out characteristics [*n*_PDX#1_ = 51 (suspension), 49 (collagen); *n*_PDX#2_ = 43 (suspension), 51 (collagen); *n*_PDX#3_ = 51 (suspension), 52 (collagen) tumoroids from three biological replicates; Kruskal–Wallis test with Dunn’s multiple comparison test]. **C** Representative snapshots of live imaging of PDX#3 tumoroid polarity reversion following embedding in fluorescently labeled collagen (time point 0) over 15 h. Tumoroids were stained with SiR-actin for visualization. Yellow arrows indicate lumen-like structures, blue arrows indicate protrusions. **D**, **E** Representative immunofluorescence images of PDX#3 tumoroids in collagen treated with either a Pan-Rac inhibitor (EHT-1864, 5 μM), a FAK inhibitor (FAK14, 10 μM), or a Src inhibitor (saracatinib, 1 μM) and stained for Ezrin (**D**). Analysis of polarity scores after the treatments is shown (**E**). Data are displayed as violin plots with mean indicated by a red line. Dashed line (at polarity score 1.5) indicates the mid-point between apical-in and apical-out characteristics [*n* = 45 (control); 53 (EHT-1864); 36 (FAK14) and 35 (saracatinib) PDX#3 tumoroids; Kruskal–Wallis test with Dunn’s multiple comparison test]. Scale bars, 100 μm (main), 50 μm (insets). Source data provided.

To investigate the molecular mechanisms of ECM-regulated MUC CRC polarity, we used previously described patient-derived xenograft (PDX) models. We focused on three PDXs from the CReMEC bank^29^ representing mucinous histotypes of CRCs^21^ and derived either from a peritoneal metastasis (PDX#1, for LRB-009C) or from the primary tumor (PDX#2, for IGR-012P; PDX#3, for IGR-014P) (see Supplementary Table 1). We cultured the MUC CRC PDX-derived cell clusters (from here on referred to as tumoroids) either in suspension (3 days) or in suspension followed by embedding in collagen-I gels, a surrogate for the cancer stroma^30^. These two setups mimic the in vivo relevant scenarios of MUC CRC progression as peritoneal effusions or as cell clusters in tissue, respectively. In order to quantitatively score polarity phenotypes, we computed a polarity score according to three parameters: presence/absence of a lumen, presence/absence of protrusions, and Ezrin fluorescence ratio between the luminal and cortical sides (Fig. S1A). Confocal microscopy imaging of the apical marker Ezrin and quantifications revealed that two of the three PDX tumoroids (PDX#1 and PDX#2) maintain apical-out polarity in both suspension and collagen, whereas PDX#3 tumoroids in collagen revert their polarity into a multilayered structure with features of apical-in polarity, such as Ezrin-positive lumens and collagen-facing protrusions (Fig. 1A, B). While these multilayered structures are not typical one-cell layered apical-in spheroids with a unique lumen, they will be referred to as apical-in from hereon. These data are concordant with previous studies indicating that 66% of MUC CRC patients’ peritoneal effusions contained TSIPs that retained their inverted apico-basolateral polarity when transferred to collagen gels^21^. Characteristically, MUC CRCs release high levels of free mucin. Upon staining with the non-specific mucin marker WGA-lectin, we were able to visualize mucin secretions by the tumoroids. In the non-ECM responsive PDX#1 and PDX#2, mucin surrounded the spheres, indicative of inverted polarity, while in PDX#3, the mucin was concentrated within the lumen, confirming the polarity reversion—from apical-out to apical-in—in this model (Fig. S1B). However, we have not analyzed which specific mucins are secreted and if this impacts the phenotype. To investigate the collagen-induced polarity reversal in detail, we embedded the PDX#3 tumoroids into collagen gels containing fluorescently labeled collagen and performed live imaging using a filamentous actin live-cell dye (SiR-Actin). We observed transient interactions between tumoroid cells and the ECM and clear formation of lumen-like structures and protrusions as early as 5 h post-embedding (Fig. 1C, Supplementary Movie 1). Together, these data imply that only the PDX#3 tumoroids are able to respond to their ECM environment by reversing their polarity.

### Focal adhesion pathway signaling regulates collagen-induced polarity reversion

To understand the differential responses of the three tumoroid models to collagen embedding, we performed pathway analysis on available transcriptome data (GSE152299) for the three PDX models (suspension versus collagen-embedded tumoroids)^21^. These analyses revealed the Focal Adhesion Pathway as a top upregulated pathway in the matrix-responsive PDX#3 tumoroids following embedding in collagen. Upon plotting the 45 top differentially regulated genes in the KEGG Focal Adhesion Pathway, we noticed a clear distinction between PDX#3 gene expression in collagen compared to the PDX#1 and PDX#2 tumoroids (Fig. S1C). The corresponding GSEA enrichment plot^21^ as well as the Principal Component Analysis (PCA) based on the KEGG Focal Adhesion geneset confirmed a clear shift in the transcriptome signature of PDX#3 tumoroids upon collagen embedding (Fig. S1D). These data prompted us to test the functional relevance of the key focal adhesion pathway signaling components on the collagen-induced polarity reversal. We inhibited the small GTPase Rac and the tyrosine kinases focal adhesion kinase (FAK) and Src with established inhibitors^31–33^. Src, a key focal adhesion-associated tyrosine kinase, was phosphorylated and activated in PDX#3 tumoroids upon embedding in collagen (Fig. S1E, F). Strikingly, downregulation of Src activity dampened the polarity reversal by reducing protrusions, the number of lumens, and the luminal/cortical Ezrin ratio (Fig. 1D, E). Similar effects were also observed by inhibiting FAK and Rac (Fig. 1D, E). Together, these data indicate that cell–ECM interaction triggers focal adhesion-associated signaling and contributes to PDX#3 polarity reversal in collagen.

### Collagen-binding integrins are upstream of the polarity establishment signaling pathway in CRC tumoroids

**Fig. 2:**
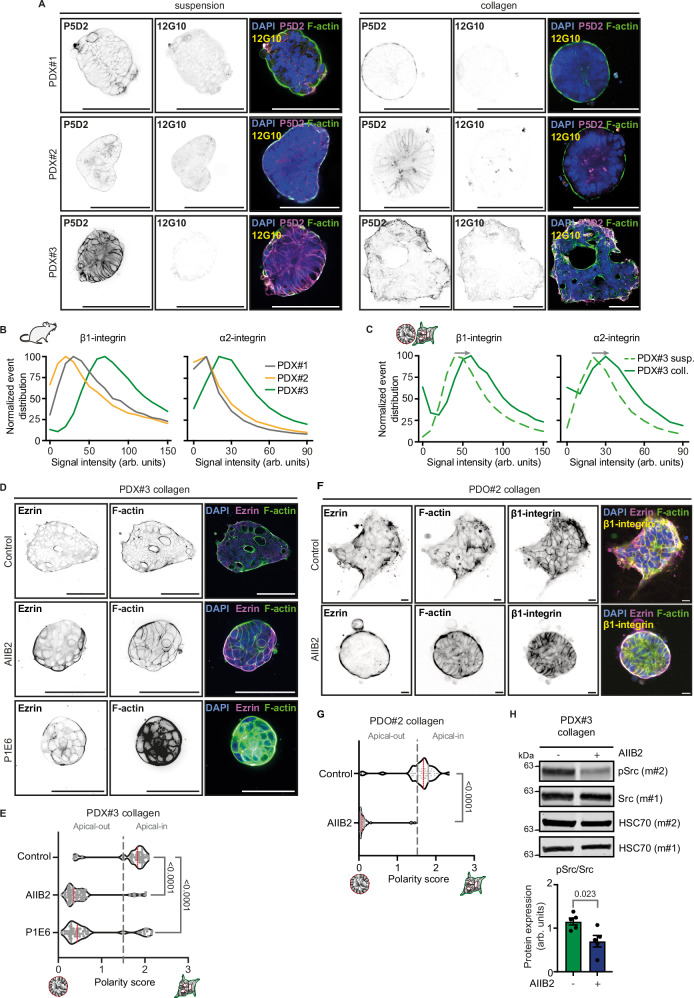
Integrin–collagen interaction controls ECM-induced polarity reversal. **A** Representative immunofluorescence images of PDX#1-, PDX#2-, and PDX#3-generated tumoroids in suspension and in collagen, stained for total β1-integrin (P5D2), active β1-integrin (12G10), F-actin, and DAPI [(*n* = 11 (PDX#1 suspension); 12 (PDX#1 collagen); 6 (PDX#2 suspension); 8 (PDX#2 collagen); 5 (PDX#3 suspension); 6 (PDX#3 collagen) tumoroids from one biological replicate)]. Scale bars 100 μm. **B** Distribution of the β1-integrin and α2-integrin surface expression signal intensity in PDX#1, PDX#2, and PDX#3 tumors out of mice (cells > 36,000). **C** Distribution of β1-integrin and α2-integrin surface expression signal intensity in PDX#3-generated tumoroids in suspension and in collagen (cells > 7000). **D** Representative immunofluorescence images of PDX#1-, PDX#2-, and PDX#3-generated tumoroids in suspension and in collagen treated with a β1-integrin blocking antibody (AIIB2, 1 μg/ml) or with an α2-integrin blocking antibody (P1E6, 10 μg/ml) and stained for Ezrin, F-actin, and nuclei (DAPI). Scale bars 100 μm. **E** Comparison of polarity scores of PDX#3 in collagen after β1-integrin and α2-integrin blocking. Data are displayed as violin plots with mean indicated by a red line. Dashed line (at polarity score 1.5) indicates the mid-point between apical-in and apical-out characteristics [(*n* = 40 (Control); 61 (AIIB2); 51 (P1E6) pooled from three biological replicates; Kruskal–Wallis test with Dunn’s multiple comparison test]. **F** Representative immunofluorescence images of PDO#2-generated spheres treated with AIIB2 and stained for the apical marker Ezrin, F-actin, and β1-integrin. Scale bar 20 µm. **G** Polarity score of PDO#2-generated spheres in collagen ± AIIB2. Data are displayed as violin plots as in (**E**). [(*n* = 45 (control); 50 (AIIB2) from two biological replicates; two-tailed Mann–Whitney test]. **H** Western blot of pSrc, Src, and HSC70 (loading control) in PDX#3-generated tumoroids in collagen ± AIIB2 (1 μg/ml), and corresponding quantification of Src phosphorylation ratio (mean ± s.e.m; *n* = 5 biological replicates; two-tailed unpaired t-test with Welch’s correction). Source data provided.

Integrins, in particular β1-integrin-containing heterodimers, are the principal ECM receptors upstream of the focal adhesion pathway and represent established polarity markers in epithelia^17,34^. We visualized β1-integrin localization and activity in the tumoroids both in suspension and in collagen. In PDX#3 tumoroids, the localization of β1-integrins (detected with the P5D2 antibody) shifted from both basal and lateral (cell–ECM and cell–cell) to basal and the amount of active β1 (detected with the 12G10 antibody) increased upon contact with the ECM (Fig. 2A). In contrast, β1-integrin intensity was lower in the PDX#1 and PDX#2 tumoroids and the receptor’s localization was not clearly altered despite the cells being embedded in collagen (Fig. 2A). We then expanded our investigation to additional CRC patient-derived organoids (PDOs; see Supplementary Table 1) and observed that PDO#1, with lower β1-integrin expression in suspension, did not respond to collagen whereas the higher β1-integrin expressing PDO#2 formed collagen-engaging protrusions, Ezrin- and F-actin-positive lumens and basal integrin, similar to PDX#3 (Fig. S2A–C).

To assess which β1-integrin heterodimers are involved, we applied high-dimensional mass cytometry analysis, focusing on cell-surface integrin receptor expression at the single-cell resolution in the PDX models under different conditions. We designed an extensive antibody panel including all the human ECM-binding integrins (see “Methods” for details). We then analyzed freshly isolated and processed samples directly from mice, digesting the resulting PDX tumoroids into a single-cell suspension. Following the mass cytometry analysis, we generated a heatmap of the median surface expression of all the integrin subunits in our panel for each PDX model. Among these integrins, the surface expression of β1- and α2-integrin subunits was markedly higher in the PDX#3 model compared to PDX#1 and PDX#2 (Fig. S2D). Interestingly, α2β1 is the main collagen I-binding integrin heterodimer^35,36^. To visualize the overall integrin cell-surface profile, we combined t-Distributed Stochastic Neighbor Embedding (tSNE; ref. ^37^) dimensionality reduction and FlowSOM clustering^38^. By doing so, it was possible to distinguish three populations depending on the expression of all the integrins in our panel (Fig. S2E). Overall, the PDX#3 model has a higher integrin expression level of all the considered subunits. By overlaying the β1-integrin and α2-integrin signal intensity to this clustering, we were able to visualize the higher surface expression of β1-integrin and α2-integrin in PDX#3 (Fig. S2F). Next, we extracted the normalized surface expression values of β1- and α2-integrins for all the independent events. The histograms further demonstrated a marked variation in the cell-surface integrin intensity and higher surface β1- and α2-integrin expression in the PDX#3 model compared to PDX#1 and PDX#2 (Fig. 2B).

To characterize the impact of the ex vivo culture on integrin surface expression, we performed a similar mass cytometry analysis on tumoroids cultured for 3 days in suspension or embedded in collagen. These data recapitulated the integrin expression profiles of the cells directly extracted from the PDX tumors mentioned above (Fig. S2G). When plotting the corresponding histograms (Figs. 2C and S2H), we observed that collagen embedding of the PDX#3 tumoroids clearly increased the surface expression of β1- and α2-integrins in the cell population, in line with their ability to revert polarity in collagen (Fig. 2C). This prompted us to test the outcome of integrin inhibition on PDX#3 tumoroid polarity following embedding in collagen. Blocking both integrins using established function-blocking antibodies (AIIB2 for β1-integrin and P1E6 for α2-integrin)^39,40^ prevented the polarity reversion of PDX#3 and PDO#2 in collagen (Fig. 2D–G) and collagen-induced Src phosphorylation in PDX#3 (Fig. 2H). Furthermore, the outcome of integrin inhibition was evident in abolished tumoroid–collagen interactions and in a circular tumoroid morphology in live-imaging experiments (Fig. S2I, J, Supplementary Movie 2). Taken together, these data indicate that α2β1-integrin-mediated cell interaction with collagen-I, and the ensuing downstream signaling, are required for the phenotypic polarity reversion in at least some MUC CRCs. However, given the limited number of models examined, these findings cannot yet be generalized to all MUC CRC patients.

### Polarity reversion is linked to altered expression of integrin traffic regulators

**Fig. 3:**
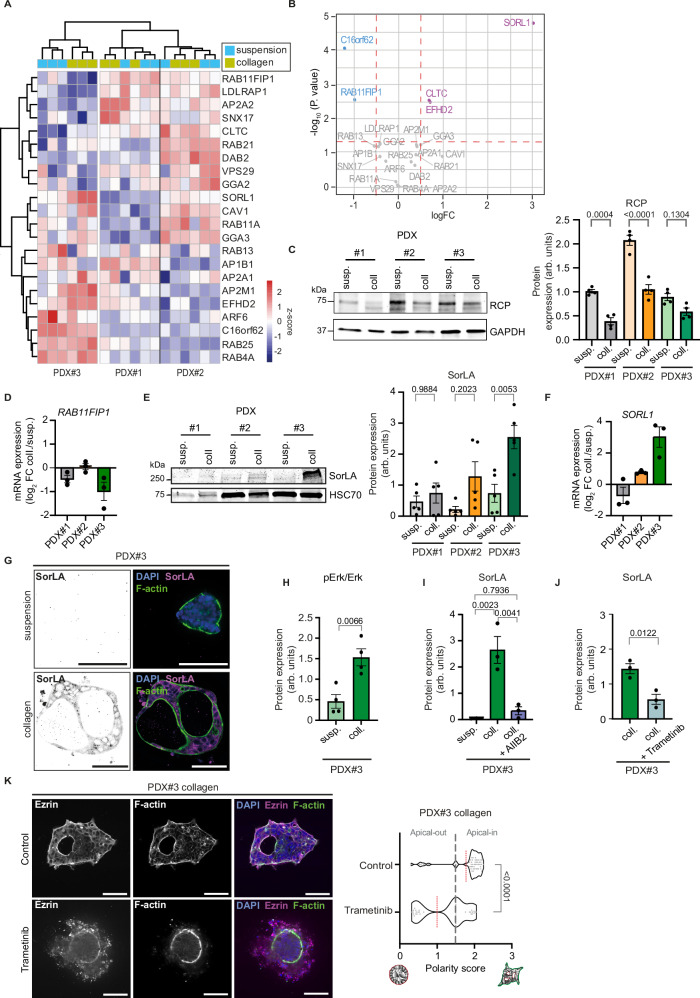
Key β1-integrin trafficking regulators are differentially expressed among PDX models and matrix conditions. **A** Relative expression of key regulators in β1-integrin trafficking, in PDX#1-, PDX#2-, and PDX#3-generated tumoroids in suspension and in collagen. **B** Volcano plot generated for the same geneset comparing PDX#3-generated tumoroids in collagen vs. suspension. Significantly upregulated genes in collagen are in purple, downregulated genes are in blue. See source data for exact *p* values. **C** Western blot analysis of RCP and GAPDH (loading control) and corresponding quantifications of RCP in PDX#1-, PDX#2-, and PDX#3-generated tumoroids in suspension and in collagen, normalized to the loading control (mean ± s.e.m; *n* = 4 biological replicates; One-way ANOVA with Tukey’s multiple comparison test). **D** Log_2_ fold change (FC) in *RAB11FIP1* (gene encoding RCP) mRNA expression in collagen vs. suspension from mRNA microarray analysis of PDX#1-, PDX#2-, and PDX#3-generated tumoroids (mean ± s.e.m; *n* = 3 biological replicates). **E** Western blot analysis of SorLA and HSC70 (loading control) and corresponding quantifications of SorLA in PDX#1-, PDX#2-, and PDX#3-generated tumoroids in suspension and in collagen normalized to loading control (mean ± s.e.m; *n* = 4 biological replicates; One-way ANOVA with Tukey’s multiple comparison test). **F** Log_2_ fold change in *SORL1* mRNA expression in collagen vs. suspension based on mRNA microarray analysis of PDX#1-, PDX#2-, and PDX#3-generated tumoroids (mean ± s.e.m; *n* = 3 biological replicates). **G** Immunofluorescence of PDX#3-generated tumoroids in suspension and in collagen, stained for SorLA (*n* = two biological replicates). Scale bars, 100 μm. **H** Western blot quantifications of ERK phosphorylation ratio in PDX#3 in suspension and collagen (mean ± s.e.m; n = 4 biological replicates; unpaired t-test with Welch’s correction). **I** Western blot quantifications of SorLA expression in PDX#3 in suspension and collagen ± AIIB2 (mean ± s.e.m; *n* = 3 biological replicates; One-way ANOVA with Tukey’s multiple comparison test). **J** Western blot quantification of SorLA expression in PDX#3-generated tumoroids in collagen ± trametinib (250 nM) (mean ± s.e.m; *n* = 3 biological replicates; two-tailed unpaired t-test with Welch’s correction). **K** Representative immunofluorescence images of PDX#3-generated tumoroids in collagen ± trametinib, stained for Ezrin, F-actin, and DAPI. Scale bars 10 μm. Corresponding polarity score of PDX#3-generated tumoroids in collagen ± trametinib. Data are displayed as violin plots with mean indicated by a red line. Dashed line (at polarity score 1.5) indicates the mid-point between apical-in and apical-out characteristics [*n* = 52 (control) and 43 (trametinib); two-tailed Mann–Whitney test]. Source data provided.

Whole exome sequencing (SRA number: PRJNA1473190) revealed no mutations in* ITGB1* and *ITGA2* genes across the three PDXs (Fig. S2K), excluding a loss of protein function in PDX#1/PDX#2 as a potential explanation for the observed differences between the models. Cell-surface integrin levels are influenced not only by gene transcription but also by the dynamic process of integrin traffic, the continuous endocytosis of integrins from the plasma membrane, and their return (recycling)^35,41,42^. To investigate the potential role of integrin trafficking in our models, we generated a list of 22 genes known to regulate this process and examined their expression in the same transcriptome data set (GSE152299) (Fig. 3A). PCA representation of this data (Fig. S3A) demonstrated an effect of matrix conditions on PDX#3 compared to PDX#1 and PDX#2. A Volcano plot representation of the geneset (Fig. 3B) identified two interesting targets for further investigation: *RAB11FIP1* and *SORL1*.

*RAB11FIP1* encodes a Rab coupling protein (RCP; also called Rab11fip1, Rab11 family-interacting protein 1) that has been implicated in numerous studies as an important positive regulator of β1-integrin recycling, cancer cell invasion, and metastasis^43–45^. *SORL1* encodes the SorLA protein, a cell-surface sorting receptor implicated in trafficking of APP (Amyloid precursor protein) in neurons and rapid recycling of β1-integrins in breast cancer^46,47^. To validate the transcriptome data, we analyzed RCP and SorLA protein levels in the PDX tumoroids in suspension and collagen. RCP was downregulated upon collagen embedding on the protein level in all of the tumoroids and on the mRNA level in PDX#1 and PDX#3 (Fig. 3C, D). In contrast, *SORL1*/SorLA mRNA and protein expression were significantly upregulated only in collagen-embedded PDX#3 tumoroids (Figs. 3E–G and S3B), which prompted us to specifically focus on this target. In breast cancer cells, integrin–ECM engagement activates the MAPK/ERK signaling pathway, and ERK activity positively regulates *SORL1* transcription^48,49^, which could also be a possible mechanism for collagen-induced upregulation of *SORL1*/SorLA in mucinous CRC. Indeed, we detected significantly elevated ERK phosphorylation in PDX#3 tumoroids in collagen (Figs. 3H and S3C), and collagen-induced SorLA upregulation was sensitive to both integrin function-blocking antibodies (Figs. 3I and S3D) and MEK/ERK inhibitor, trametinib (Figs. 3J and S3E, F). In addition, trametinib-treated PDX#3 tumoroids showed significantly reduced polarity reversion and were surrounded by dead cells (Fig. 3K). These data suggest the possibility that the polarity switching of PDX#3 tumoroids in collagen is supported by ERK-induced SorLA expression.

### Apical-in polarity in collagen is established through SorLA-dependent β1-integrin recycling

**Fig. 4:**
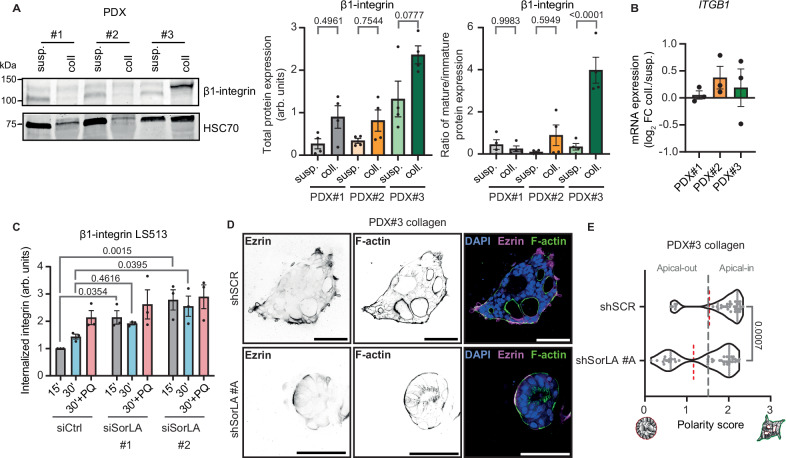
SorLA-mediated β1-integrin trafficking is necessary for polarity reversion in collagen. **A** Western blot and corresponding quantifications and maturation ratios of β1-integrin in PDX#1-, PDX#2-, and PDX#3-generated tumoroids in suspension and in collagen (mean ± s.e.m; *n* = 4 biological replicates; One-way ANOVA with Tukey’s multiple comparison test). **B** *ITGB1* mRNA expression fold change in collagen vs. suspension generated from an RNA microarray analysis of PDX#1-, PDX#2-, and PDX#3-generated tumoroids (mean ± s.e.m; *n* = 3 biological replicates). **C** β1-integrin uptake assay in LS513 after siRNA-induced silencing of SorLA. Timepoints are 15′ and 30′, and the recycling inhibitor used is primaquine (PQ) (mean ± s.e.m; *n* = 3 biological replicates; One-way ANOVA and Dunnett’s multiple comparisons test). **D** Immunofluorescence of PDX#1-, PDX#2-, and PDX#3-generated tumoroids in suspension and in collagen, showing polarity status through Ezrin staining after SorLA KD by shRNA. **E** Comparison of polarity scores of PDX#3 tumoroids in collagen after SorLA silencing. Data are displayed as violin plots with mean indicated by a red line. Dashed line (at polarity score 1.5) indicates the mid-point between apical-in and apical-out characteristics (*n*_shSCR_ = 38, *n*_shSORLA #A_ = 44; two-tailed Mann–Whitney test). Scale bars, 100 μm. Source data provided.

To explore SorLA-mediated β1-integrin regulation, we first investigated the impact of collagen embedding on β1-integrin total protein and *ITGB1* mRNA levels (Fig. 4A, B). Western blot analysis of β1-integrin typically reveals two bands, a slower-migrating band representing the mature, fully-glycosylated protein, and a faster-migrating band, indicating the immature, ER-resident protein. The relative abundance of these two protein forms has been linked to alterations in integrin traffic, with a higher ratio of the slower-migrating band corresponding to increased β1-integrin recycling and plasma membrane residency time^50^. Interestingly, overall β1-integrin levels were not affected in any of the three PDX tumoroids in response to collagen-I. However, quantification of the ratio of the mature versus immature protein form revealed a significant increase in integrin maturation in the PDX#3 model in response to collagen-I. The identity of the slower-migrating band as mature β1-integrin was further confirmed with digestion of the lysates with PNGase (Fig. S4A).

To formally assess the role of SorLA in integrin recycling within MUC CRC cells, we sought to establish a more tractable cell-based model, given the technical challenges associated with performing cell biology with PDX models. Similar to some other cell lines^51^, the MUC CRC cell line LS513 generates buds from confluent cultures and releases TSIPs in the medium. We observed that these cells phenocopy all the key features of the PDX#3 tumoroids. Once embedded in collagen, the LS513-generated TSIPs formed lumens, shifted significantly towards an apical-in polarity (Fig. S4B, C), and upregulated SorLA levels (Fig. S4D). We concluded that these cells are a good surrogate to study β1-integrin traffic. Previous work has shown that β1-integrins recycle to the plasma membrane with a turnover rate of 10–15 min^52–54^. Impaired recycling can lead to the gradual intracellular accumulation of β1-integrin^55^. We silenced *SORL1* in LS513 with two independent siRNA oligos (Fig. S4E) and used the gold-standard cell-surface biotinylation-based integrin uptake assay to monitor endocytosed β1-integrin levels^56,57^. *SORL1* silencing significantly elevated intracellular β1-integrin levels within 15 min, mirroring the effects of the recycling inhibitor primaquine (Fig. 4C), suggesting that loss of SorLA reduces integrin recycling, similar to our previous findings in breast cancer^47^.

To further explore the role of SorLA in collagen-induced polarity reversion, we established a stable tumoroid line (PDXO) from PDX#3 tumor samples. We observed increasing SorLA levels upon collagen embedding during 72 h (Fig. S4F) and detected overlapping vesicular punctae of α2-integrin and SorLA (Fig. S4G). To functionally test the requirement for SorLA, we silenced *SORL1* using lentiviral shRNA. We obtained more than 50% silencing (Fig. S4H) and observed a significant reduction in PDX#3 polarity reversion in collagen-I, with clusters retaining a mostly apical-out polarity (Fig. 4D, E), indicating that SorLA is necessary for the apical-in polarity in collagen.

### SorLA-dependent β1-integrin trafficking is induced by HER2/HER3 signaling

The human epidermal growth factor receptor 2 (HER2) is a transmembrane receptor tyrosine kinase (RTK) implicated in breast and gastroesophageal cancers, where targeted therapies against this RTK have already been developed for clinical use^58^. Recently, HER2-targeted drugs have also been approved by the FDA for HER2-positive CRC^59^. We have shown that SorLA interacts with HER2 homo- and HER2/HER3 heterodimers, supporting rapid HER2/HER3 recycling and protein stability and oncogenic signaling in breast and bladder cancers^47,48^. Furthermore, Heregulin-β1-induced HER2/HER3 signaling increases SorLA transcription^48^. To explore whether RTK signaling is implicated in MUC CRC polarity, we analyzed HER2 and HER3 expression in PDX#1, PDX#2, and PDX#3 tumoroids in suspension and collagen. Although HER2 is significantly upregulated in both PDX#2 and PDX#3 in collagen, HER3 induction was more variable and reached significance only in the collagen-responsive PDX#3 (Fig. S5A, B). Immunofluorescence staining further validated these results with clear HER2 and HER3 signal detected in PDX#3 in collagen, where HER2 mostly localized at the basal pole (Fig. S5C). Similar to the collagen-induced SorLA expression, HER2 and HER3 upregulation were also integrin dependent in PDX#3 in collagen (Fig. S5D).

**Fig. 5:**
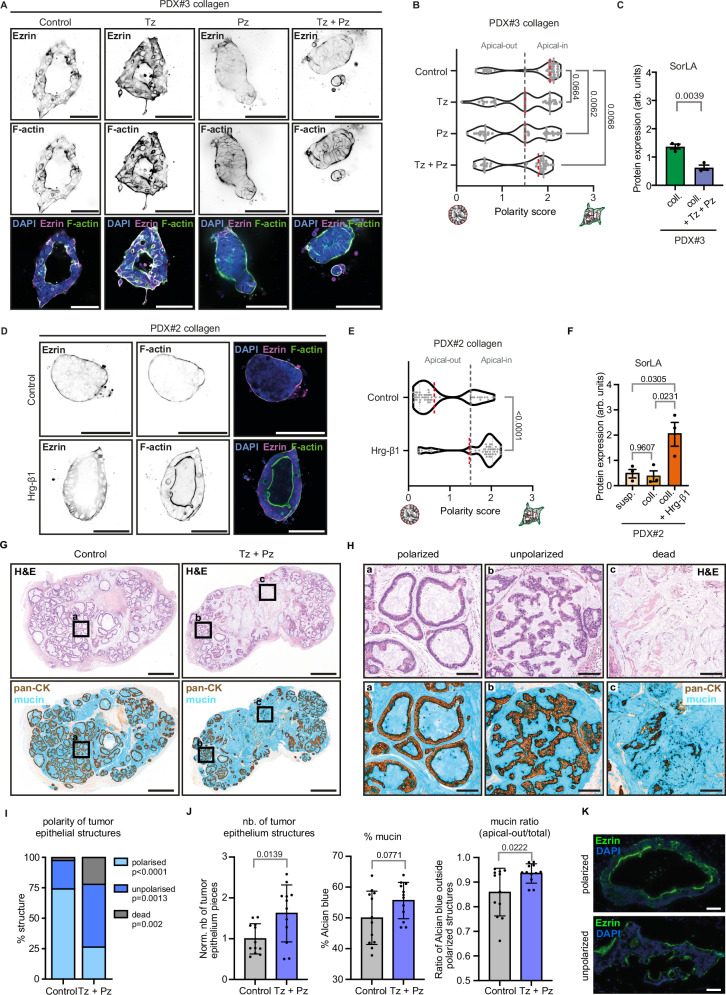
HER2 and HER3 control apicobasal polarity orientation. **A** Immunofluorescence of PDX#3-generated tumoroids in suspension, collagen, and in collagen treated with a HER2-blocking antibody (trastuzumab, Tz, 10 μg/ml), a HER2/HER3 heterodimerization-blocking antibody (pertuzumab, Pz, 10 μg/ml), or a combination of both (Tz+Pz). Polarity status is depicted through Ezrin staining. Scale bars, 100 μm. **B** Comparison of polarity scores in PDX#3 in collagen with HER2/HER3-blocking antibodies. Data are displayed as violin plots with mean indicated by a red line. Dashed line (at polarity score 1.5) indicates the mid-point between apical-in and apical-out characteristics (*n*_Ctrl_ = 44, *n*_Tz_ = 39, *n*_Pz_ = 47, *n*_Tz+Pz_ = 38; Kruskal–Wallis test with Dunn’s multiple comparisons test). **C** Western blot quantification of SorLA expression in PDX#3-generated tumoroids in collagen ± a combination of trastuzumab and pertuzumab (Tz+Pz; both 10 μg/ml) (mean ± s.e.m; *n* = 3 biological replicates; two-tailed unpaired t-test with Welch’s correction). **D** Immunofluorescence of PDX#2-generated tumoroids in suspension and in collagen ± a HER3 ligand (Heregulin-β1, Hrg-β1, 20 ng/ml), showing polarity status through Ezrin staining. **E** Comparison of polarity scores in PDX#2 in collagen ± Hrg-β1. Data are displayed as violin plots with mean indicated by a red line. Dashed line (at polarity score 1.5) indicates the mid-point between apical-in and apical-out characteristics (*n*_Ctrl_ = 56, *n*_Hrg-β1_ = 62; two-tailed Mann–Whitney test). **F** Western blot quantifications of SorLA expression in PDX#2 in suspension and in collagen ± Hrg-β1 (mean ± s.e.m; *n* = 3 biological replicates; One-way ANOVA with Tukey’s multiple comparison test). Representative images (**G**) and zoomed-in insets (**H**) of Hematoxylin & Eosin (H&E), Alcian Blue, and Pan-Cytokeratin (Pan-CK) stainings in PDX#3 tumors extracted from the flank of control vs treated (Tz+Pz, 10 mg/kg) mice. Scale bar, 1.5 mm. Insets illustrate the structures characterized as polarized, unpolarized, and dead tumor epithelium based on the stainings. Insets on the Alcian blue images show the segmentation results used in quantitative analysis (see Fig. S6B). Scale bar 150 µm. **I** Qualitative classification of polarized, unpolarized, and dead tumor epithelial structures in control and treated tumors, shown as a percentage of the whole tumor. Statistical analysis was performed between the conditions for each indicated feature (*n* = 12 tumor sections out of four tumors/group; two-tailed unpaired t-test with Welch’s correction). **J** Segmentation-based quantification of the number of epithelial structures (Pan-CK), percentage of mucin (Alcian blue), and the ratio of apical-out mucin (mucin outside the closed tumor epithelial structures) comparing control and treated tumors (mean ± SD; *n* = 12; two-tailed unpaired t-test with Welch’s correction). **K** Representative images of polarized and unpolarized tumor epithelial structures based on Ezrin immunofluorescence staining (*n* = 4 tumors/group). Scale bar, 50 µm. Source data provided.

To assess the impact of HER2 and HER3 signaling on tumoroid polarity, we used two monoclonal antibodies: pertuzumab (antibody binding to the dimerization arm of HER2) and trastuzumab (antibody that binds to a different epitope of HER2), both in clinical use for HER2-positive breast cancer treatment^60^. In our setup, trastuzumab alone had no significant effect on PDX#3 polarity. However, pertuzumab alone or in combination with trastuzumab significantly shifted PDX#3 tumoroids toward an apical-out polarity (Fig. 5A, B), indicating a functional role for HER2/HER3 signaling in PDX#3 polarity reversion in response to collagen. Furthermore, SorLA expression was significantly downregulated in PDX#3 upon HER2/HER3 inhibition (Figs. 5C and S5E). Nevertheless, HER2/HER3 activation with the HER3 ligand Heregulin-β1 was sufficient to trigger collagen-induced polarity reversion in the thus far matrix-agnostic PDX#2 tumoroids (Fig. 5D, E). Upon HER3 stimulation with Heregulin-β1, SorLA was also found to be upregulated in PDX#2 (Figs. 5F and S5F). Using a different set of CRC PDOs, we confirmed that Heregulin-β1 treatment was sufficient to trigger the apical-out to apical-in polarity switch while HER2/HER3-inhibiting antibodies prevented collagen-induced polarity reversion (Fig. S5G, H). The PDX#1 tumoroids did not respond, presumably due to their lack of HER3 expression (Fig. S5I, A). Additionally, whole exome sequencing (Fig. S5J) indicated that there are no mutations in the aforementioned genes, *RAB11FIP1*, *SORL1*, *HER2*, and *HER3*, except for a missense mutation in HER2 in PDX#2 (a G>A at nucleotide 1943 encoding Arg648Gln), which SIFT and PolyPhen scores suggest is benign on its structure and function^61,62^.

### HER2/HER3 regulates PDX polarity in vivo

To test the relevance of the HER2/HER3 signaling axis in vivo, we treated PDX#3 tumor-bearing mice with a combination of trastuzumab and pertuzumab (both 10 mg/kg) or control IgG1 antibody. Histological analyses revealed that antibody treatment disrupted the organization of tumor epithelial structures (Fig. 5G, H) by markedly reducing the proportion of polarized epithelial structures and increasing necrotic areas (Figs. 5I and S6A). Quantitative image analysis confirmed the increase of tumor epithelium fragments, indicating loss of polarity upon HER2/HER3 inhibition. Furthermore, there was a higher ratio of mucin outside polarized structures, suggesting reversion from apical-in to apical-out polarity in the treated tumors (Figs. 5J and S6B). Interestingly, the overall tumor burden and the area of tumor epithelium were not significantly affected by the treatment (Fig. S6C, D). Immunofluorescence staining for Ezrin further verified the HER2/HER3 inhibition induced loss of apical-in polarity in the affected regions (Fig. 5K). Taken together, these data indicate that HER2/HER3 signaling maintains apical-in polarity in vivo and that inhibition of this pathway promotes polarity loss and cell death. However, we cannot exclude the possibility of additional factors contributing to this response.

### Apical-in CRC polarity is associated with enhanced collagen remodeling

**Fig. 6:**
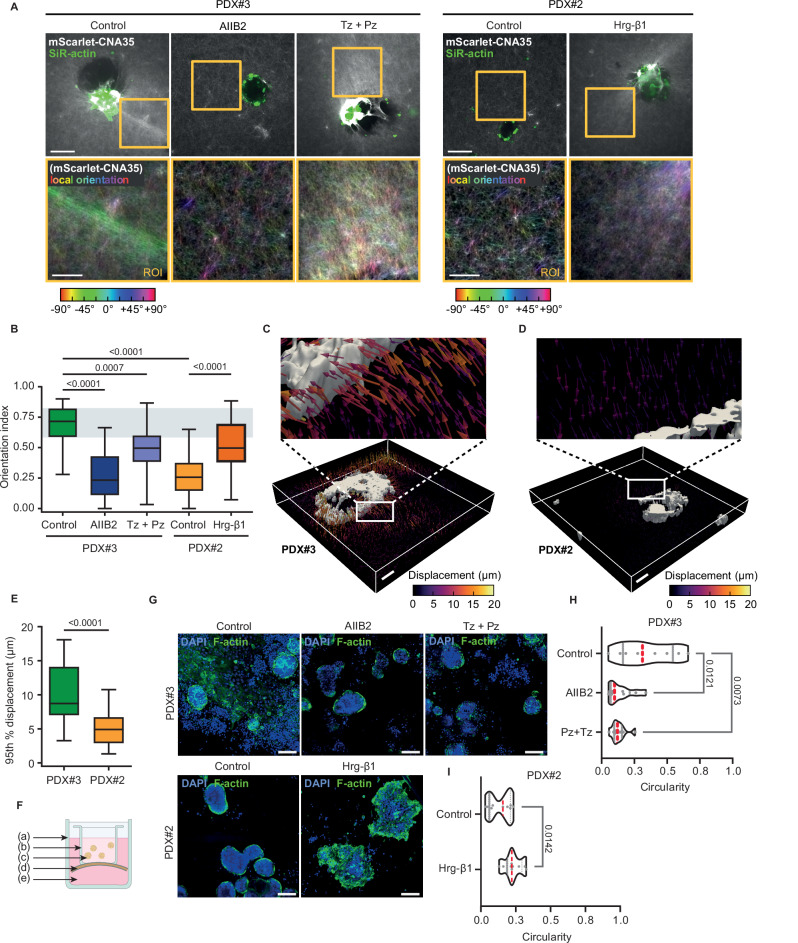
Collagen remodeling is correlated to apicobasal polarity status in collagen. **A** Visualization of collagen fiber orientations proximal to PDX#2 and PDX#3 tumoroids treated with β1-integrin-blocking antibody (AIIB2), HER2/HER3-blocking antibodies (Pz+Tz), and HER3 ligand (Hrg-β1). Collagen fibers are marked with mScarlet-CNA35, and the images are color-coded based on the local orientation of the fibers. Scale bars, 50 μm (main), 20 μm (inset). **B** Collagen fiber orientation index in PDX#2 and PDX#3 treated as indicated (*n*_PDX#3 Ctrl_ = 28, *n*_PDX#3 AIIB2_ = 28, *n*_PDX#3 Pz+Tz_ = 21, *n*_PDX#2 Ctrl_ = 29, *n*_Hrg-β1_ = 23; One-way ANOVA with Šidák’s multiple comparison test). Box plot shows the median, interquartile range (box bounds), and minimum and maximum values. **C** Visualization of reversible displacements exerted on the collagen matrix by a PDX#3 tumoroid (rendered in the image using thresholded F-actin signal). **D** Visualization of reversible displacements exerted on the collagen matrix by a PDX#2 tumoroid. **E** Quantification of the matrix displacements exerted by the PDX#3 and PDX#2 tumoroids (*n*_PDX#3_ = 27, *n*_PDX#2_ = 29; two-tailed unpaired t-test with Welch’s correction). Box plot shows the median, interquartile range (box bounds), and minimum and maximum values. **F** Setup for the peritoneum invasion assay: (a): well of a 12-well cell culture plate, (b) Transwell cell invasion insert, from which the membrane has been removed, (c) tumoroids, (d) decellularized peritoneum, (e) medium. **G** Fluorescence imaging of PDX#2- and PDX#3-generated tumoroids after 6 days of cell contact with the decellularized peritoneum, after treatment with AIIB2 (1 μg/ml), Pertuzumab+Trastuzumab (Tz+Pz, 10 μg/ml, 10 μg/ml) or Hrg-β1 (20 ng/ml). **H** Comparison of polarity scores in PDX#3 in collagen after treatment with AIIB2 (1 μg/ml) or Pertuzumab+Trastuzumab (Tz+Pz, 10 μg/ml each) (*n* = 9 spheres selected randomly; One-way ANOVA with Tukey’s multiple comparison test). **I** Comparison of circularity in PDX#2 in collagen after treatment with Heregulin-β1 (Hrg-β1, 20 ng/ml) (*n* = 9 spheres selected randomly; two-tailed Mann–Whitney test). Scale bars, 100 μm (main), 50 μm (3D projections). Vector scaling is set to 200% for clarity. Source data provided.

Since the reversion of PDX#3 tumoroid polarity in collagen was dependent on direct integrin-mediated cell-ECM interactions, we wanted to investigate whether this would also lead to concomitant remodeling of the ECM itself. To study the mechanics of the SorLA/HER2/HER3-dependent polarity reversion, we visualized collagen fiber rearrangements using a fluorescent probe specific for fibrillar collagen (mScarlet-conjugated CNA35)^63^. The PDX#3 tumoroids remodeled the collagen matrix, resulting in increased fiber alignment in the tumoroid-proximal ECM (Figs. 6A, B and S7A), whereas integrin inhibition with AIIB2 antibody or dual inhibition of HER2/HER3, with the combination of pertuzumab and trastuzumab, significantly decreased matrix alignment (Figs. 6A, B and S7A). Conversely, treating PDX#2 tumoroids with Heregulin-β1 increased collagen fiber orientation, indicating elevated collagen remodeling (Figs. 6A, B and S7B).

To further investigate biophysical interaction between the cells and their ECM, we imaged collagen-embedded PDX#3 and PDX#2 tumoroids before and after Latrunculin B treatment. Latrunculin B disrupts the actin cytoskeleton, releasing cellular tension and leading to a recoil in the tensed matrix (Figs. 6C, D and S7C). Comparing the images before and after treatment allowed us to compute the reversible displacement of collagen fibers caused by tumoroid contraction in each condition. Using a previously described MATLAB pipeline^64^, we observed significantly higher reversible collagen displacements proximal to the PDX#3 tumoroids compared to PDX#2 (Fig. 6C–E). This suggests that the collagen fibers are under constant mechanical tension exerted by the MUC CRC cells, and such forces are much higher in the apical-in tumoroids.

### HER2/HER3 and integrins regulate CRC tumoroid interaction with the peritoneum ex vivo

CRC often metastasizes to the liver and peritoneum, and successful metastasis depends on the cancer cells’ ability to adhere to and invade this tissue^65^. To study CRC interaction with the peritoneal basement membrane with high granularity and in response to various treatments, we set up an in vitro model. We isolated and decellularized mouse peritoneum, a technique previously applied to the mouse mesentery^66^, which was shown to recapitulate the organization and characteristics of the in vivo basement membrane^67^. We plated the PDX tumoroids on the decellularized peritoneum (Fig. 6F) and tested the outcome of different treatments (AIIB2 or Trastuzumab+Pertuzumab for PDX#3, Heregulin-β1 for PDX#2) on the ability of the tumoroids to adhere and spread on the tissue. Despite the relatively low adhesion rate, we observed tumor spheres adhering to and spreading on the peritoneum matrix (Fig. 6G), and, using actin staining, we quantified their circularity. The PDX#3 tumoroids, which revert their polarity to apical-in when embedded in collagen, spread extensively on the peritoneum with individual cells dissociating from the clusters. Integrin- and HER2/HER3-inhibiting antibodies significantly blocked this (Fig. 6G, H). In contrast, the untreated PDX#2 tumoroids did not display extensive spreading on the peritoneum. However, stimulation with the HER3 ligand Heregulin-β1 enhanced spreading and reduced spheroid circularity (Fig. 6G–I).

Taken together, these data are concordant with a model where HER2 and HER3 regulate integrin-–collagen interactions in MUC CRC tumoroids, and increased integrin cell surface availability and signaling are associated with both apical-in polarity and altered tumoroid-proximal ECM architecture.

### High HER2/HER3/SORLA expression correlates with apical-in polarity of CRC tumors

**Fig. 7:**
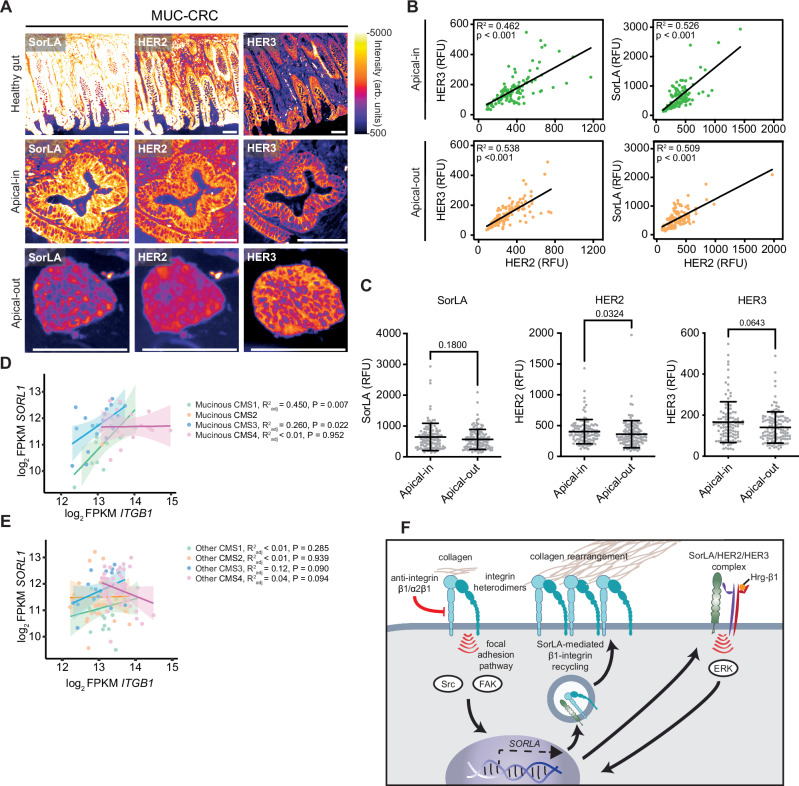
HER2, HER3, and SorLA expressions are correlated in vivo. **A** Immunohistochemistry of healthy gut tissue, apical-in and apical-out cancer structures from MUC CRC patients stained for HER2, HER3, and SorLA. These panels have been made from two consecutive different slides that were cut at different depths of the tumor, which explains the slight change in structure between the pictures. Correlation plots (from a simple linear regression, two-tailed Pearson’s correlation coefficient) (**B**) and expression (**C**) for HER2, HER3, and SorLA in a cohort of 25 patients (5 apical-in and 5 apical-out structures chosen for each patient when possible) in apical-in and apical-out cancer structures. (mean ± SD; HER2: *N*_Apical-in_ = 115, *N*_Apical-out_ = 120, two-tailed Mann–Whitney test; HER3: *N*_Apical-in_ = 110, *N*_Apical-out_ = 120, Mann–Whitney test; SorLA: *N*_Apical-in_ = 115, *N*_Apical-out_ = 120, Mann–Whitney test). **D** Correlation (from a simple linear regression) between *ITGB1* and *SORL1* expression in mucinous CRCs of CMS1-4 (log_2_ of Fragments per Kilobase of transcript per Million mapped reads (FPKM) plotted). Shaded areas represent the 95% confidence interval for the mean estimate. **E** Correlation between *ITGB1* and *SORL1* expression in non-mucinous CRCs of CMS1-4 (log_2_ FPKM). Shaded areas represent the 95% confidence interval for the mean estimate. Statistical significance was assessed using two-sided t-tests. **F** Proposed mechanism of collagen-induced FAK and Src activation and SorLA expression, leading to β1-integrin recycling. This mechanism is supported by a HER2/HER3/SorLA positive feedback loop and results in efficient matrix-sensing and collagen rearrangement. Scale bars, 100 μm. Source data provided.

To determine if the ex vivo findings on SorLA, HER2, and HER3 were applicable in vivo, we investigated their correlation in PDX tumors in mice. We observed high HER2 staining and predominantly apical-in polarity in the PDX#3 tumors. Conversely, the staining intensity was low in the predominantly apical-out PDX#2 tumors (Fig. S8A). Encouraged by the concordant results between in vivo and ex vivo models regarding HER2, we stained a cohort of 25 human MUC CRC patient samples^68^ for these three proteins. The staining revealed a strong positive signal for HER2/SorLA and HER2/HER3 in the normal epithelium as well as the epithelium of apical-in CRC clusters, whereas in apical-out structures, the staining intensities of SorLA and HER2 were lower (Figs. 7A and S8B). The quantifications revealed interesting findings: first, there is a positive correlation between HER2 and HER3, as well as between HER2 and SorLA, both in apical-in and apical-out structures (Fig. 7B). Second, there is a modest correlation between the polarity status and HER2 expression (higher in apical-in than in apical-out). Although similar tendencies were seen for HER3 and SorLA, the lack of statistical significance did not allow us to draw any strong conclusions (Fig. 7C).

Interestingly, by analyzing gene expression profiles from a TCGA CRC cohort with prior histological characterization^69^, and dividing the cohort into two groups (mucinous and non-mucinous) (Fig. S8C), we were able to see a positive correlation between *ITGB1* and *SORL1* mRNA expression both in CMS1 and CMS3 (Consensus Molecular Subtype 1 and 3) solely for MUC CRCs (CMS2 being mostly non-represented in the mucinous CRCs), which was not the case for the non-mucinous CRCs (Fig. 7D, E).

Cumulatively, using cell- and mechanobiology methods to interrogate MUC CRC PDX tumors ex vivo and patient samples, we report a previously uncharacterized polarity determination mechanism whereby HER2/HER3/SorLA-controlled β1-integrin traffic controls tumor–ECM interactions and ECM rearrangements (Fig. 7F).

## Discussion

Epithelial polarity is often considered a feature of normal tissue, maintained by cell–cell and cell–basement membrane interactions, and one that is progressively lost in cancer in conjunction with increased invasion and metastasis. Our study uncovers a dynamic crosstalk between integrins, HER-family RTKs, and receptor membrane traffic orchestrated by SorLA that jointly determine MUC CRC polarity. Using cell- and mechanobiology methods to interrogate ex vivo CRC PDX tumoroids and PDO spheres and patient samples, we report a previously uncharacterized polarity determination mechanism whereby HER2/HER3/SorLA-controlled β1-integrin traffic regulates tumor–ECM interactions and ECM remodeling. Furthermore, expression of these receptors shows significant positive correlation in clinical patient specimens (Fig. 7B). While our data show that perturbation of these pathways inhibits collagen-induced polarity reversal, the molecular mechanisms underpinning polarity reversal may be more complex and diverse.

In normal and cancer cells, integrins are constantly trafficked from the plasma membrane to endosomes and recycled back to facilitate dynamic cell adhesion^35,70^. Increased integrin traffic facilitates invasion and metastasis in many cancer types, including breast and pancreatic cancer^41,71^. In particular, increased integrin recycling via the trafficking protein RCP has been linked to β1-integrin recycling in complex with EGFR to drive cancer cell motility in a 3D matrix^43,45,72–74^. On the other hand, SorLA, a well-established sorting protein regulating membrane traffic of APP in neurons and insulin receptor in adipocytes, has been shown to regulate integrin and HER2/HER3 receptor recycling in HER2-dependent breast cancer^47,48,75–80^. Here we find that MUC CRC tumor spheres, interacting with collagen, significantly downregulate RCP expression and upregulate SorLA. This implies that upon polarity reversion, TSIPs’ integrin recycling is altered to a receptor trafficking program that maintains polarity and supports HER2/HER3 signaling. To the best of our knowledge, SorLA has not been previously implicated in CRC or in the regulation of cell polarity. These data increase our understanding of MUC CRC cluster behavior during cancer progression and are likely to have important functional implications, as previous work has indicated that non-reverted CRC clusters are capable of long-distance collective ameboid migration and are more resistant to chemotherapy^18,22^. In this study, we focused our investigation on PDXs and PDOs with only apical-in or apical-out phenotypes in order to decipher the underpinning mechanism of ECM-based polarity reversion and the features distinguishing these distinct polarities. However, in a cohort including 36 MUC CRC patients^21^, it was observed that most tumors show marked heterogeneity and generate both apical-in and apical-out PDOs. These may reflect either genetic diversity within the tumor, spatial heterogeneity in the microenvironment, or plasticity of polarity at different stages of tumor development; however, this remains to be investigated in the future.

We observed that efficient polarity reversion and apical-in morphology facilitates MUC CRC interaction with decellularized mouse peritoneum and promotes local collagen remodeling, inducing collagen fiber alignment adjacent to the tumoroids. Similar radially organized ECM signatures have been shown to drive local invasion and are associated with poor patient outcomes in breast cancer^81,82^, suggesting that the impact of polarity reversion in MUC CRC may be multifaceted, and its role during the different phases of the metastatic cascade and in different tissue microenvironments warrants careful investigation.

Integrin expression is frequently altered in CRC, with laminin-binding α6β4-integrin being frequently overexpressed and perhaps the most broadly studied integrin heterodimer^83^. Of the collagen-binding integrins, α1β1-integrin is normally restricted to the crypt epithelium but is upregulated in 65% of CRCs, where its expression is driven by MYC^84,85^. Surprisingly, the main fibrillar collagen-binding integrin-α2β1 has not been investigated extensively in CRC. We find here that both α1β1- and α2β1-integrins are expressed in the PDX tumors and tumor spheres, with α2β1 having the highest expression in the apical-in polarized PDX and PDO models. Inhibitory antibodies for α2-integrin are sufficient to block collagen-induced polarity reversion, implying that α1β1-integrin–collagen interaction alone is not supportive of polarity reversion. This is an interesting distinction that could be linked to the pro-tumorigenic role of α1β1-integrin in CRC and warrants further investigation in the future.

Both a combination of a tyrosine kinase inhibitor tucatinib plus trastuzumab, as well as the antibody drug-conjugate trastuzumab deruxtecan, have recently been approved by FDA as therapies for HER2-positive CRC^86,87^. Interestingly, HER2 amplifications and elevated levels of the HER3 ligand, Heregulin-β1, are linked to resistance of CRC to the anti-EGFR therapeutic antibody cetuximab^88,89^. As expected, distinct CRC subtypes show differential responses to cancer therapeutics owing to their different biology. Here, we find a correlation between *ITGB1* and *SORL1* expressions solely in the mucinous subtypes of CRC, which shows the presented mechanism might be limited to specific histological phenotypes of cancer. Our discovery outlining the polarity regulation of MUC CRC by HER2/HER3 signaling ex vivo and in vivo, as well as in human tumors, suggests a functionally important role for the pathway in this CRC subtype, which may warrant further attention in the field of CRC therapy.

## Methods

### Ethical considerations

Animal experiments: Animal experiments performed in France were compliant with French legislation and EU Directive 2010/63. The project was validated by the Ethical Committee (CEEA) no. 26 and was then granted French government authorizations under numbers 517-2015042114005883 and 2734-2015111711418501. Animal experiments performed in Finland were in accordance with the Finnish Act on Animal Experimentation (animal license numbers ESAVI/12558/2021 and ESAVI/6253/2024). Mice were obtained from Charles River France and Germany, housed and bred at the Gustave Roussy animal core facility (accreditation number E-94-076-11) and at TCDM (Turku Center for Disease Modelling). Mice were housed in standard conditions (12-h light/dark cycle; ambient temperature, 21 °C; 50% humidity ± 8%) with food and water available ad libitum. Animals were euthanized according to endpoints that were validated by the Ethical Committee, the French government (Ministère de l’Enseignement Supérieur, de la Recherche et de l’Innovation), and the Finnish government (Etelä-Suomen Aluehallintovirasto, hankelupatoimikunta).

Patient samples: The PDOs used in this study were initially derived from fresh tumor tissue samples collected within two precision medicine studies: ORGANOTREAT (NCT05267912) and Gustave Roussy Cancer Profiling STING trial (NCT04932525), both approved by the Gustave Roussy institutional review board and ethics committee^90,91^, in compliance with the Declaration of Helsinki. All patients gave signed and informed consent for tumor sampling and collection and for genomic analysis.

### Definitions

*Sphere*: cluster of tumor cells.

*Tumor Sphere with Inverted Polarity (TSIP)*: specific cluster of tumor cells showing enrichment of apical domain markers at its peripheral surface and basolateral markers on its internal cell-cell contacts.

*Tumoroid*: small pieces of patient-derived xenograft (PDX) tumors maintained no more than 3 days in culture.

*Organoid*: cell line of tumor cells maintained as spheres.

*Patient-Derived Xenograft Organoid (PDXO)*: PDX-derived cell line maintained as spheres.

*Patient-Derived Organoid (PDO)*: cell line derived from freshly collected patient tumors and maintained as cell clusters.

### Culture and passaging of patient-derived samples

Three human colorectal tumors (PDX#1 corresponding to LRB-0009C, PDX#2 corresponding to GR-0012P, and PDX#3 corresponding to IGR-014P) from the previously described CReMEC tumor collection initiative^29^ were maintained in NSG mice (strain: NOD.Cg-*Prkdc*^*scid*^ *Il2rg*^*tm1WjI*^/SzJ, female, 6–8 weeks old at grafting)^21,29^. Briefly, small tumor fragments were subcutaneously engrafted on both flanks of anesthetized mice (2.5% isoflurane). Tumor growth was measured once to twice a week. When the combined tumor burden reached 1700 mm^3^, the mice were euthanized, tumors were used for ex vivo experiments, and 50 mm^3^ fragments were engrafted on the flanks of new mice in order to maintain the biological material source. The maximal tumor burden was not exceeded during experiments.

### Generation of tumoroids

Tumoroids were prepared according to ref. ^21^ and adapted for muco-secreting tumors as follows. The PDX#1, PDX#2 or PDX#3 tumors of 1200–1700 mm^3^ were retrieved from the mice, minced into small fragments using a sterile scalpel and incubated for 1 h at 37 °C in a final volume of 5–10 ml of culture medium (Dulbecco’s modified Eagle’s medium; DMEM) without fetal bovine serum (FBS) and with 2 mg/ml collagenase (Sigma, C2139). The samples were then mixed with 20 ml of DMEM, filtered through 100 μm mesh size cell strainers (Greiner, EASYstrainer, 542000), and then centrifuged at 277 g for 10 min. Clusters were isolated from the remaining mucin and single cells by washing in 10 ml of DMEM and pulse centrifuging at 277 × *g* five times. The clusters, now free of mucin and single cells, were maintained for 3 days in ultra-low attachment plates (Corning, CLS3471) in culture medium (DMEM supplemented with 10% FBS). Tumoroids were then pelleted at 277 × *g* and used for further experiments.

### Generation of PDX-derived organoid (PDXO)

The clusters obtained from the PDX as described previously were pulse centrifuged at 277 × *g*, resuspended in Matrigel (Corning, 354230), and plated in 10 × 15 μl droplets in the bottom of a 6-well plate (Greiner, 657160). Cells were then incubated at 37 °C for 15 min to let the basement membrane extract polymerize. During polymerization, the cell culture plate was placed upside down to prevent the PDXOs from settling at the bottom. Following polymerization, culture medium was added as described in ref. ^92^, without any human R-spondin1, A83-01, and Afamin-Wnt-3A in serum-free medium. During the first 2 days, the organoid expansion medium was supplemented with Y27632 (Calbiochem, 688000, 10 μM). This medium was renewed every 2 days, and PDXOs were passaged every 7–14 days as described^90^. For experiments, the well was first washed thrice with PBS before adding 1 ml of Cell Recovery Solution (Corning 354253). Cells were then left to incubate at 4 °C for 20 min, and mechanical dissociation was performed with a p1000 pipette until the Matrigel was completely dissolved. PBS (4 ml) was added, and spheroids were pulse centrifuged at 277 × *g*. The spheres, now free of Matrigel, were maintained for 3 days in ultra-low attachment plates (Corning, CLS3471) in DMEM supplemented with 10% FBS. Organoids were then pelleted at 277 × *g* and used for further experiments.

### Generation of patient-derived organoid (PDOs)

The PDOs used in this study were initially derived from fresh tumor tissue samples collected within two precision medicine studies (ORGANOTREAT, NCT05267912; STING, NCT04932525)^90,91^. They were maintained in culture as described^90,91^. Briefly, PDOs were passaged every 6–10 days by dissociation using TrypLE 1X (Thermo Fisher Scientific) and mechanical pipetting, followed by pelleting (350 g for 5 min) twice, resuspension in basement membrane extract (BME; Culturex UltiMatrix Reduced Growth Factor Basement Membrane Extract, Culturex, BME001-10; or Matrigel® Matrix for Organoid Culture Phenol-Red Free, Corning, 356255) at 500 cells/μl, and plating. PDOs were then cultured at 37 °C in organoid culture medium modified from ref. ^93^ supplemented with Intesticult™ Organoid Growth Medium (Stemcell™ Technologies, 06010), referred to as “PDO media,” replenished three times per week. Only after BME polymerization, 10 μM Y27632 (Selleckchem, S1049) was added. For experiments, PDOs were retrieved from BME by a brief 1 min TrypLE 1X digestion, filtered on a 70-µm strainer (EASYStrainer, 542070), washed twice in full DMEM, and incubated in “PDO media” in a petri dish (non-culture-coated) at 37 °C for 1 day. For collagen embedding, PDO#1 and PDO#2 were recovered from Matrigel after 5 days of culture by successive washes with cold medium and subsequently transferred to a 6-well ultra-low attachment plate for 2 days.

### LS513 culture and LS513 TSIPs formation

LS513 cells were obtained from ATCC (#CRL-2134) and cultured in RPMI-1640 medium supplemented with 10% FBS in 10 cm cell culture dishes. To generate TSIPs from the LS513 monolayer, the medium was changed every 2 days until the monolayer reached confluence. After waiting 5 days, the medium was collected and pulse centrifuged at 277 × *g* to collect the LS513 TSIPs. These were left for 3 days in suspension in ultra-low attachment plates (Corning, CLS3471), and embedded in collagen as described earlier (using the LS513 medium). For passaging, the monolayer was digested with 1x trypsin when 70% confluence was reached.

### Collagen embedding

Collagen-I (Corning, 354236) was neutralized with 1 M NaOH and 10× MEM (Life Technologies, 21430-02) according to the ratio: 1:0.032:0.1 (v/v/v). The collagen concentration was then adjusted to 2 mg/ml with 1x DMEM, and incubated on ice for 1 h. The wells of a µ-Slide 8-Well ibiTreat slide (Ibidi, 80826) or 6-well plate (Greiner, 657160) were first pre-coated with 7 µl or 70 µl of the neutralized collagen-I for 30 min at 37 °C to allow collagen polymerization. The PDXOs and PDOs, after 3 or 2 days in suspension, respectively, were mixed with neutralized collagen-I at a concentration of 30–50 organoids of PDXOs/5 µl and 100 organoids of PDOs/5 µl. Then, 30 µl of organoid-collagen-I solution was added on top of the pre-coated wells. For the 6-well plates, 300 µl of organoid-collagen-I solution was added on top of the pre-coated wells. The gel was allowed to polymerize for 1 h at 37 °C. Organoids were then cultured in culture medium supplemented with 10% FBS for 3 days (PDX#3, PDO#1, and PDO#2) or 6 days (PDX#1 and PDX#2). The drugs were diluted in the medium as follows: AIIB2 (DSHB, AB528306, 1 μg/ml), Heregulin-β1 (Peprotech, 100-03-10UG, 20 ng/ml), Trastuzumab (Herceptin, Roche, 10 μg/ml), Pertuzumab (Perjeta, Genentech, 10 μg/ml), Saracatinib (Selleckchem, S1006, 1 μM), EHT-1864 (R&D Systems, 3872, 5 μM), P1E6 (DSHB, AB2619597, 10 μg/ml), FAK14 (Tocris, 3414, 10 μM), Trametinib (MCE, HY-10999, 250 nM, 48 h treatment).

### Ex vivo peritoneum assay

Peritoneum samples were collected from mice and decellularized by incubating them in a 1 M NH_4_OH solution for 1 h at RT. After washing thrice with PBS for 15 min, peritoneum samples were left to incubate with PBS and penicillin-streptomycin (1:100) at 4 °C overnight. After washing thrice with PBS for 15 min, the peritoneum was sectioned into 1 cm × 1 cm pieces and adhered (using Tissue Adhesive, 3 M, 1469SB) to the bottoms of plastic Transwell inserts (Greiner, Thincerts, 8 um pore size, 662638) after removing the filter with a scalpel. 100 tumor spheres were resuspended in 100 ml of DMEM + 10% FBS and placed in the well with either AIIB2 (DSHB, AB528306, 1 μg/ml), Y27632 (Calbiochem, 688000, 25 μM), Heregulin-β1 (Peprotech, 100-03-10UG, 20 ng/ml), orTrastuzumab (Herceptin, Roche, 10 μg/ml) alone or combined with Pertuzumab (Perjeta, Genentech, 10 μg/ml) for 6 days. The fixing and immunofluorescencestaining were performed as described previously. Peritoneum bits were placed upside down on glass-bottom dishes (Cellvis, D35-14-1-N), and imaged as described previously, using the ×20 objective.

### Immunofluorescence staining

After incubation for 3 days in suspension or for 3–6 days in collagen, the apico-basolateral polarity of organoids was quantified. Cells were washed thrice with PBSCM (PBS supplemented with CaCl_2_ (0.1 mM) and MgCl_2_ (1 mM)), fixed with 4% paraformaldehyde (PFA) for 5 min (for spheres in suspension) or 45 min (for spheres in collagen) at RT. Spheres fixed in suspension were then embedded in collagen for imaging as previously described. Permeabilization was then performed in PBSCM supplemented with 0.5% Triton X-100 for 45 min. Spheres were incubated with primary antibodies overnight at 4 °C with the dilutions mentioned in Supplementary Table 2 in PBSCM supplemented with 10% FBS and 0.1% Triton X-100. After washing thrice with PBSCM supplemented with 0.05% Tween, spheres were incubated with secondary antibodies and phalloidin for 2 h at room temperature (RT) with the dilutions mentioned in Supplementary Table 2, as well as with DAPI (1 μg/ml). Spheres were then washed thrice with PBSCM supplemented with 0.05% Tween. The gel was then immersed in PBS before imaging.

### Confocal imaging

Images were acquired either using a SpinningDisk CSU-W1 microscope (Yokogawa) with a ZylasCMOC camera piloted with an Olympus X83, or with a 3i CSU-W1 spinning disk confocal microscope with Hamamatsu ORCA-Flash4.0 v2 scientific complementary metal-oxide semiconductor (sCMOS) camera (x40 water immersion objective, 1.1 NA). For traction force microscopy experiments and live imaging, a x20 objective was used. Basic image processing, including look-up table optimization, channels overlay, projections, etc., was performed using Fiji software^94^. More specific quantifications are detailed in the following sections. In panels showing Ezrin staining to assess the polarity phenotype, the signal balance was adjusted for a better visualization. These adjustments were only done on representative shown images and not on the image data used for quantitative analysis.

### Polarity score

The computation of the polarity score (PS) used throughout the manuscript is based on three parameters (S1A), defining three sub-scores: Sub-score (1), the presence/absence of lumen (*L*), set to 0 in case of lumen absence, or 1 in case of the presence of one or more lumen. Sub-score (2), circularity (*C*), obtained through the shape descriptor function on ImageJ. A value of 0 would be obtained for a perfect sphere, whereas a value closer to 1 translates to a higher number of protrusions. The segmentation here is based on the actin fluorescence signal. Sub-score (3), the apicobasal Ezrin fluorescence signal ratio (*F*), which is the ratio of Ezrin luminal signal (or the mean of luminal signals in case of multiple lumens, *F*_luminal_) to total Ezrin fluorescence (*F*_luminal_+*F*_cortical_). This value varies from 0 (complete cortical signal) to 1 (complete luminal signal). The sum of these three sub-scores is the total polarity score (PS) varying from 0 (perfect apical-out) to 3 (perfect apical-in). In case of non-polarized structures (cytoplasmic Ezrin signal), we set the polarity score to 1.5 per default.

1 $${PS}=L+C+\frac{{F}_{{{\rm{luminal}}}}}{{F}_{{{\rm{luminal}}}}+{F}_{{{\rm{cortical}}}}}$$

### Western blotting

Spheres embedded in collagen were first released from the matrix by incubation in DMEM without serum supplemented with 2 mg/ml collagenase (Sigma, C2139) for 45 min. After pulse centrifuging at 277 × *g*, spheres were washed with PBS and pulse centrifuged at 277 × *g* twice. Spheres were then lysed in TXLB buffer [50 mM Hepes, 1% Triton X-100, 0.5% sodium deoxycholate, 0.1% SDS, 0.5 mM EDTA, 50 mM NaF, 10 mM Na_3_VO_4_, and protease inhibitor cocktail (cOmplete Mini, EDTA-free, Roche)]. Cells cultured in 2D were washed twice with PBS and directly lysed with TXLB. Separation was performed by gel electrophoresis (Mini-PROTEAN TGX Precast Gels 4-20%, Bio-Rad, 4561096), before transferring onto a nitrocellulose membrane (Trans-Blot Turbo Transfer System, Bio-Rad) and blocking with AdvanBlock-Fluor (Advansta, R-03729-E10). Primary antibodies in AdvanBlock-Fluor were incubated overnight at 4 °C with the dilutions mentioned in Supplementary Table 2. Membranes were washed thrice between primary and secondary antibody treatments with Tris-buffered saline with 0.1% Tween 20 (TBST). IRDye secondary antibodies (see Supplementary Table 2) were incubated for at least 1 h at RT, before detection on an Odyssey fluorescence imager CLx (LI-COR). Densitometry analysis was performed in Fiji by normalizing the signal to a suitable loading control (either GAPDH, β-actin, or HSC70).

### PNGase digestion of lysates

For PNGase digestion, cell lysates were prepared in SDS-free buffer [50 mM Hepes, 1% NP-40, 0.5% sodium deoxycholate, 0.5 mM EDTA, 50 mM NaF, 10 mM Na_3_VO_4_, and protease inhibitor cocktail (cOmplete Mini, EDTA-free, Roche)]. 9 μl of lysate was mixed to 1 μl of Glycoprotein Denaturing Buffer (10X) (NEB, B1704S). The lysate was denatured at 100 °C for 10 min, then chilled on ice. 2 μl GlycoBuffer 2 (10X) (NEB, B3704S), 2 μl 10% NP-40 (NEB, B2704S), 5 μl H_2_O, and 1 μl PNGase F (NEB, P0704S) were then added to the lysate, and mixed gently. The lysate was then incubated at 37 °C for 1 h. From there, the samples were prepared and run as described before.

### *SORL1* silencing using shRNA lentiviral transduction

During passaging of the PDX#3-generated PDO line as described previously, 10^5^ cells were resuspended in 36 μl of organoid expansion medium and infected with 4 M TU [62 μl of each virus (Origene, TL309181V)], 1 μl polybrene (Merck, TR-1003-G, stock solution at 1 mg/ml) and 1 μl Y-27362 (Calbiochem, 688000, 10 μM, stock solution 1 mM), in a U-bottom 96-well plate (Falcon, 351177) for 6 h at 37 °C. The well’s contents were then collected in a 1.5 mL Eppendorf tube, 1 ml of DMEM + 10% FBS was added, and cells were centrifugated at 200 × *g* for 3 min. Cells were then resuspended in 60 μl Matrigel (Corning, 354230) and 4×15 μl droplets were poured in a 24-well cell culture plate (Cellstar, 662 160) and left at 37 °C to polymerize for 15 min. 1 ml organoid expansion medium was used for organoid expansion (supplemented with 10 μM Y-27362 for the first 2 days). The medium was changed every 2 days, and organoids were left to grow for 7 days. For polarity assays, the well was first washed thrice with PBS before adding 1 ml of Cell Recovery Solution (Corning 354253). Cells were then left to incubate at 4 °C for 20 min, and mechanical dissociation was performed with a p1000 pipette until Matrigel was completely dissolved. 4 ml of PBS was added, and spheroids were pulse centrifuged at 277 × *g*. The spheres, now free of Matrigel, were maintained for 3 days in ultra-low attachment plates (Corning, CLS3471) in PDX culture medium. Then, organoids were pelleted at 277 × *g* and used for further experiments.

### *SORL1* silencing using siRNA transient transfection

LS513 were plated in a 6-well plate at 80% confluency. Transient siRNA transfections were performed using Lipofectamine RNAiMAX reagent (Invitrogen, 56532) according to the manufacturer’s instructions. *SORL1*-targeting siRNAs were ON-TARGETplus obtained from Dharmacon—siSORLA #1 (J-004722-08), siSORLA #2 (J-004722-06), siSORLA #3 (J-004722-07), siSORLA #4 (J-004722-05). For controls, Allstars negative control (Qiagen, Cat. No. 1027281) was used. siRNA concentrations used were all 20 nM and cells were transfected with siRNAs 72 h prior to experiments.

### Integrin uptake assay

Surface biotinylation-based integrin trafficking assays in *SORL1*-silenced LS513 cells were performed based on previously published methods^56,57^, with some modifications. Nunc MaxiSorb 96-well plates (Thermo Fischer, 44-2404-21) Enzyme-linked immunosorbent assay (ELISA) plates were coated with anti-β1-integrin antibody mix (5 μg/ml of AIIB2 (in-house) and anti-CD29 (BD Bioscience #610468)) in TBS (50 μl per well) overnight at +4 °C. The wells were blocked with 5% BSA in TBS for 2 h at 37 °C (100 μl per well). LS513 cells were silenced 3 days before the experiment as described earlier. 2 h prior to the experiment, the medium was changed to pre-warmed RPMI with 10% FBS to induce receptor traffic. The cells were placed on ice and washed once with cold PBS. Cell surface proteins were labeled with 0.13 mg/ml EZ-link cleavable sulfo-NHS-SS-biotin (Thermo Scientific, 21331) in serum-free RPMI medium for 30 min at +4 °C. Any unbound biotin was removed by washing with cold medium and pre-warmed RPMI + 10% FBS with or without 100 μM primaquine (Sigma, 160393) was added to the cells. The biotin-labeled surface proteins were allowed to traffic at +37 °C for 15 or 30 min. Cells were placed on ice, washed once with cold PBS, and cold cell surface reduction buffer (50 mm Tris-HCl, pH 8.6 and 100 mm NaCl). Cell surface biotin was cleaved with non-membrane permeant reducing reagent MesNa (30 mg/ml, sodium 2-mercaptoethanesulfonate; Fluka, 63705) in cell surface reduction buffer for 20 min at 4 °C, followed by quenching with 100 mM iodoacetamide (Sigma, I3750) for 15 min on ice. For the 0 min internalization, cells were maintained on ice in serum-free RPMI until cell surface reduction with MesNA. The cells were lysed by scraping in lysis buffer (1.5% octylglucoside, 1% NP-40, 0.5% BSA, 1 mM EDTA, and protease and phosphatase inhibitors) and incubation at +4 °C for 20 min with rotation and cleared by centrifugation (16,000 × *g*, 10 min, 4 °C). To detect the biotinylated integrins, 50 μl volumes of the cell lysates were incubated in duplicate wells at +4 °C overnight, washed extensively with TBST, incubated for 2 h at 4 °C with 1:1000 horseradish peroxidase-coupled streptavidin (Fisher, 21130), washed, and detected with antibody for ELISA detection.

### Trastuzumab and pertuzumab treatment of PDX#3 in vivo

Mice were grafted with PDX#3 tumor fragments on both flanks (*n* = 4). Tumor fragments were soaked in a pretreatment solution of a combination of trastuzumab (Herceptin, Roche, 50 µg/ml in PBS) and pertuzumab (Perjeta, Genentech, 50 µg/ml in PBS) or non-targeting control IgG1 antibody (#BE0297, BioXCell, 100 µg/ml in PBS) prior to grafting. Mice received intraperitoneal injections twice a week for 3 weeks with either combination of trastuzumab and pertuzumab (both 10 mg/kg) or control IgG1 antibody (20 mg/kg). Tumor volumes and body weights were measured twice a week throughout the experiment.

After 3 weeks of treatment, mice were sacrificed, and tumors were collected for histological analysis: fixed in 4% buffered formaldehyde (10.5000.0001, Q Path) for 24 h at +4 °C, washed in PBS, embedded in paraffin, and sectioned. Three sections per tumor with 100 µm trimming in between each were used for quantification (*n* = 12 per condition). Classical histology protocols were used for Hematoxylin-Eosin and Alcian blue stainings. Immunohistochemistry staining was performed for pan-cytokeratin and cleaved caspase-3, and immunofluorescence staining for Ezrin (see Supplementary Table 2). Slides were imaged using Panoramic 1000 Digital Scanner (3dHistech) and Panoramic MIDI FL (3dHistec) for Ezrin staining. Manual analysis to qualify the nature of each tissue epithelial structure was performed using QuPath 6.0 software. For segmentation-based analysis, images were converted to TIFF format using QuPath and analyzed in ImageJ with the Labkit plugin^95^. Tumor epithelial structures (pan-CK, mucins (Alcian blue)) and the rest of the tissue were segmented to calculate the percentages and ratios quantified. In both cases, the origin of the images was blinded to the analyst and revealed after quantification.

### Patient-sample immunohistochemistry

Histological CRC and peritoneum specimens obtained after surgical resection were formalin-fixed and paraffin-embedded according to routine protocols. Sections (3 μm) of formalin-fixed and paraffin-embedded samples were deparaffinized, unmasked (pH 8), and rehydrated before immunohistochemistry or immunofluorescence stainings. Immunohistochemistry sections were immunostained for SorLA, HER2, and HER3 (see Supplementary Table 2). Slides were imaged using Axioscan Z1, Zeiss (×20, NA 0.8). Due to differences in the three different stains, imaging was optimized separately for each channel, but the settings were then kept constant for all samples. Regions of interest (ROIs) were hand-drawn using QuPath^96^, exported as TIFF files through custom scripting, and then stored as Zarr datasets. These were subsequently fed into a custom processing pipeline using the Python scientific library scikit-image^97^. Zone binary masks for apical-in and apical-out ROIs were generated using digital image processing. For apical-out ROIs, zero-intensity pixels were used to delineate the mask boundary; for apical-in ROIs, a combination of Gaussian filter, local thresholding, and binary morphological operations was used, with masks drawn manually when required. Within the masked zones, nuclear counts based on the DAPI channel were generated using a StarDist^98,99^ pre-trained model “2d_versatile_fluo.” The total intensity signal for SorLA, HER2, and HER3 was measured in apical-in and apical-out ROIs and normalized against the nuclear count in respective ROIs. Pearson’s test was used for correlation analysis between HER2, HER3, and SorLA.

### Mass cytometry

Spheroids grown in collagen for 3/6 days underwent collagen digestion by incubation in DMEM without serum supplemented with 2 mg/ml collagenase (Sigma, C2139) for 45 min. After resuspending in DMEM + 10% FBS and pulse centrifugating at 277 × *g*, they were collected. Spheroids cultured in suspension, clusters obtained from PDX digestion, or spheroids released from collagen as described previously were washed with PBS thrice and pulsed centrifuged at 277 × *g* thrice in order to keep the clusters/spheroids and get rid of any single cells and secreted mucins. The spheres were then digested in Cell Dissociation Buffer Enzyme-Free PBS-based (Gibco, 13151-014) supplemented with 2 mg/ml collagenase (Sigma, C2139) and incubated for 1 h at 37 °C with occasional mechanical dissociation by pipetting. After addition of DMEM + 10% FBS to quench the collagenase, cells were centrifuged at 200 × *g* for 3 min and washed thrice with PBS. They were then resuspended in 1 ml cold PBS and filtered through a 5 ml polystyrene round-bottom tube with Cell-Strainer cap (Falcon, 352235), and kept on ice until staining, which was done according to the Fluidigm Maxpar Cell Surface Staining with Fresh Fix protocol. The sample was then run using a Helios™ Mass Cytometer, and the data were analyzed with Cytobank and clustered/visualized through SPADE and tSNE analysis.

### Generation of fluorescent collagen

To fluorescently label rat tail type I collagen (~4.24 mg/mL, 354236, Corning), 1.65 mL was mixed with 450 μL of Milli-Q water and 500 μL of neutralizing buffer (20 mM NaH_2_PO_4_, 112 mM Na_2_HPO_4_⋅2H_2_O, 0.4 M NaCl, and 46 mM NaOH) and incubated at 37 °C for 30 min. The polymerized collagen was then washed thrice with PBS for 10 min. Then, 3 mL of Milli-Q water and 1 mL of bicarbonate buffer [1 M NaHCO_3_ (pH 8), raised dropwise to pH 8.3 using 1.17 M Na_2_CO_3_ (pH 11)] were added to the collagen gel before addition of the Alexa Fluor™ 647 NHS Ester (Succinimidyl Ester) dye (A20006, Invitrogen) in 100 μL of PBS. After incubating the collagen mix overnight at 4 °C, the dye was then removed, and the collagen was washed with PBS with rotation at RT for 30 min, changing the PBS five times. Stained collagen was then depolymerized through the addition of 2 mL HCl (20 mM) and gentle rotation at 4 °C overnight. The collagen was finally centrifuged at 20,000 × *g* for 10 min, collecting the labeled collagen from the supernatant. The fluorescent collagen was then used at a 1:1000 concentration in the previously described neutralized collagen gel.

### Cloning and generation of CNA35

To generate the CNA35-mScarlet construct, pET28a-CNA35-EGFP (a kind gift from Maarten Merkx (Eindhoven University of Technology, MB Eindhoven, The Netherlands), Addgene plasmid #61603) was digested with NheI/EcoRI and ligated with an NheI/EcoRI digested mScarlet gene fragment (IDT) to generate pET28a-mScarlet-CNA35. The final construct was validated by analytical digestion and sequencing. Recombinant protein production and purification for CNA35-mScarlet was performed as described previously^100^.

### Whole exome sequencing

DNA was extracted using the DNeasy kit (Qiagen, Cat. No. 69504) from organoids either after 3 days in suspension (washed once in PBS supplemented with Ca^2+^ and Mg^2+^ as mentioned above). Whole exome analysis was performed by Integragen SA (France), comparing the three samples (PDX#1, PDX#2, and PDX#3) to a PON (panel of normal), and analyzed with MERCURY™. Whole exome sequencing (WES) data have been deposited in the NCBI Sequence Read Archive (SRA) under accession number PRJNA1473190.

### Collagen orientation analysis

Type I collagen gels with PDX#3 and PDX#2 spheroids were prepared on glass-bottom dishes (Cellvis, D35-14-1-N). 80 µl of PureCol EZ Gel (Advanced BioMatrix, 5074) was spread on the glass bottom using a micropipette tip and allowed to polymerize at +37 °C for 1 h. Next, PDX#3 or PDX#2 spheroids were pulse centrifuged at 277 × *g* to remove the mucin and single cells. Approximately 100 spheroids were mixed with 80 µl of PureCol EZ Gel and pipetted on top of the previously polymerized collagen layer, after which the mixture was allowed to polymerize at +37 °C for 1 h. Spheres were treated with AIIB2 (DSHB, AB528306, 1 μg/ml), Heregulin-β1 (Peprotech, 100-03-10UG, 20 ng/ml), Trastuzumab (Herceptin, Roche, 10 μg/ml), and/or Pertuzumab (Perjeta, Genentech, 10 μg/ml). One day before the samples were imaged, the cultures were supplemented with 1:1000 SiR-actin (Sprichrome, SC001) and ~40 µg/ml of mScarlet-conjugated collagen probe CNA35^63^ (see above for details on cloning).

The spheroids were imaged live using a Marianas spinning disk confocal microscope, 20x objective, Orca Flash4 sCMOS camera, and 2 × 2 binning (see Microscopy for details) at 37 °C, 5% CO_2_. 60–80 µm stacks were acquired around the center (z) of each spheroid. To analyze collagen fiber orientation around the spheroids, ~4 µm substacks were acquired near the center of each spheroid, from which maximum intensity projections were prepared. Next, 200 × 200 µm regions of interest depicting CNA35 directly proximal to each spheroid (but excluding any dense collagen aggregates on the spheroid surface) were selected from the projections for analysis. If the matrix surrounding the spheroid was obviously heterogeneous, the region was selected to maximize the local alignment.

The selected regions were analyzed with ImageJ plugin OrientationJ, using cubic spline gradient and a local window size of 4 pixels. In the color survey, hue represented orientation, and saturation represented coherence. All the local orientations were exported and analyzed in R to yield fiber orientation indices^101,102^. Briefly, the orientations (−90°…+90°) representing each region were normalized, i.e., their distribution was centered around zero based on the peak of the histogram. Next, orientation indices (*S*) were calculated such that

2 $$S=2 < {\cos }^{2}\alpha > -1$$

where *α* is the angle between an individual (fiber) orientation and the average orientation of the entire region, and <cos^2^*α*> is the average square cosine of all *α* per analyzed region. An index of 0 represents a random distribution, and an index of 1 represents a perfectly aligned distribution.

### Collagen displacement fields

To measure transient displacements exerted on the collagen matrix by the PDX#3 and PDX#2 spheroids, the spheroids were prepared and embedded in type I collagen, as described above. The spheroids were grown in the gels for 6 days and supplemented with 1:1000 SiR-actin and ~40 µg/ml mScarlet-CNA35 1 day before the imaging. 60–80 µm stacks were acquired around/near the center of each spheroid, before and after the cells and matrix were relaxed by adding 10 µM latrunculin B and incubating for 20 min. Mariana’s spinning disk confocal microscope, 20× objective, Orca Flash4 sCMOS camera, and 2×2 binning were used for the imaging (see Microscopy for details).

3D displacement fields were calculated using TFMLAB^64,103,104^, a traction force microscopy toolbox implemented in MATLAB R2022a (MathWorks). The spheroids were segmented using actin images, variable threshold adjustment, and a minimum object size of 10^4^ voxels. Rigid image registration was conducted using the default phase correlation-based algorithm. The displacements were calculated from CNA35 images using 10 × 10 × 10 µm grid spacing, default registration metric and optimizer (normalized correlation coefficient, adaptive stochastic gradient descent), and post-shift correction. The results were visualized using ParaView v5.11.0^105^.

### Analysis of *SORL1* and *ITGB1* gene expression in human tumors

Preprocessed TCGA colon adenocarcinoma cohort RNAseq data and raw RSEM-counts were downloaded from https://gdc.cancer.gov/node/905/ and https://gdac.broadinstitute.org/runs/stddata__2016_01_28/data/, respectively. CMSCaller^106^ was used to infer consensus molecular subtypes (CMS) from RSEM-counts, excluding calls with FDR > 0.05. The samples were categorized as mucinous and control cases based on previously conducted characterization^69^. Associations between *SORL1* and *ITGB1* gene expression were assessed with the preprocessed, normalized log2 mRNA expression data by computing linear regressions within each CMS group. Statistical significance was evaluated using two-sided t-tests of the regression coefficients.

### Microarray analysis

The microarray data are from a previous publication^21^ and are available on the Gene Expression Omnibus with the accession number GSE152299. An unsupervised classification was performed based on the expression of genes involved in ITGB1 membrane trafficking, curated from the literature. Normalized gene expression values were used to perform unsupervised hierarchical clustering using Euclidean distance and Ward.D2 linkage, and heatmaps were generated with the pheatmap R package with row scaling.

For differential expression analysis, the limma package was applied to the same ITGB1‑trafficking geneset, comparing cells cultured on collagen versus cells in suspension after filtration. Linear models were fitted with empirical Bayes moderation, and genes were considered significantly differentially expressed at a false discovery rate (FDR) ≤ 0.1 after a moderated t-test, and the Benjamini–Hochberg adjustment method was implemented in the R package limma. Volcano plots were generated from the limma output, showing log_2_ fold-change between collagen and suspension conditions on the x‑axis and the −log_10_ adjusted p‑value on the y‑axis, with significantly regulated genes highlighted.

### Statistics and reproducibility

GraphPad Prism (versions 9.3.1 and 10.1.1) was used for all statistical analyses. Data distribution was determined with the Shapiro-Wilk normality test. Two-sample testing was performed using Student’s t-test (unpaired, two-tailed) with Welch’s correction (normally distributed data) or nonparametric Mann–Whitney U-test (non-normally distributed data). Multiple comparisons were performed using either one-way ANOVA (normally distributed data) or Kruskal–Wallis (non-normally distributed data), followed by an appropriate post-hoc test as indicated in the figure legends. Data are presented as column graphs, dot plots (mean ± standard error; s.e.m), box plots (defined in the legends), or violin plots. *P* values less than 0.05 were considered to be statistically significant. Exact *p* values are provided in the figures where possible. Otherwise, *p* values are available in the statistical Source data for each figure.

No statistical methods were used to predetermine sample sizes. Data were reproduced in three or more biological replicates unless otherwise indicated in the figure legends.

The experiments were not randomized and were not performed in a blinded fashion, with the exception of the analysis of the in vivo models of PDX#3 tumors treated with trastuzumab and pertuzumab or IgG control. In this case, the analyst was blind to the treatment condition. Analysis software/statistical packages were used as detailed in the methods for robust data analysis, removing user bias. In addition, appropriate controls were included in experiments, and control versus treated samples were analyzed in the same fashion.

### Reporting summary

Further information on research design is available in the Nature Portfolio Reporting Summary linked to this article.

## Supplementary information

Supplementary Information

Description of Additional Supplementary Files

Supplementary Movie 1

Supplementary Movie 2

Reporting Summary

Transparent Peer Review file

## Source data

Source data

## Data Availability

Data supporting the findings of this study are available within the paper and its Supplementary Information files or from the corresponding authors upon reasonable request. The microarray data are from a previous publication^21^ and are available on the Gene Expression Omnibus with the accession number GSE152299. WES data have been deposited in the NCBI Sequence Read Archive (SRA) under accession number PRJNA1473190. Source data are provided with this paper.

## Code availability

The custom image processing pipeline used to analyze the patient IHC data is available on Zenodo (10.5281/zenodo.20156260).
