# Supplementary material for: Signaling downstream of tumor–stroma interaction regulates mucinous colorectal adenocarcinoma apicobasal polarity
Source: Nat Commun. 2026 Jul 4;17:8303. doi: 10.1038/s41467-026-75127-0 (PMC13469138; doi:10.1038/s41467-026-75127-0)

Figure 2 H

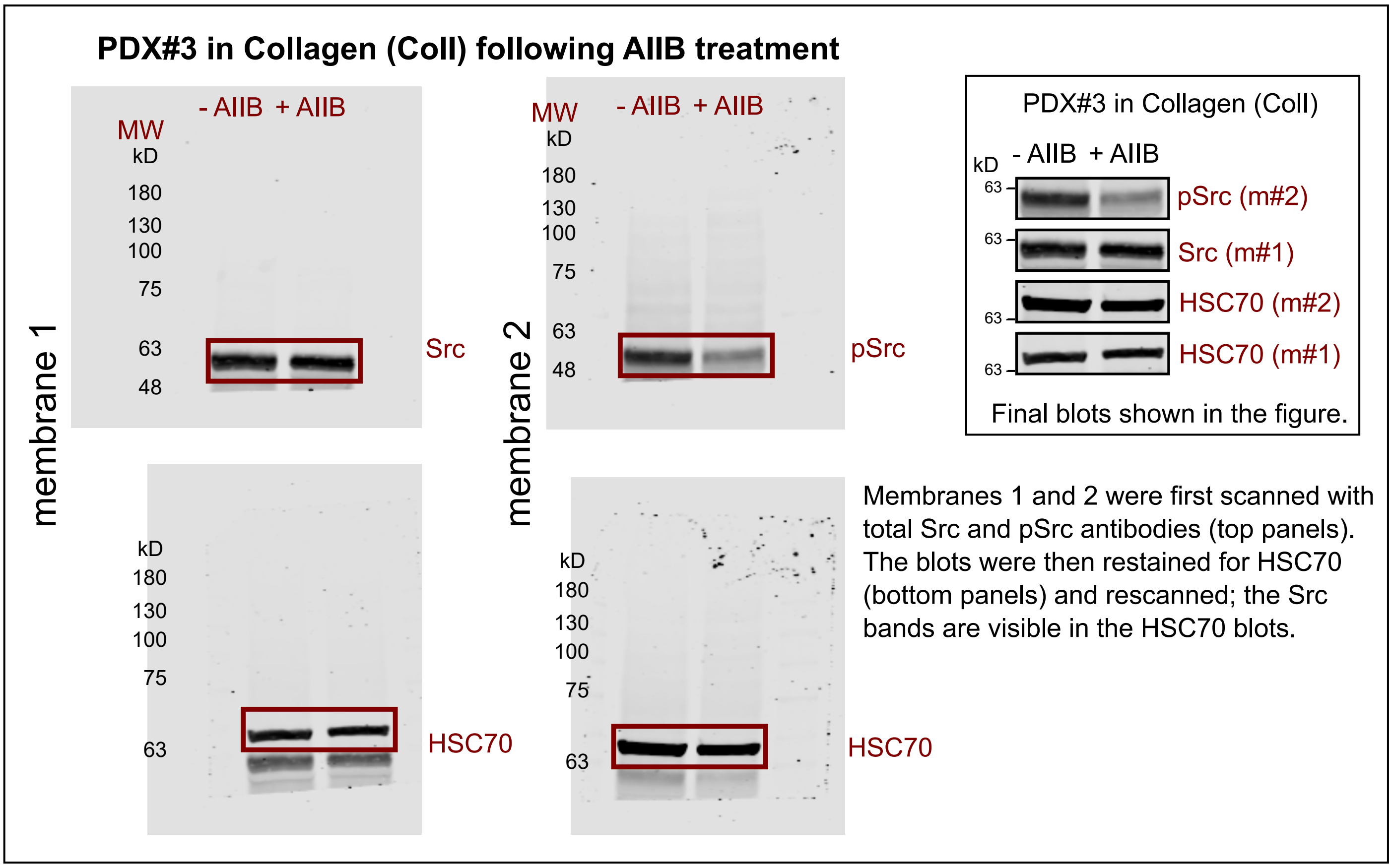

PDX#3 in Collagen (ColI) following AIIB treatment
membrane 1
MW
kD
- AIIB + AIIB
180
130
100
75
63
48
Src
kD
180
130
100
75
63
HSC70
membrane 2
MW
kD
- AIIB + AIIB
180
130
100
75
63
48
pSrc
kD
180
130
100
75
63
HSC70
PDX#3 in Collagen (ColI)
kD
- AIIB + AIIB
63
pSrc (m#2)
63
Src (m#1)
63
HSC70 (m#2)
63
HSC70 (m#1)
Final blots shown in the figure.
Membranes 1 and 2 were first scanned with total Src and pSrc antibodies (top panels). The blots were then restained for HSC70 (bottom panels) and rescanned; the Src bands are visible in the HSC70 blots.

Figure 3 C

RCP levels in PDX models: susp. vs coll

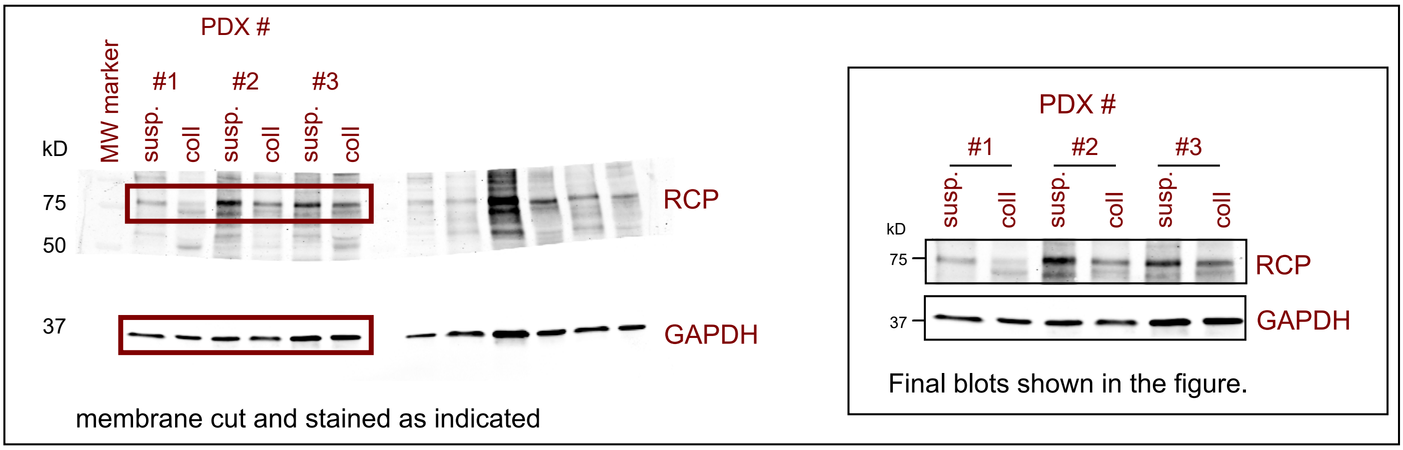

Figure 3 E

SorLA levels in PDX models: susp. vs coll

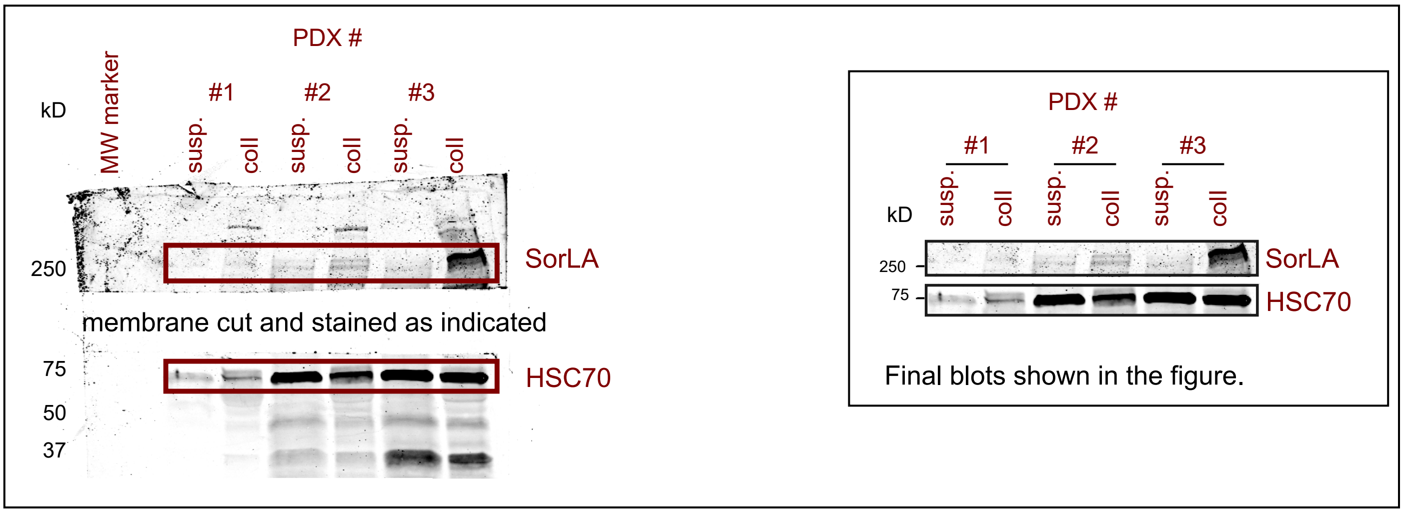

Figure 4 A

β1-integrin maturation levels in PDX models: susp. vs coll

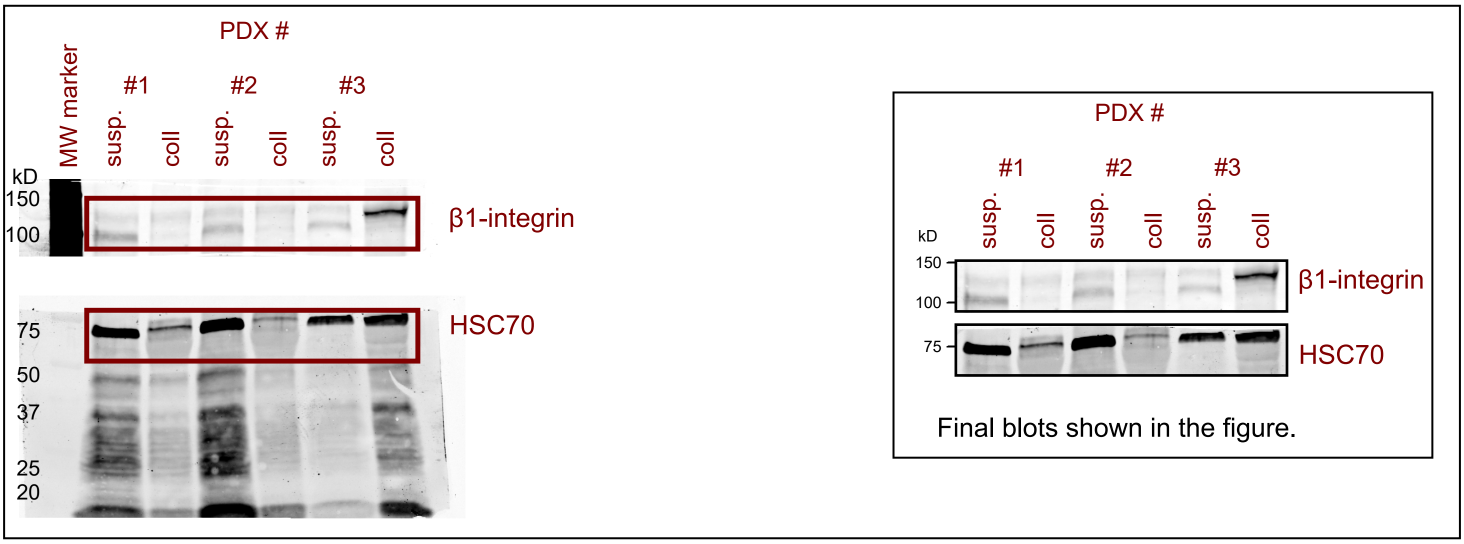

Supplement: Supplementary file 1 [file 41467_2026_75127_MOESM7_ESM.zip › statistical source data/Unprocessed_blots_Main figures.pdf]
