# Supplementary material for: Signaling downstream of tumor–stroma interaction regulates mucinous colorectal adenocarcinoma apicobasal polarity
Source: Nat Commun. 2026 Jul 4;17:8303. doi: 10.1038/s41467-026-75127-0 (PMC13469138; doi:10.1038/s41467-026-75127-0)

Supplementary information

Signaling downstream of tumor-stroma interaction regulates mucinous colorectal adenocarcinoma apicobasal polarity

Nicolas Pasquier[1,2#], Meri Pelkonen[1,#], Elise Carraz-Billat[2,], Aleksi Isomursu[1**], Raphaël Merand[2,§], Hellyeh Hamidi[1], Jacques R.R. Mathieu[2], Jouni Härkönen[1], Gautier Follain[1], Christophe Desterke[3], Zoé Fusilier[4,5], Junel Solis[6], Irina Belaya[6], Pasi Kankaanpää[6], Valeria Barresi[7], Mohamed-Amine Bani[8,9], Johanna Protin[2], Jérôme Cartry[2], Sabrina Bedja[2], Olav M. Andersen[10], Klaus Elenius[1,11,12,13], Florent Peglion[2]*, Fanny Jaulin[2] & Johanna Ivaska*[1,13,14,15,16]

[1]Turku Bioscience Centre, University of Turku and Åbo Akademi University, Turku, Finland
[2]Université Paris-Saclay, Gustave Roussy, Inserm, UMR 1279 Tumor Cell Dynamics, F-94805, Villejuif, France
[3]INSERM UMR-S1310, Université Paris-Saclay, Hôpital Paul Brousse, Villejuif F-94805, France
[4]INSERM-U932, Immunity and Cancer, Institut Curie, Paris-Cité University, Paris, France
[5]INSERM-U932, Immunity and Cancer, Institut Curie, PSL University, Paris, France
[6]Turku Bioimaging, Åbo Akademi University and University of Turku, Turku, Finland
[7]Department of Diagnostics and Public Health, University of Verona, Verona 37134, Italy
[8]Medical Biology and Pathology Department, Pathology Laboratory, Gustave Roussy Cancer Campus, Villejuif, France
[9]Platform for Experimental and Translational Pathology (PETRA), Gustave Roussy Cancer Campus, Villejuif, France
[10]Department of Biomedicine, Aarhus University, Denmark
[11]Institute of Biomedicine, and MediCity Research Laboratory, University of Turku, Turku, Finland
[12]Department of Oncology, Turku University Hospital, Turku, Finland
[13]InFLAMES Research Flagship Center, University of Turku, Turku, Finland
[14]Department of Life Technologies, University of Turku, Turku, Finland
[15]Foundation for the Finnish Cancer Institute, Helsinki, Finland
[16]Western Finnish Cancer Center, University of Turku, Turku FI-20520, Finland
# Equal contribution

*Correspondence: johanna.ivaska@utu.fi and florent.peglion@gustaveroussy.fr
**Current address: Cell Polarity, Migration and Cancer Unit, Institut Pasteur, CNRS UMR3691, Université Paris Cité, Équipe Labellisée Ligue Contre le Cancer, F-75015, Paris, France

The supplementary information file contains the following:

- Supplementary Figures 1-8 and associated legends
- Supplementary Tables 1 and 2
- Uncropped scans of all blots and gels presented in Supplementary Figures

•

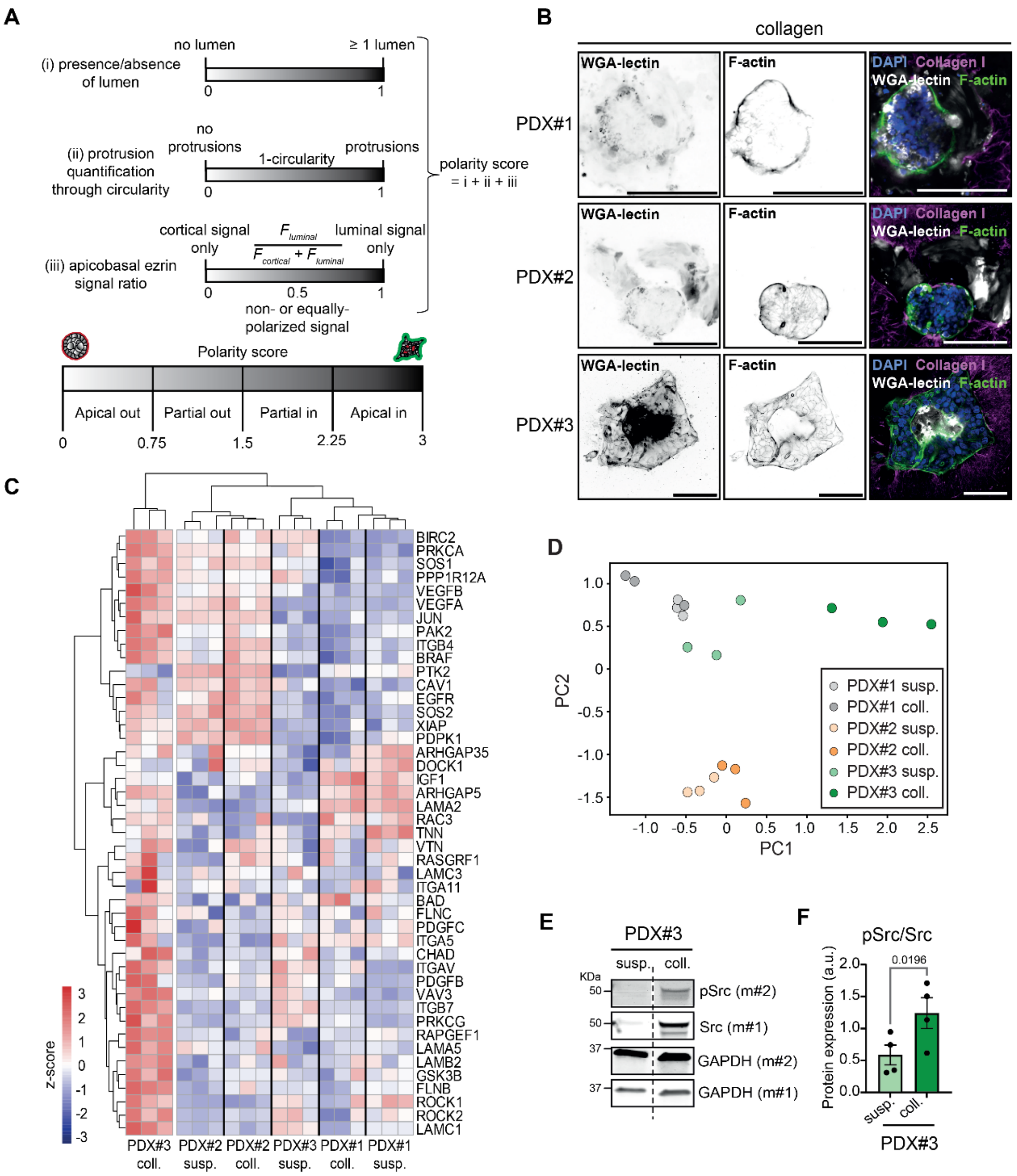

FIGS1. Characterization of apicobasal polarity of mucinous CRC PDX models

(A) Outline of polarity score calculation used for all experiments. The score (from 0: apical-out to 3: apical-in) is a sum of three parameters, i) the presence of a lumen, ii) circularity, and iii) ratio of luminal/total Ezrin fluorescence. (B) Representative immunofluorescence images of PDX#1-, PDX#2- and PDX#3-generated tumoroids in collagen, with labeled F-actin and WGA-lectin to detect mucin secretion [(n= 2 (PDX#1); 2 (PDX#2) from one biological replicate; 6 (PDX#3) from two biological replicates)]. (C) Heatmap of the 45 top differentially regulated genes in the KEGG Focal Adhesion Pathway in PDX#3 (collagen vs. suspension). (D) Associated Principal Component Analysis on the same geneset for PDX#1, PDX#2 and PDX#3 models in suspension and collagen. (E, F) Western blot

analysis of pSrc and Src levels in PDX#3 tumoroids in suspension or collagen and associated quantifications. Dashed lines indicate that non-adjacent lanes from the same blot were juxtaposed (samples from the same experimental repeat); intervening lanes were removed (n=4 biological replicates; two-tailed paired t-test). Scale bars, 100 µm; a.u.= arbitrary units.

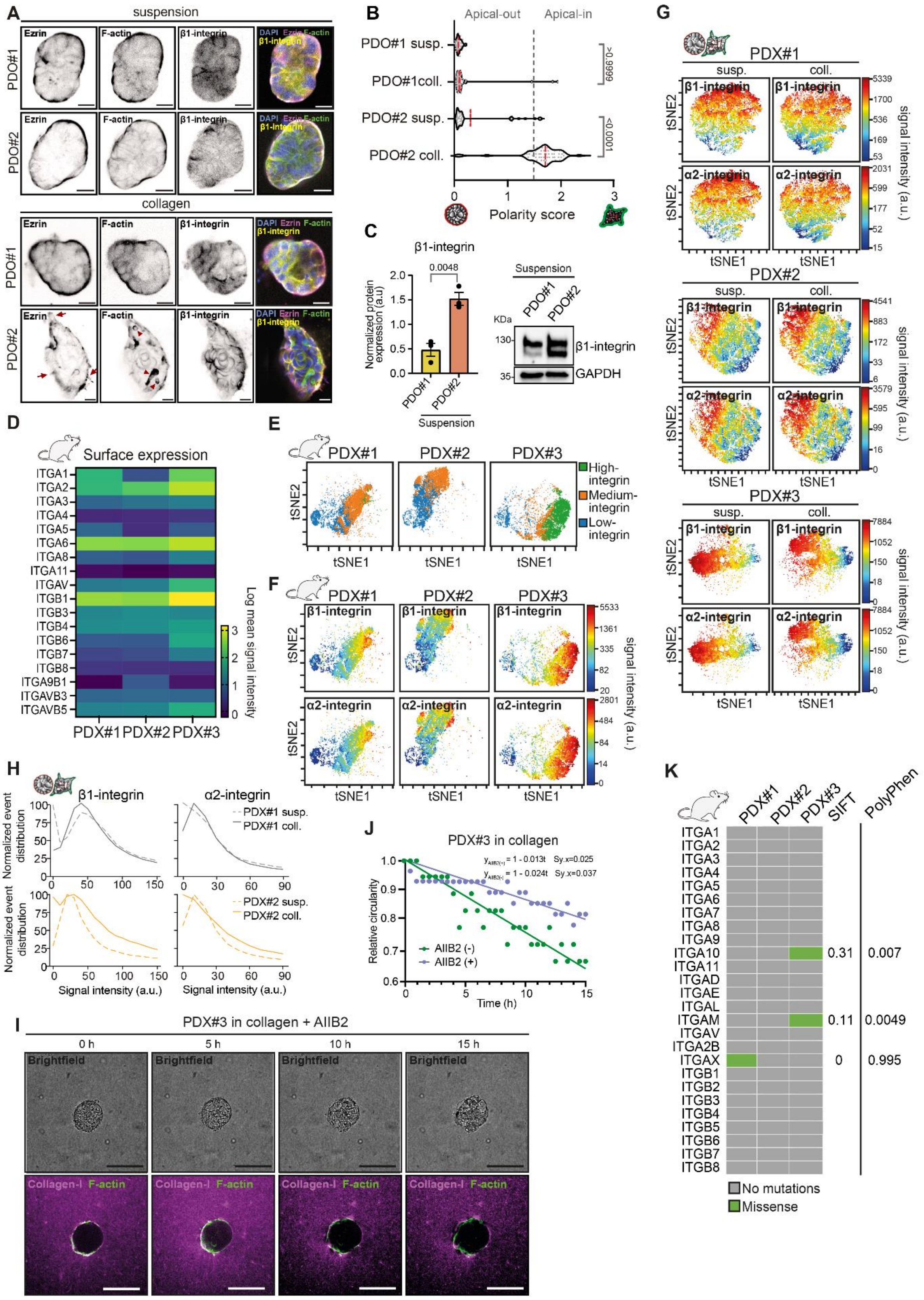

FIGS2. Organoid and tumoroid polarity in collagen correlate with integrin expression

(A) Representative immunofluorescence images of PDO#1- and PDO#2-generated spheres in suspension and in collagen, stained for Ezrin, F-actin, β1-integrin and DAPI. In the PDO#2 collagen condition, arrows indicate protrusions and arrow heads indicate lumens. Scale bars 20 µm. (B) Polarity score of PDO#1- and PDO#2-generated spheres before and after inclusion in collagen for 3 days. Data are displayed as violin plots with mean indicated by a red line. Dashed line (at polarity score 1.5) indicates the mid-point between apical-in and apical-out characteristics. Two-tailed Mann-Whitney test; PDO#1: n=55 (suspension); 59 (collagen) from four biological replicates, and for PDO#2: n=51 (suspension); 53 (collagen) from three biological replicates. (C) Western blot analysis and corresponding blot of β1-integrin in PDO#1- and PDO#2-generated spheres in suspension after 24h (n=3 biological replicates; unpaired t-test with Welch's correction). (D) A heatmap representing logarithmic mean cell-surface expression of the indicated integrins in PDX#1, PDX#2 and PDX#3 tumors dissected from mice. (E) FlowSOM clustering following a tSNE analysis of single-cell mass cytometry data obtained from PDX#1, PDX#2 and PDX#3 tumors out of mice show three distinct cell clusters based on integrin surface expression levels. (F) tSNE analysis of single-cell mass cytometry data obtained from dissected PDX#1, PDX#2 and PDX#3 tumors based on the whole panel of surface receptors (see Material and Methods, table 2), overlayed with α2-integrin and β1-integrin signal intensities. (G) tSNE analysis of PDX#1-, PDX#2- and PDX#3-generated tumoroids in suspension and collagen based on the whole panel of surface receptors, overlayed with α2-integrin and β1-integrin signal intensities. (H) Distribution of β1-integrin and α2-integrin surface expression signal intensity in PDX#1- and PDX#2-generated tumoroids in suspension and in collagen (cells > 7000). (I) Snapshots from live imaging of PDX#3 polarity reversion over 15 hours, with AIIB2 (1 µg/ml), starting from timepoint 0 (collagen embedding) (Controls shown in Fig. 1C). Scale bars 100 µm. (J) Quantification of sphere circularity from **Supplementary Movies 1 and 2**, showing different evolutions when the spheres are cultured ± AIIB2. (K) Whole-Exome Sequencing data showing mutation profiles in PDX#1, PDX#2 and PDX#3 for the different integrin subunits. a.u.= arbitrary units.

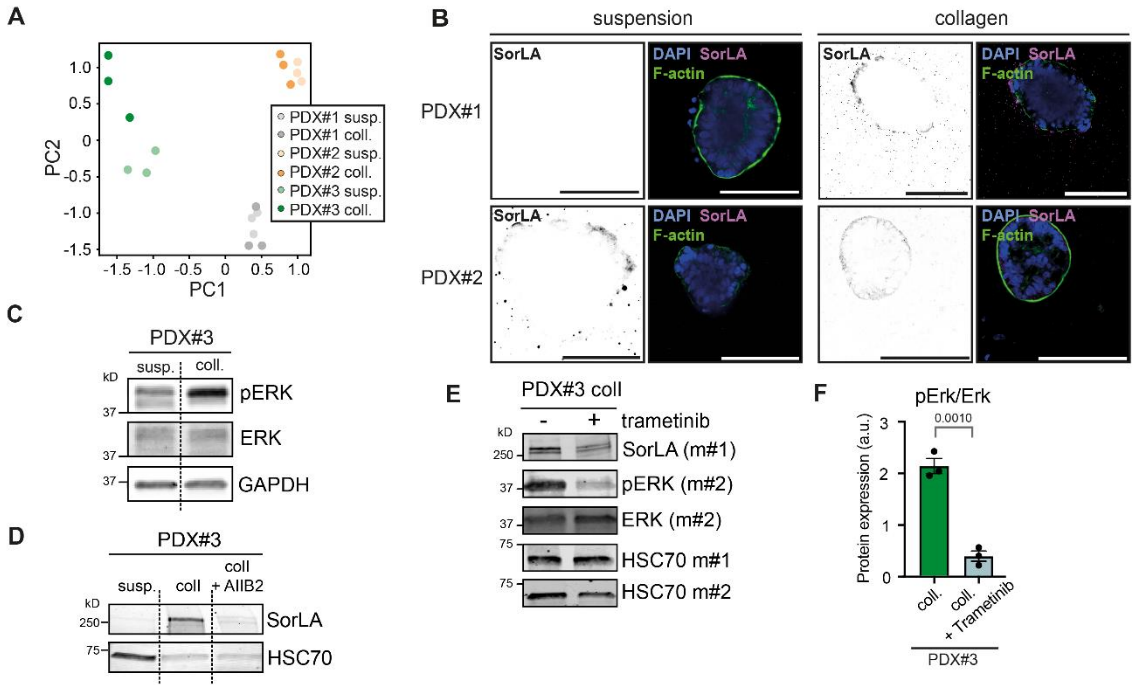

FIGS3. SorLA signaling regulates polarity in MUC CRCs.

(A) PCA associated to the relative expression of key regulators in β1-integrin trafficking, generated from an RNA microarray analysis of PDX#1-, PDX#2- and PDX#3-generated tumoroids in suspension and in collagen. (B) Immunofluorescence of PDX#1- and PDX#2-generated tumoroids in suspension and in collagen, showing SorLA staining (n= two biological replicates). (C) Western blot of pERK, ERK and GAPDH as a loading control in PDX#3-generated tumoroids in suspension and collagen. (D) Western blot of SorLA and HSC70 as loading control in PDX#3-generated tumoroids in suspension, collagen, and collagen + AIIB2 (1 µg/ml). N.B. The HSC70 loading control is the same as in FIG S5D as the samples are from the same experiment. (E, F) Representative western blot of SorLA, β1-integrin, ERK and HSC70 (loading control) and quantification of ERK phosphorylation (pERK/total ERK ratio) (F) in PDX#3 in collagen ± trametinib (250 nM) (n=3 biological replicates; two-tailed unpaired t-test with Welch's correction). Scale bars, 100 µm. Dashed lines on western blots (C, D) indicate that non-adjacent lanes from the same blot were juxtaposed (samples from the same experimental repeat); intervening lanes were removed. a.u.= arbitrary units.

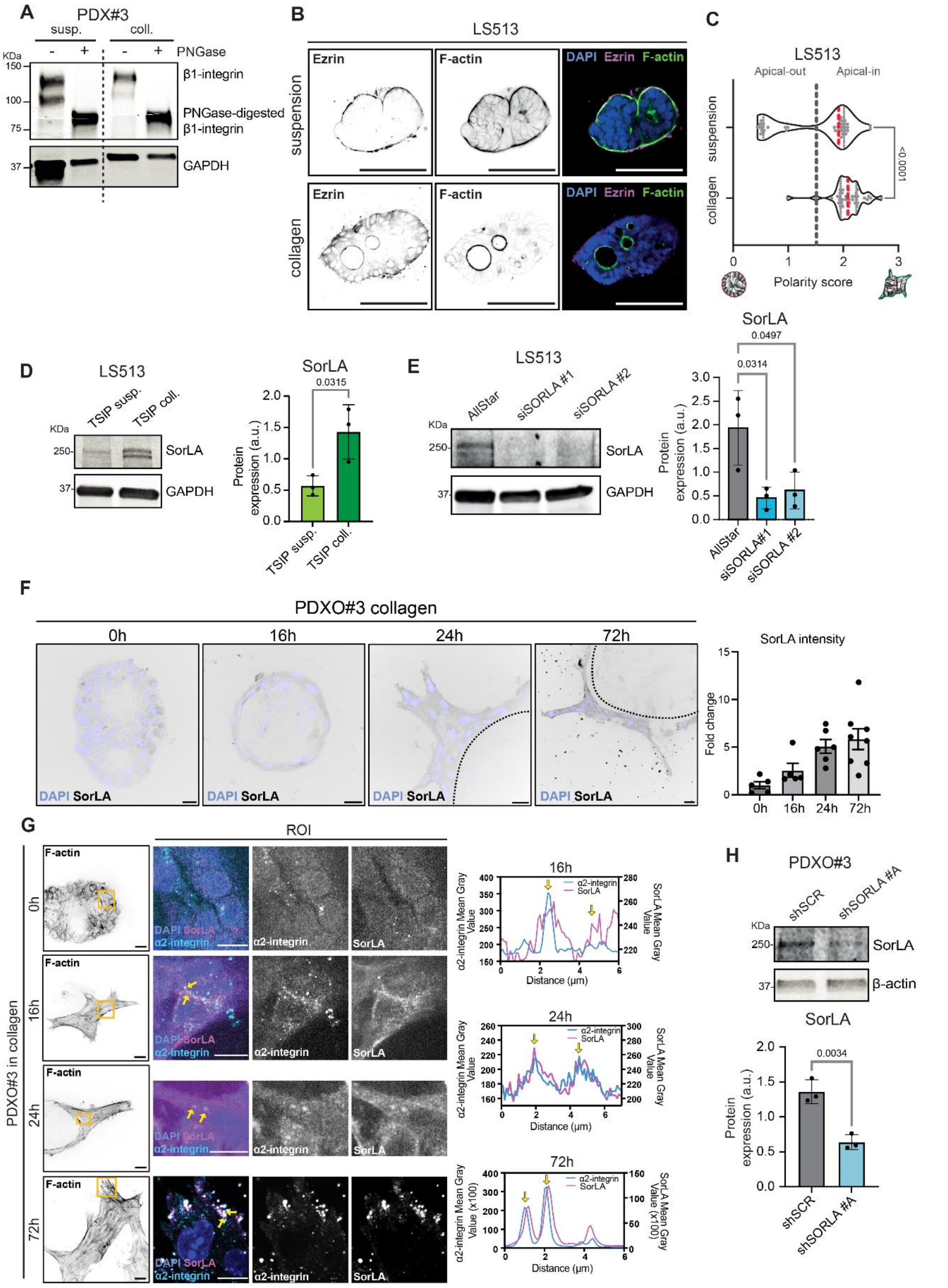

FIGS4. SorLA-dependent β1-integrin recycling loop in PDX#3.

(A) Western blot of β1-integrin in PDX#3-generated tumoroids in suspension and collagen, before and after PNGase digestion of the lysates. Dashed line on the western blot indicates that non-adjacent lanes from the same blot were juxtaposed (samples from the same experimental repeat); intervening lanes were removed. (B) Immunofluorescence of LS513 TSIPs in suspension and in collagen, showing their

polarity status through Ezrin staining. Scale bars 100 µm. (C) Comparison of polarity scores amongst models in collagen. Data are displayed as violin plots with mean indicated by a red line. Dashed line (at polarity score 1.5) indicates the mid-point between apical-in and apical-out characteristics ($n_{suspension}$=42, $n_{collagen}$=40, two-tailed Mann-Whitney test). (D) Western blot of SorLA in LS513 TSIPs in suspension and collagen (n=3 biological replicates, two-tailed unpaired t-test). (E) Western blot of SorLA in LS513 transfected with SORLA-targeting siRNA (n=3 biological replicates, ANOVA). (F) Representative immunofluorescence images of PDX#3-generated organoids (PDXO#3) fixed in suspension (0 h in collagen) or embedded in collagen (16-72 h) and stained for SorLA and DAPI [n=5 (0h); 5 (16h); 8 (24h); 8 organoids (72h) from one biological replicate]. Lumens in 24 h and 72 h PDXO#3 are indicated with a dashed line. Scale bars 20 µm. All images were acquired using identical microscope setting, and the same brightness and contrast parameters were applied to allow visualization of the relative fluorescence signals across the collagen-embedding time course in comparison to the average of 0 h. (G) Left: Representative immunofluorescence images of PDXO#3 fixed in suspension (0 h in collagen) or embedded in collagen over time (16-72h) and stained for SorLA, α2-integrin, F-actin and DAPI. F-actin maximum intensity projections show the sphere morphology and protrusions. Yellow boxes indicate the zoomed-in insets (ROI) and yellow arrows highlight overlapping SorLA and α2-integrin signals plotted in the graphs on the right. Scale bar 20 µm (main),10 µm (insets). All images were acquired using identical microscope settings. Since signal intensities varied between early and late time points, brightness and contrast were adjusted individually for each image to visualize SorLA and α2-integrin staining patterns. (H) Western blot of SorLA in PDXO#3 infected with SORLA-targeting shRNA (n=3 biological replicates, two-tailed unpaired t-test). a.u.= arbitrary units.

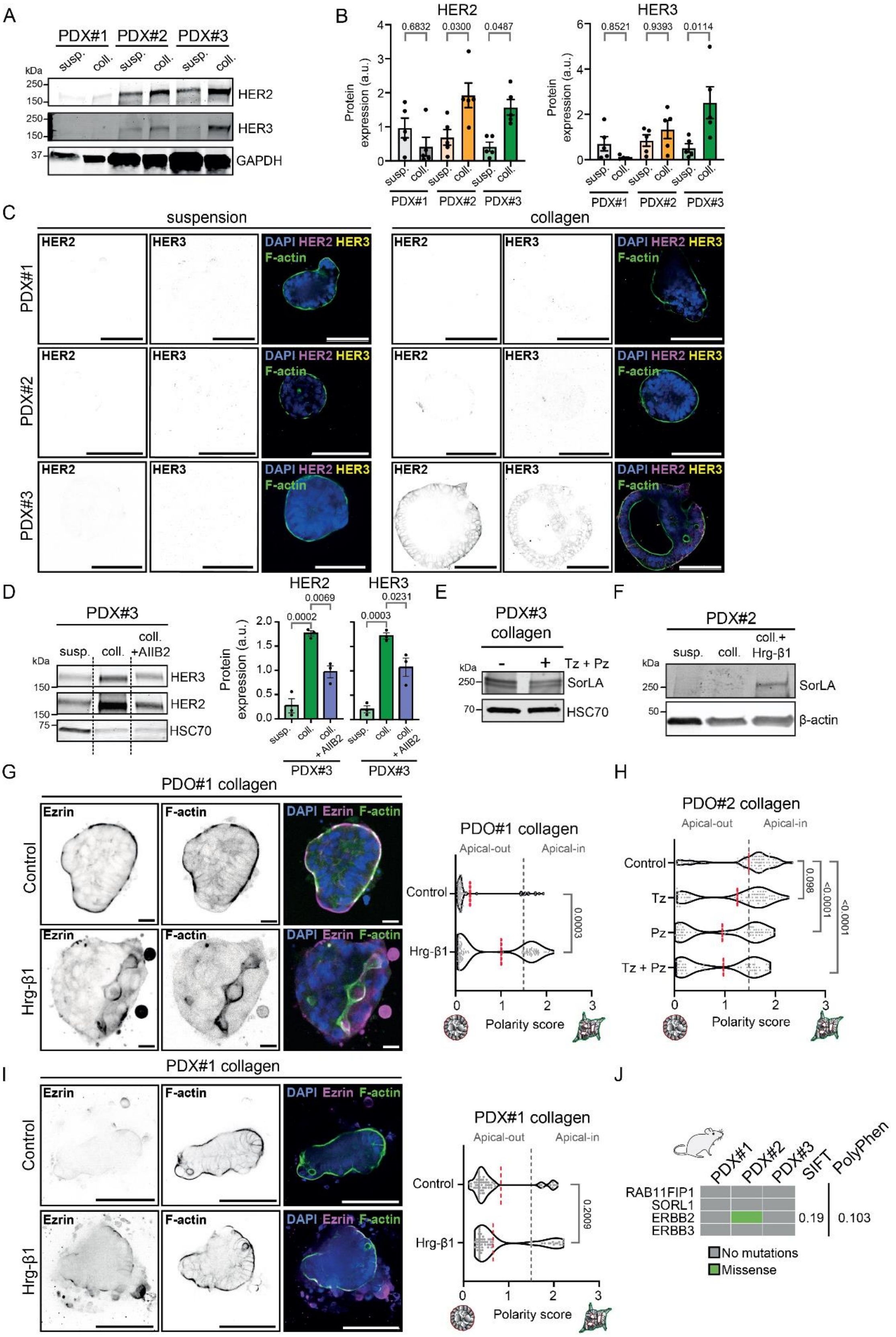
A
PDX#1 PDX#2 PDX#3
susp. coll. susp. coll. susp. coll.
kDa
250
150
HER2
HER3
37
GAPDH
B
HER2
HER3
Protein expression (a.u.)
0.6832 0.0300 0.0487
0.8521 0.9393 0.0114
PDX#1 PDX#2 PDX#3
C
suspension
collagen
PDX#1
PDX#2
PDX#3
HER2
HER3
DAPI HER2 HER3 F-actin
D
PDX#3
susp. coll. coll. +AIIB2
HER3
HER2
HSC70
0.0002 0.0069
0.0003 0.0231
E
PDX#3 collagen
Tz + Pz
SorLA
HSC70
F
PDX#2
susp. coll. coll.+ Hrg-β1
SorLA
β-actin
G
PDO#1 collagen
Control
Hrg-β1
Ezrin
F-actin
DAPI Ezrin F-actin
Apical-out Apical-in
0.0003
Polarity score
H
PDO#2 collagen
Control
Tz
Pz
Tz + Pz
0.098
<0.0001
<0.0001
I
PDX#1 collagen
0.2009
J
PDX#1 PDX#2 PDX#3 SIFT PolyPhen
RAB11FIP1
SORL1
ERBB2
ERBB3
0.19
0.103
No mutations
Missense

FIGS5. HER2 and HER3 characterization in MUC CRC PDXs.

(A) Western blot of HER2, HER3 and GAPDH (loading control) in PDX#1-, PDX#2- and PDX#3-generated tumoroids in suspension and in collagen. (B) Corresponding quantifications of HER2 and HER3 in PDX#1-, PDX#2- and PDX#3-generated tumoroids in suspension and in collagen (mean ± s.e.m; n=5 biological replicates; One-way ANOVA with Tukey's multiple comparison test). HER2 and HER3 have been blotted on the same membrane and were therefore normalized to the same GAPDH signal. (C) Immunofluorescence of PDX#1/2/3-generated tumoroids in suspension and in collagen, showing HER2 and HER3 [(n= 10 (PDX#1 suspension); 10 (PDX#1 collagen); 10 (PDX#2 suspension); 7 (PDX#2 collagen); 4 (PDX#3 suspension); 10 (PDX#3 collagen) tumoroids from one biological replicate)]. (D) Western blot quantifications and corresponding blot of HER2 and HER3 expression in PDX#3 in suspension and collagen ± AIIB2 (1µg/ml) (mean ± s.e.m; n=3 biological replicates, One-way ANOVA with Tukey's multiple comparison test). N.B. The HSC70 loading control is the same as in FIG S3D as the samples are from the same experiment. Dashed lines on western blot indicate that non-adjacent lanes from the same blot were juxtaposed (samples from the same experimental repeat); intervening lanes were removed. (E) Western blot showing SorLA and HSC70 (loading control) in PDX#3 in collagen ± a combination of trastuzumab and pertuzumab (Tz + Pz; both 10 µg/ml). (F) Western blot showing SorLA expression in PDX#2-generated tumoroids in suspension, collagen and collagen + Hrg-β1. (G) Immunofluorescence (Ezrin staining) and polarity score of PDO#1-generated spheres in collagen ± Hrg-β1 treatment (20 ng/ml). Scale bar 20 µm. For polarity score data are displayed as violin plots with mean indicated by a red line. Dashed line (at polarity score 1.5) indicates the mid-point between apical-in and apical-out characteristics (n_{Ctrl}=85 and $n_{Hrg\text{-}\beta1}$=121 from four biological replicates; two-tailed Mann Whitney test). (H) Comparison of polarity scores of PDO#2-generated spheres in collagen treated with HER2-blocking antibody (Tz), HER3-blocking antibody (Pz) or combination of both (Tz+Pz). Data are displayed as violin plots with mean indicated by a red line. Dashed line (at polarity score 1.5) indicates the mid-point between apical-in and apical-out characteristics ($n_{control}$=67, n_{Tz}=63, n_{Pz}=69, n_{Tz+Pz}=49 from three biological replicates; Kruskal-Willis test with Dunn's multiple comparison tests). (I) Immunofluorescence (Ezrin staining) and polarity score of PDX#1-generated tumoroids in collagen ± Hrg-β1 (20 ng/ml). Scale bar 100 µm. For polarity score data are displayed as violin plots with mean indicated by a red line. Dashed line (at polarity score 1.5) indicates the mid-point between apical-in and apical-out characteristics (n_{Ctrl}=59, $n_{Hrg\text{-}\beta1}$ =55, two-tailed Mann-Whitney test). (J) Whole-Exome Sequencing data, showing mutation profiles in PDX#1, PDX#2 and PDX#3 in *RAB11FIP1*, *SORL1*, *ERBB2* and *ERBB3*. a.u.= arbitrary units.

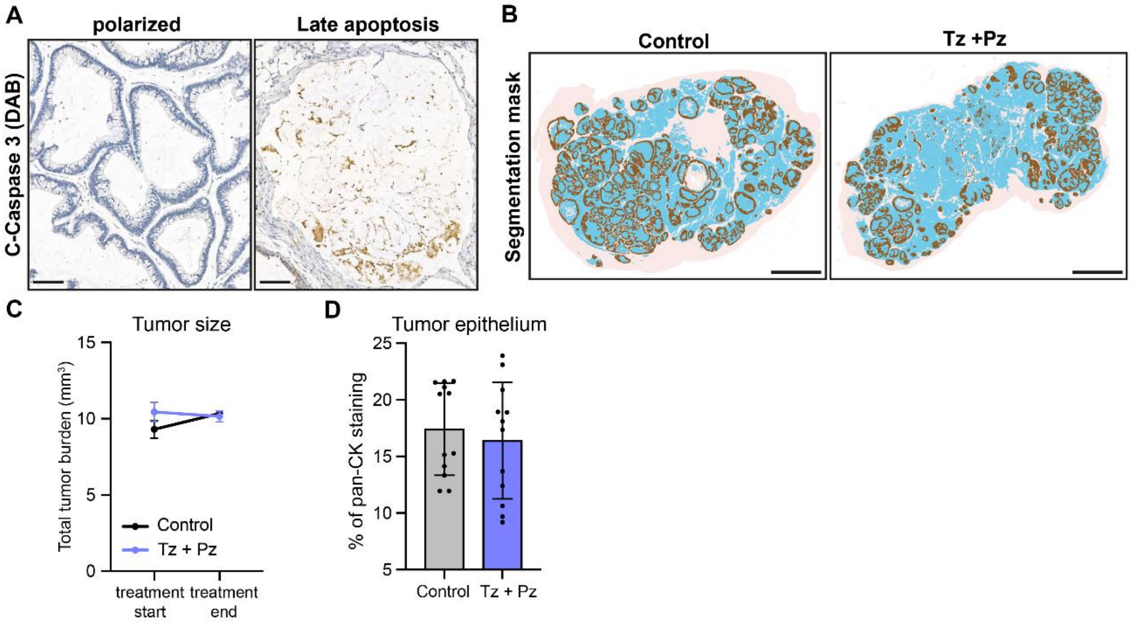

FIGS6. HER2/HER3 regulate PDX polarity in vivo.

(A) Representative images of tumor epithelial structures characterized as polarized (left) and dead (right), stained with cleaved caspase-3 antibody to show late apoptotic traces in the “dead” class. Scale bar 100 µm. (B) Segmentation mask obtained from the representative images shown in Fig.5G. Scale bar 1.5 mm. (C) Total tumor burden in control and trastuzumab and pertuzumab combination (Tz + Pz) treated mice. Average of total tumor size from both flanks is plotted (n=2 mice/group). (D) Quantification of the percentage of tumor epithelial structures (Pan-Cytokeratin mask) over the total size of the sample cut, comparing control and treated tumors (mean ± SD; n=12 tumor sections out of four tumors/group; Mann-Whittney statistical test, non-significant).

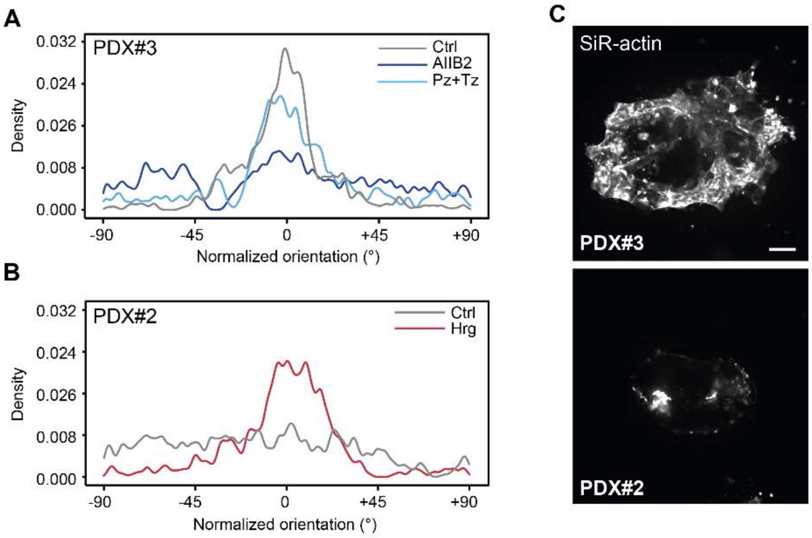

FIGS7. Local collagen alignment proximal to PDX#2 and PDX#3 tumoroids.

(A) Normalized orientation of collagen fibers proximal to PDX#3-generated tumoroids after 6 days in collagen, with AIIB2 treatment (1 µg/ml) and combination of trastuzumab and pertuzumab treatment (Tz+Pz, both at 10 µg/ml). (B) Normalized orientation of collagen fibers proximal to PDX#2-generated tumoroids after 6 days in collagen ± Hrg-β1 treatment (20 ng/ml). (C) Fluorescence images of PDX#2- and PDX#3-generated tumoroids embedded in collagen, used for masking the cells for the displacement analysis in Fig. 6C-D. Scale bar 100 µm.

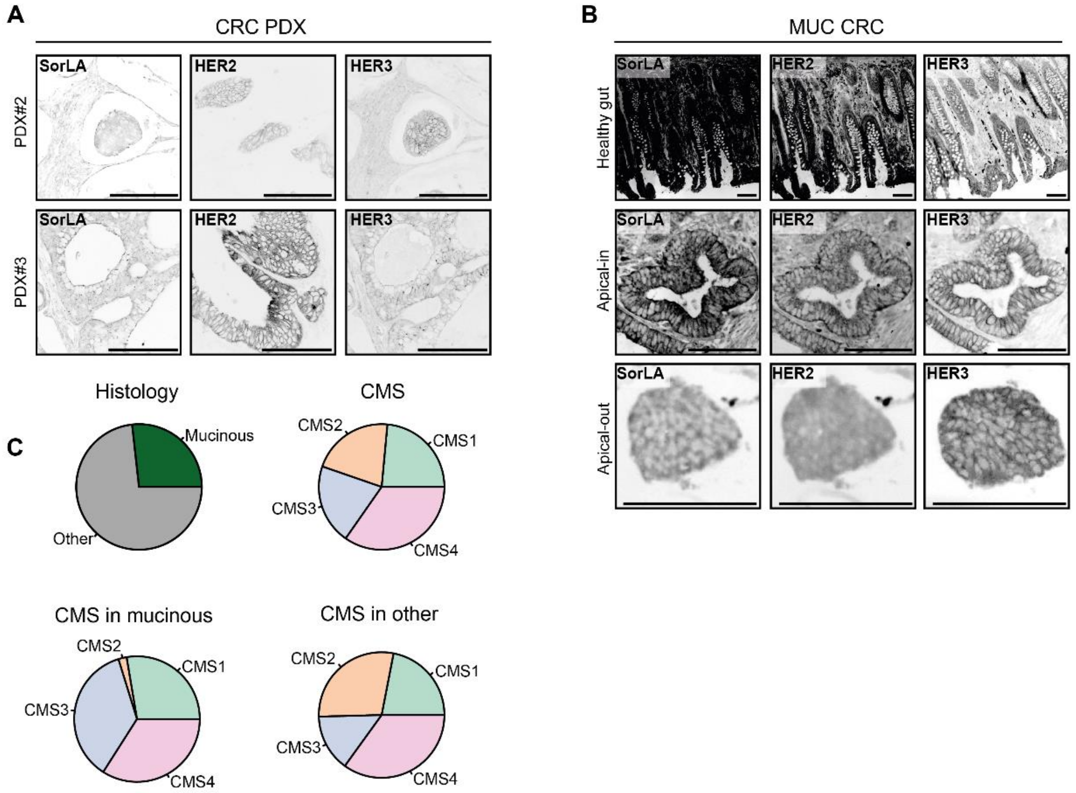

FIGS8. HER2, HER3 and SorLA expressions are correlated *in vivo.*

(A) Immunohistochemistry of PDX#2 and PDX#3 tumors stained for HER2 and SorLA, and HER2 and HER3. (B) Immunohistochemistry of healthy gut tissue, apical-in and apical-out cancer structures stained for HER2, HER3 and SorLA. Same specimens as in Fig. 7A. (C) Classification of the histotypes of the cohort used for FIG7D-E, further stratified into mucinous and non-mucinous CRCs. Scale bars, 100 μm.

Supplementary Table 1 – MUC CRC PDXs and PDOs used in the study

Models	Sample ID	Clinical Trial	Origin	Profiling
PDX#1	LRB-009C	CReMEC	Metastatic (peritoneum)	MSS, BRAF WT, $KRAS^{G12V}$
PDX#2	IGR-012P	CReMEC	Primary	MSS, BRAF WT, $KRAS^{G13D}$
PDX#3	IGR-014P	CReMEC	Primary	MSS, BRAF WT, $KRAS^{G12D}$
PDO#1	CGR0089	STING (NCT02517892)	Metastasis (liver)	MSS, BRAF WT $KRAS^{G12V}$,
PDO#2	CGR0116	Organotreat 01 (NCT05267912)	Metastasis (liver)	MSS, BRAF WT, KRAS WT

Supplementary Table 2 – Antibodies and Reagents (WB=Western Blot, IF=Immunofluorescence, IHC=Immunohistochemistry, FB=Function blocking, MC=Mass cytometry, ELISA=enzyme-linked immunosorbent assay)

Protein	Company	Catalog Number	Application	Concentration
Ezrin	DHSB	AB-210031	IF	1: 400
SorLA	C.M. Petersen (Aarhus U)		IF	1:200
SorLA (serum raised in goat)	Olav M. Andersen	Ref: PMID:16174740, 31138794	IF (PDXO#3)	1:10
RAB11FIP1 (RCP)	ThermoFischer	PA5-55276	IF/WB	1 :400 / 1 :1000
HER2	ThermoFischer	MA5-14 57	IF/WB/IHC	1 :400/1 :1000/1 :400
HER3	Cell Signaling	12708S	IF/WB	1 :1000/1 :100
HSC70	Enzo	ADI-SPA-815	WB	1 :1000
β-actin	Sigma	A1978	WB	1 :1000
P5D2 (Total β1-integrin)	Abcam	Ab193592	IF	1 :500
12G10 (Active β1-integrin)	Abcam	Ab202641	IF	1 :500
β1-integrin (EP1041Y)	Abcam	ab52971	IF	1:1000
WGA-lectin	GeneTex	GTX01502	IF	1 :500
CNA-35	In-house		IF	40 ug/ml

Phalloidin 488	Invitrogen	A12379	IF	1 :1000
Phalloidin 647	Sigma	65906	IF	1 :1000
Phalloidin 750	Sigma	07373	IF	1 :1000
SorLA	BD Biosciences	624084	WB	1 :500
Anti-ms 568	Invitrogen	A10037	IF	1 :500
Anti-ms 488	Invitrogen	A21202	IF	1 :500
Anti-Rbt 488	Invitrogen	A21206	IF	1 :500
Anti-Rbt 561	Invitrogen	A10042	IF	1 :50
β-actin	Sigma	A1978	WB	1 :1000
GAPDH	HyTest	5G4MAB6C5	WB	1 :2000
Erk	Cell Signaling	4696S	WB	1 :500
p-Erk	Cell Signaling	4370S	WB	1 :500
Src	Cell Signaling	2108S	WB	1 :500
p-Src (active)	Cell Signaling	2101S	WB	1 :500
Ms sec 650	Azure Biosystems	AC2166	WB	1 :1000
Ms sec 800	Azure Biosystems	AC2135	WB	1 :1000
Rbt sec 650	Azure Biosystems	AC2165	WB	1 :1000
Rbt sec 800	Azure Biosystems	AC2134	WB	1 :1000
HER3	Dako	DAK-H3-IC	IHC	1 :50
SorLA	Atlas	HPA031321	IHC	1 :400
Pan CK	Invitrogen	MA5-13203	IHC	1 :400
Cleaved Caspas-3	Cell Signaling	9661S	IHC	1:800
Ezrin	BD Bioscience	610603	IF	1:200
P1E6 (α2-integrin)	DSHB	AB2619597	FB	10 µg/ml
AIIB2 (β1-integrin)	DHSB	AB528306	FB	1 µg/ml
AIIB2 (β1-integrin)	In-house		ELISA	5 µg/ml
CD29	BD Bioscience	610468	eLISA	5 µg/ml
^{89}Y-Integrin-αII	AH Diagnostics	3089004B	MC	1 :100
^{141}Pr-EpCA	AH Diagnostics	3141006B	MC	1 :100

^{142}Nd-PETA-	AH Diagnostics	3142011B	MC	1 :100
^{143}Nd-N-Cadheri	AH Diagnostics	3143016B	MC	1 :100
^{145}Nd-Syndecan-	AH Diagnostics	3145003B	MC	1 :100
^{146}Nd-Integrin-β	AH Diagnostics	3145011B	MC	1 :100
^{148}Nd-HER	AH Diagnostics	3148011A	MC	1 :100
^{149}Sm-CD3	AH Diagnostics	3149013B	MC	1 :100
^{150}Nd-Integrin-αvβ	AH Diagnostics	3150026B	MC	1 :100
^{151}Eu-ICAM-	AH Diagnostics	3151015B	MC	1 :100
^{156}Gd-Integrin-β	AH Diagnostics	3156007B	MC	1 :100
^{158}Gd-E-Cadherin	AH Diagnostics	3158018B	MC	1 :100
^{159}Tb-CD9	AH Diagnostics	3159022B	MC	1 :100
^{160}Gd-Integrin-α	AH Diagnostics	3160015B	MC	1 :100
^{161}Dy-Integrin-α	AH Diagnostics	3161012B	MC	1 :100
^{162}Dy-Integrin-β	AH Diagnostics	3162026B	MC	1 :100
^{163}Dy-Integrin-α	AH Diagnostics	3163015B	MC	1 :100
^{164}Dy-Integrin-α	AH Diagnostics	3164006B	MC	1 :100
^{165}Ho-Notch	AH Diagnostics	3165026B	MC	1 :100
^{166}Er-CD4	AH Diagnostics	3166001B	MC	1 :100
^{168}Er-Integrin-α9β	AH Diagnostics	3168013B	MC	1 :100
^{169}Tm-CD2	AH Diagnostics	3169004B	MC	1 :100
^{170}Er-ICAM-	AH Diagnostics	3170014B	MC	1 :100
^{171}Yb-CD	AH Diagnostics	3171009B	MC	1 :400
^{173}Yb-Integrin-β	AH Diagnostics	3173008B	MC	1 :100
^{174}Yb-Integrin-α	AH Diagnostics	3174018B	MC	1 :100
^{176}Yb-NCA	AH Diagnostics	3176001B	MC	1 :100
^{209}Bi-CD4	AH Diagnostics	3209004B	MC	1 :100
^{11} Cd-EGFR	Biolegend	352902	MC	1 :50
^{114}Cd-Integrin-αV	R&D Systems	MAB1219	MC	1 :50
^{111}Cd-Integrin-α3	Sigma	MAB1952Z	MC	1 :50
^{166}Cd-HER4	R&D Systems	MAB11311	MC	1 :50
^{110}Cd HER3	R&D Systems	MAB3481	MC	1 :50

^{106}Cd-Integrin-α11	R&D Systems	MAB4235	MC	1 :50
^{144}Nd-Syndec n-4	R&D Systems	MAB29181	MC	1 :50
^{152}Sm-<br>Integrin αvβ5	R&D Systems	MAB2528	MC	1 :50
^{155}Gd-Integrin-α8	R&D Systems	MAB6194	MC	1 :50
^{175}Lu-Integrin-β8	R&D Systems	MAB4775	MC	1 :50
^{153}Eu-Integrin-β6	R&D Systems	MAB4155	MC	1 :50
^{147}Sm-CD166	Biolegend	343902	MC	1 :200
^{154}Sm-Notch-1	Biolegend	352102	MC	1 :50
^{167}Er-Notch-3	Biolegend	345407	MC	1 :50
^{172}Yb-Neuropilin-1	Biolegend	354502	MC	1 :50
^{11} Cd-CD10	Biolegend	312223	MC	1 :50

Figure S1 E

pSrc and Src levels in PDX#3: suspension (susp.) vs collagen (coll)

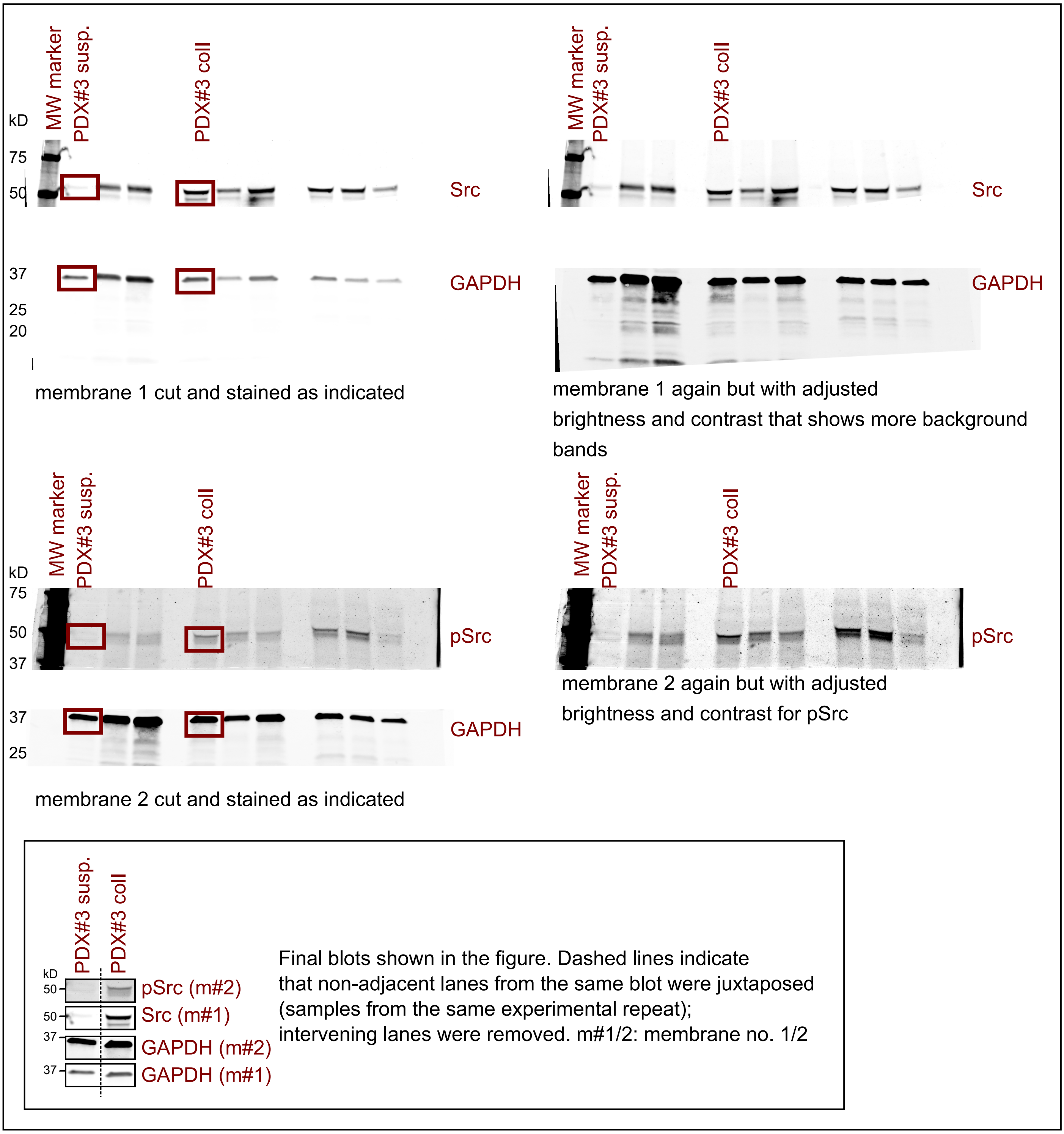

Figure S2 C

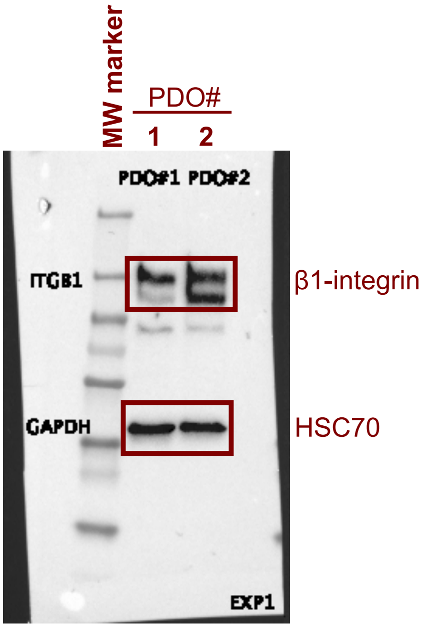

MW marker
PDO#
1
2
PDO#1
PDO#2
ITGB1
GAPDH
EXP1
β1-integrin
HSC70

Figure S3 C

pERK and ERK levels in PDX#3: suspension (susp.) vs collagen (coll)

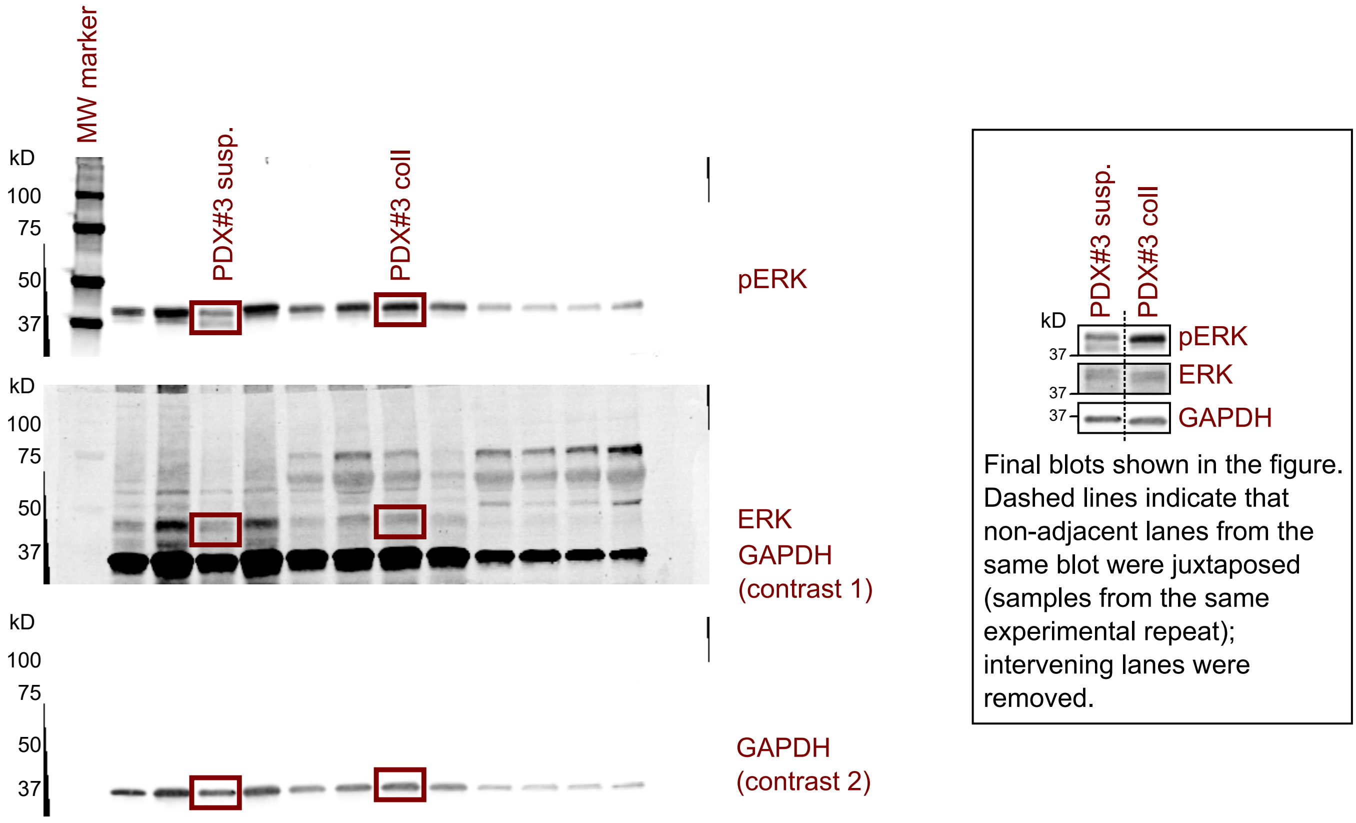

Final blots shown in the figure. Dashed lines indicate that non-adjacent lanes from the same blot were juxtaposed (samples from the same experimental repeat); intervening lanes were removed.

Figure S3 D

SorLA levels in PDX#3: suspension (susp.) vs collagen (coll) + AIIB2

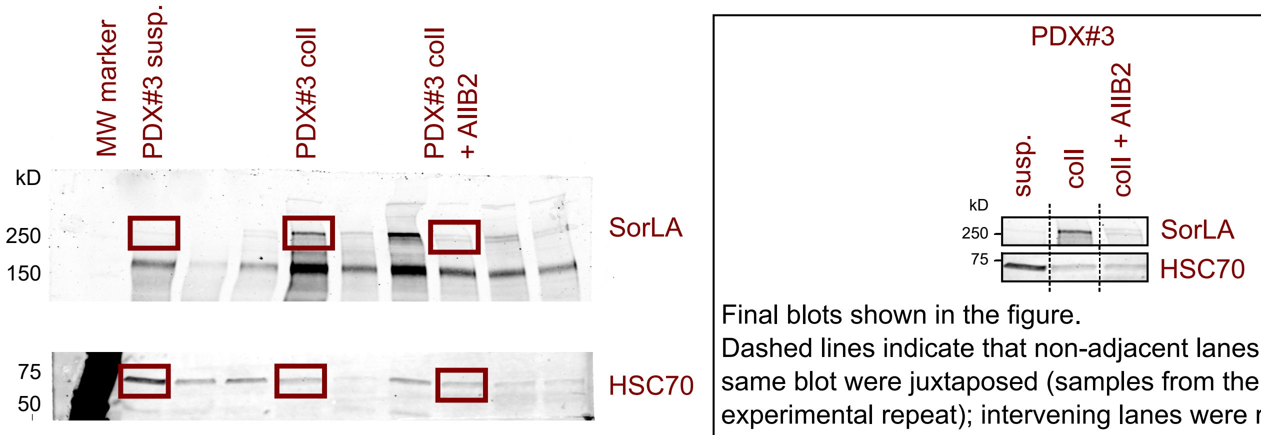

Final blots shown in the figure.
Dashed lines indicate that non-adjacent lanes from the same blot were juxtaposed (samples from the same experimental repeat); intervening lanes were removed.
N.B. this experiment also included blotting for HER2 and HER3 - blots shown in Figure S5D. Therefore the HSC70 blot is same for S3D and S5D!

Figure S3 E

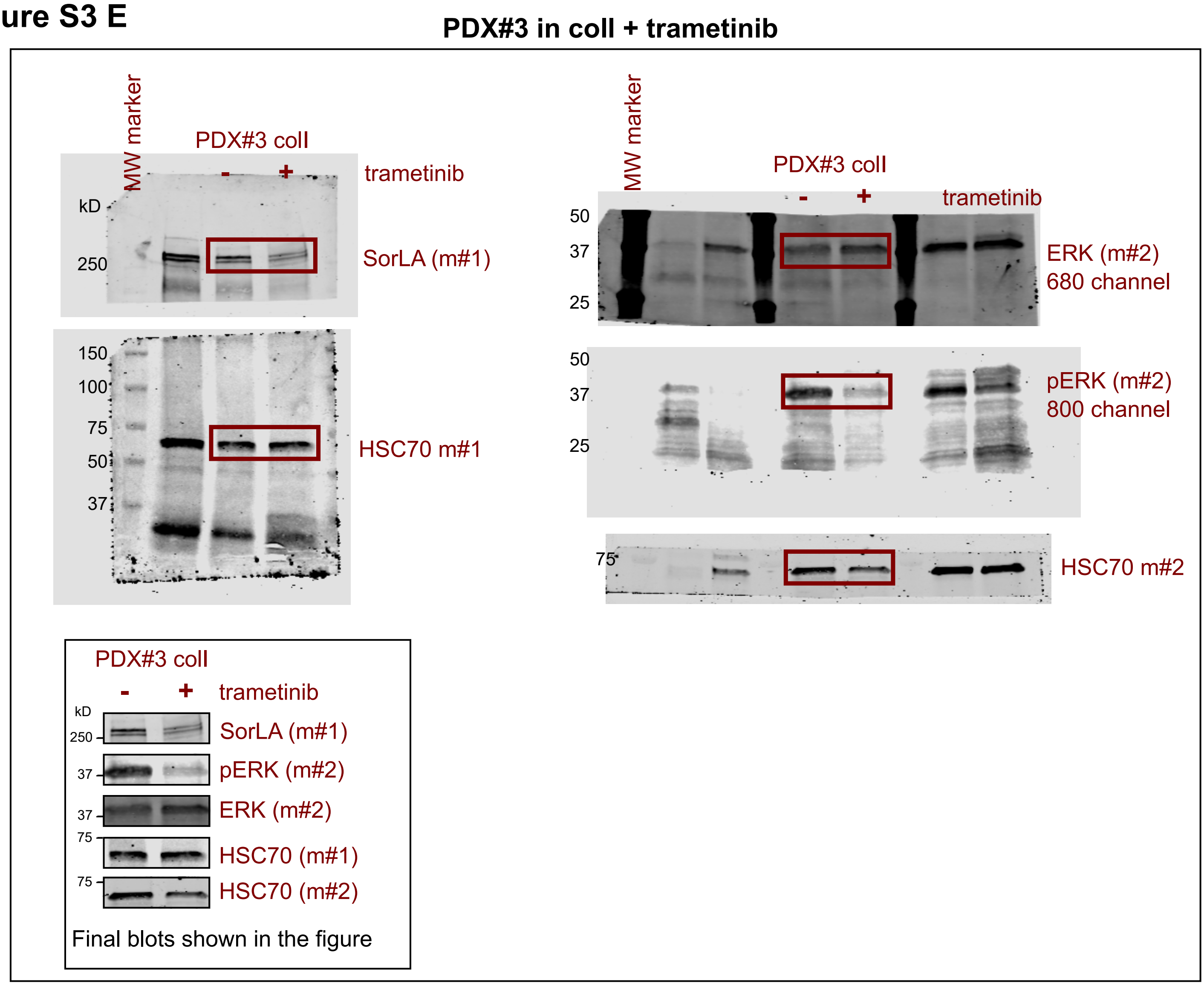
PDX#3 in coll + trametinib
MW marker
PDX#3 coll
- +
trametinib
kD
250
SorLA (m#1)
150
100
75
50
37
HSC70 m#1
MW marker
PDX#3 coll
- +
trametinib
50
37
25
ERK (m#2)
680 channel
50
37
25
pERK (m#2)
800 channel
75
HSC70 m#2
PDX#3 coll
- +
trametinib
kD
250
SorLA (m#1)
37
pERK (m#2)
37
ERK (m#2)
75
HSC70 (m#1)
75
HSC70 (m#2)
Final blots shown in the figure

Figure S4 A

PNGase digestion - β1 integrin - in PDX#3: suspension (susp.) vs collagen (coll)

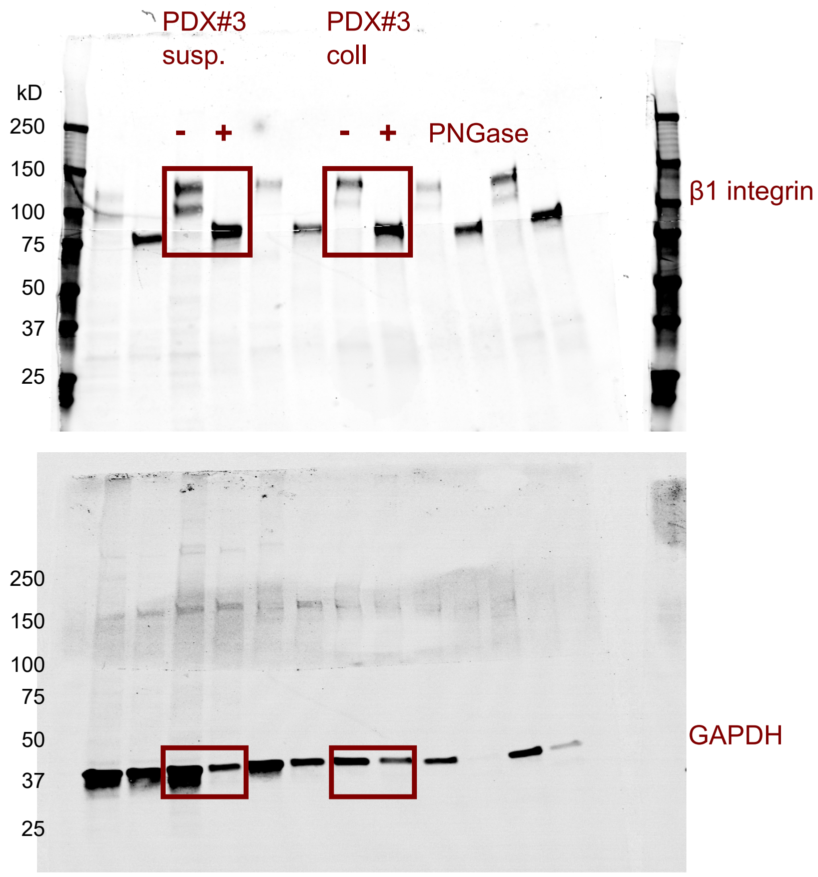

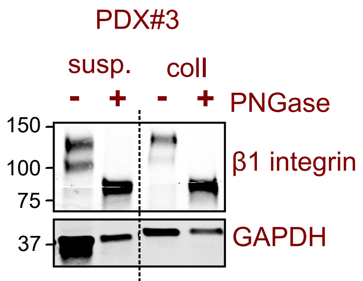

Final blots shown in the figure.
Dashed lines indicate that non-adjacent lanes from the same blot were juxtaposed (samples from the same experimental repeat); intervening lanes were removed. Please note, the membrane was cut and this cut is visible on the blot.

Figure S4 D

SorLA levels - LS513 TSIPs susp. vs coll

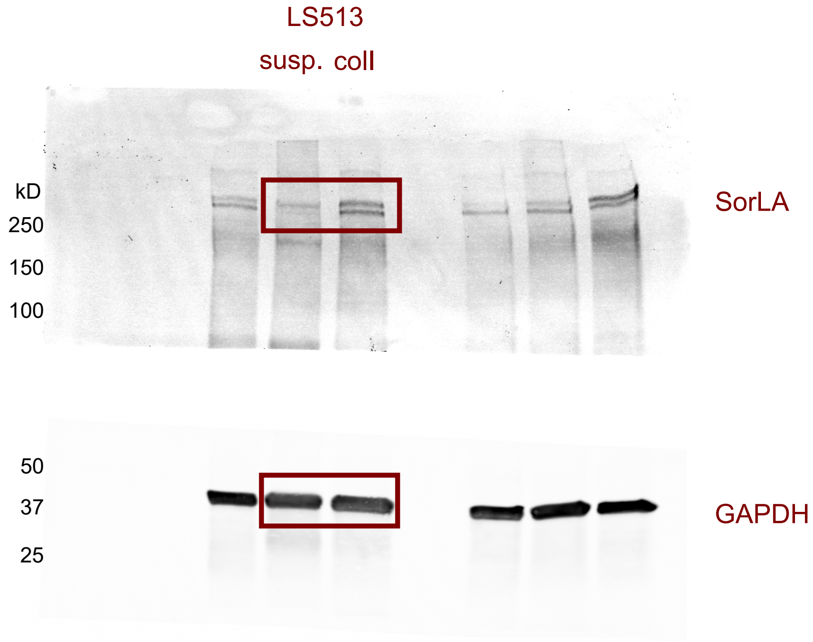

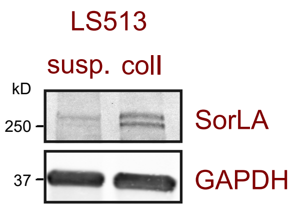

Final blots shown in the figure.

Figure S4 E

LS513 - SorLA knockdown (siRNA)

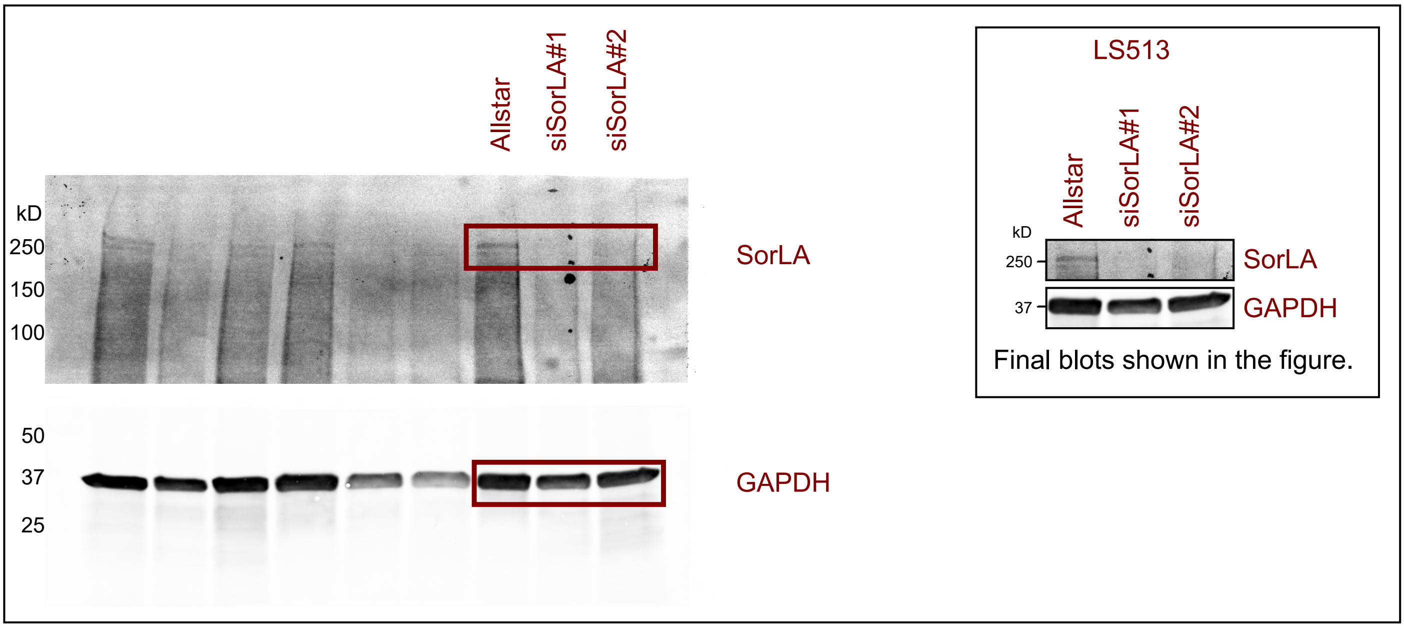

Figure S4 F

LS513 - SorLA knockdown (shRNA)

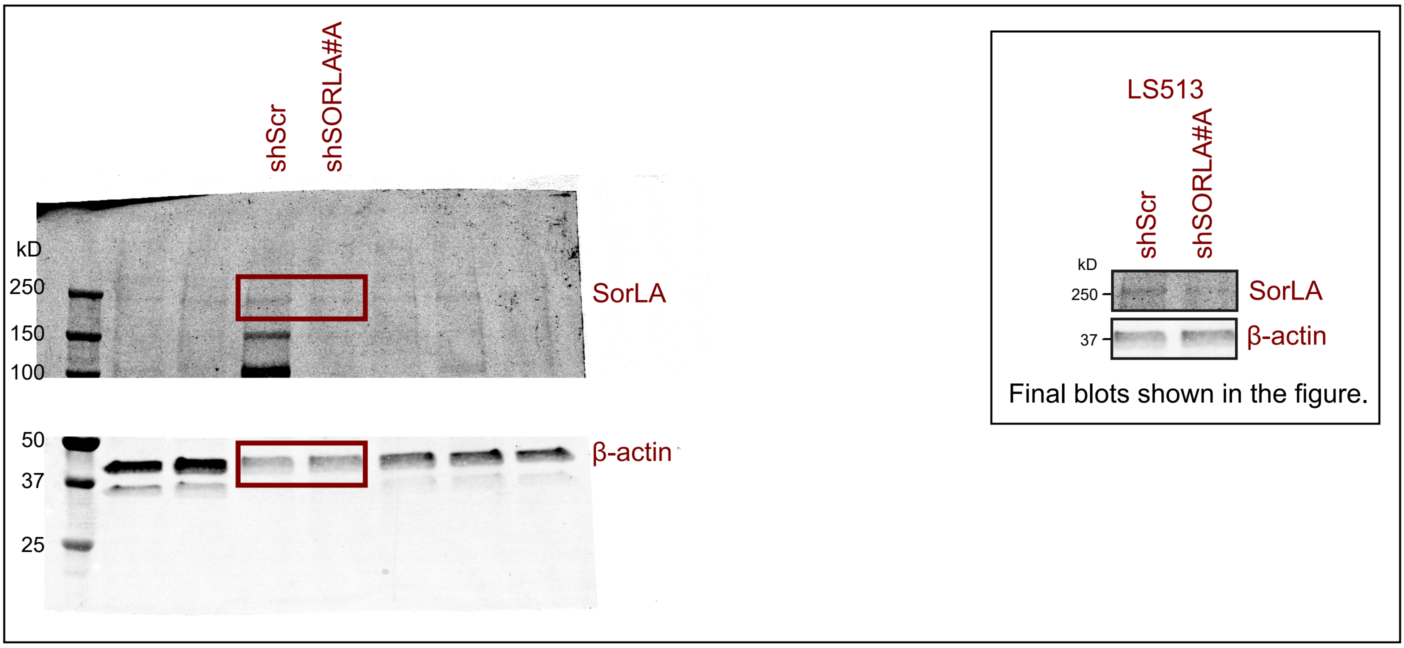

Figure S5 A

HER2 and HER3 levels - PDX models, susp. vs coll

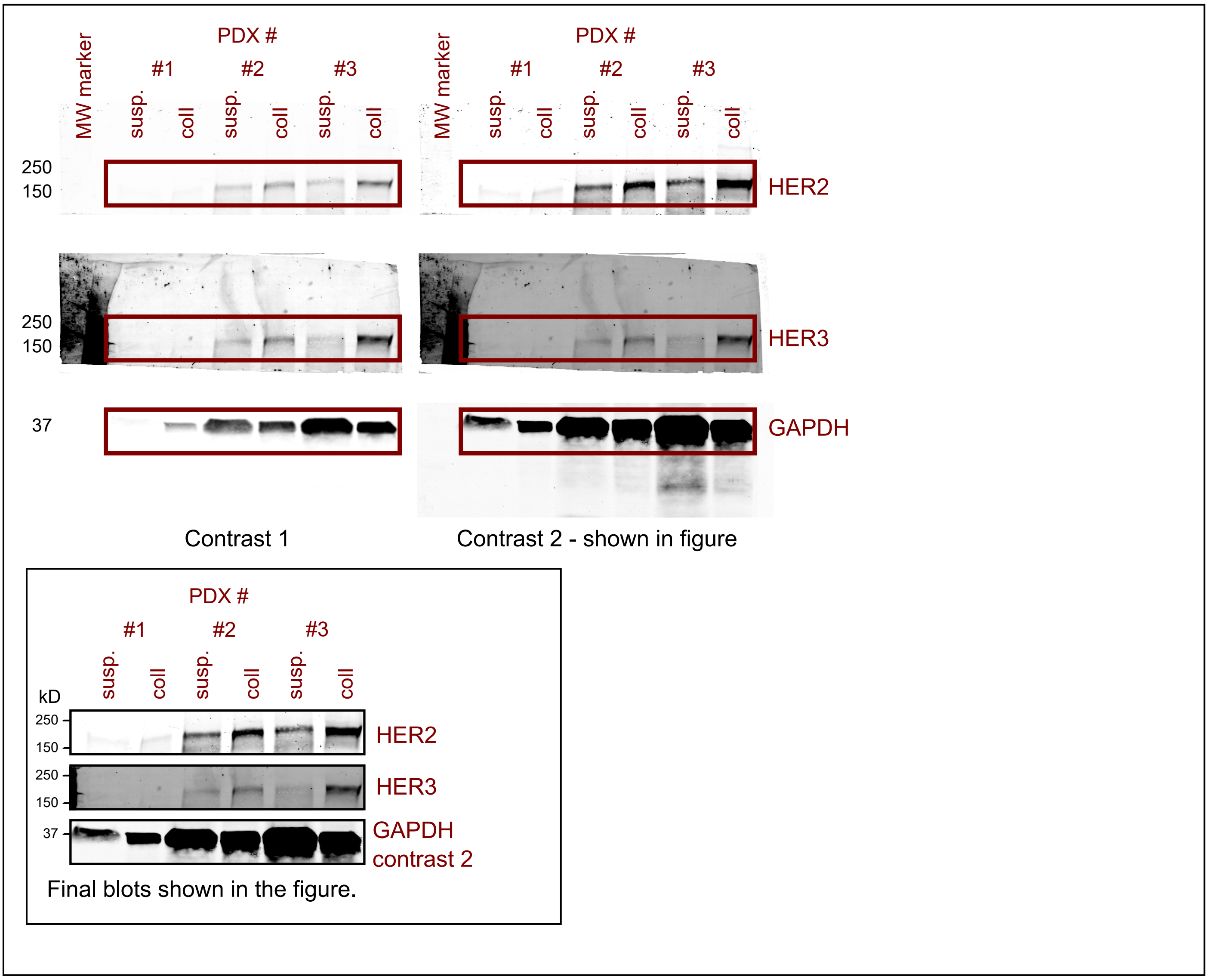

Figure S5 D

HER2 and HER3 levels in PDX#3 + AIIB2

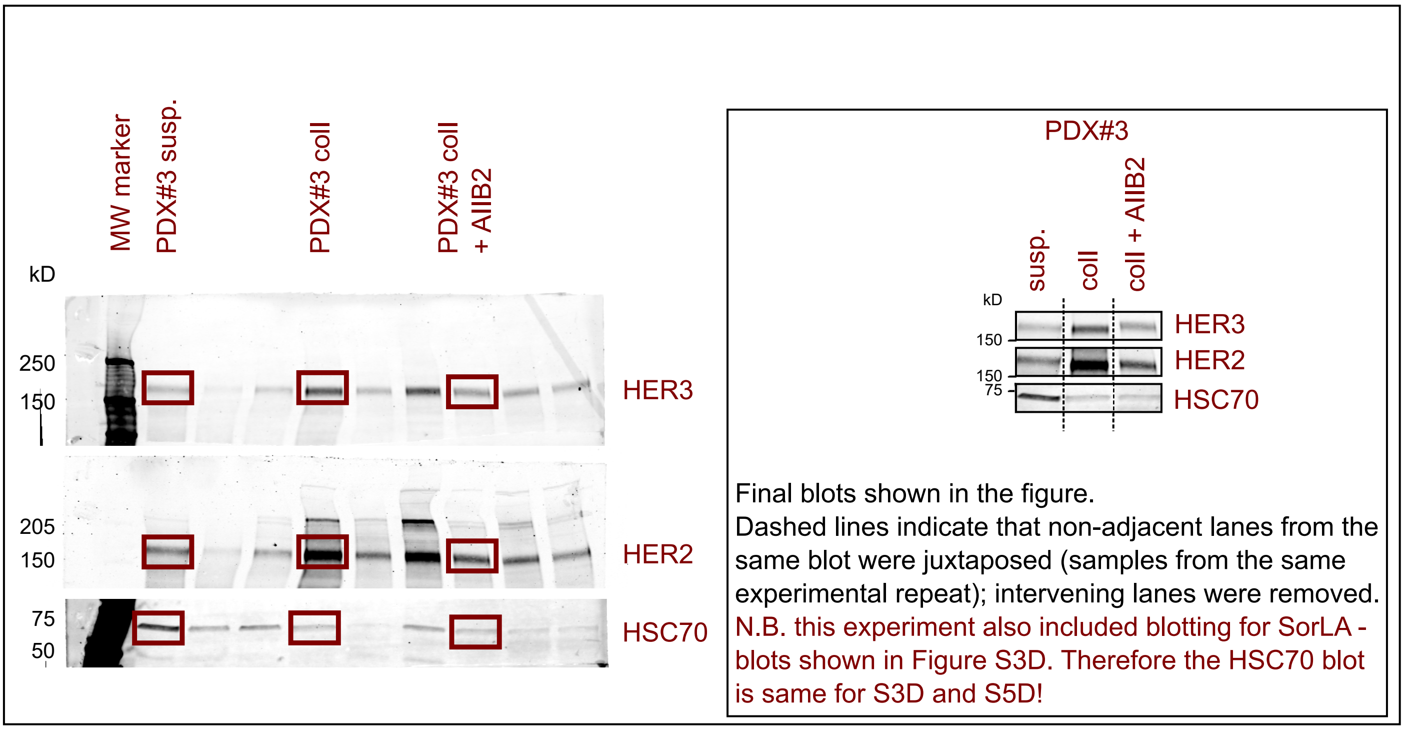

Figure S5 E

SorLA levels in PDX #3 + Tz and Pz

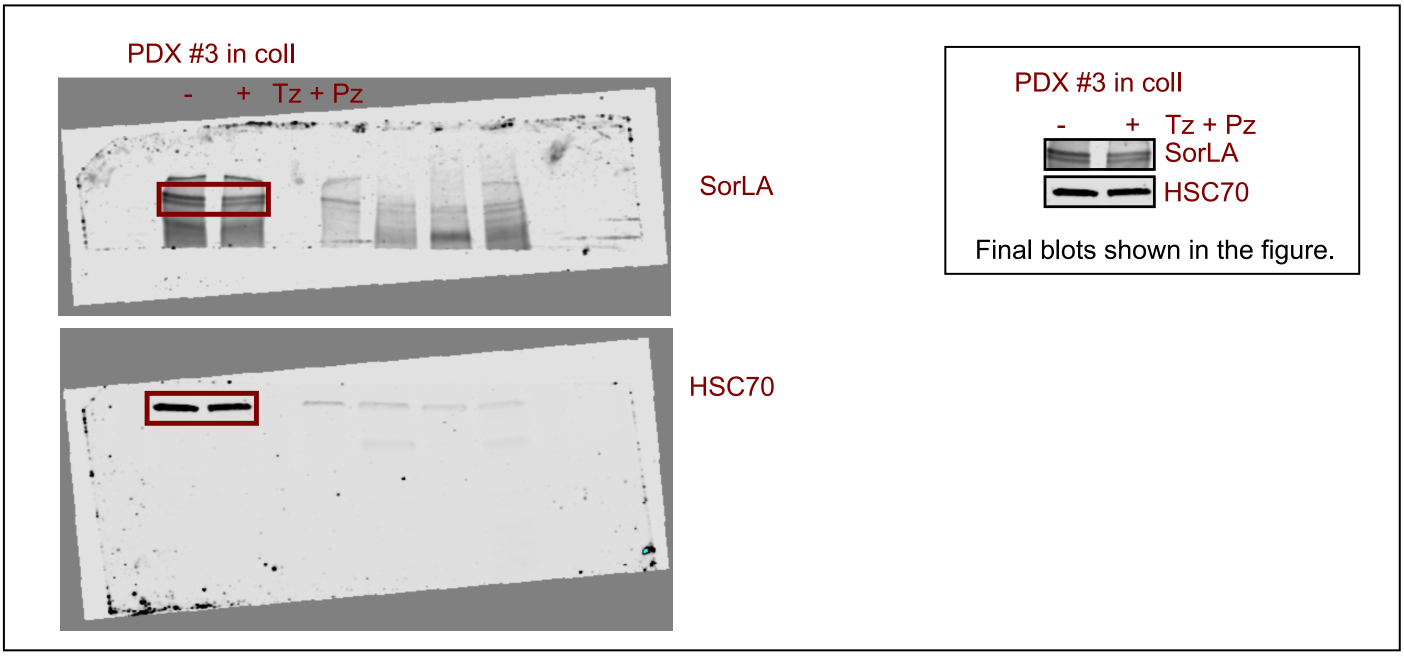

Figure S5 F

SorLA levels in PDX #2 + Hrg-β1

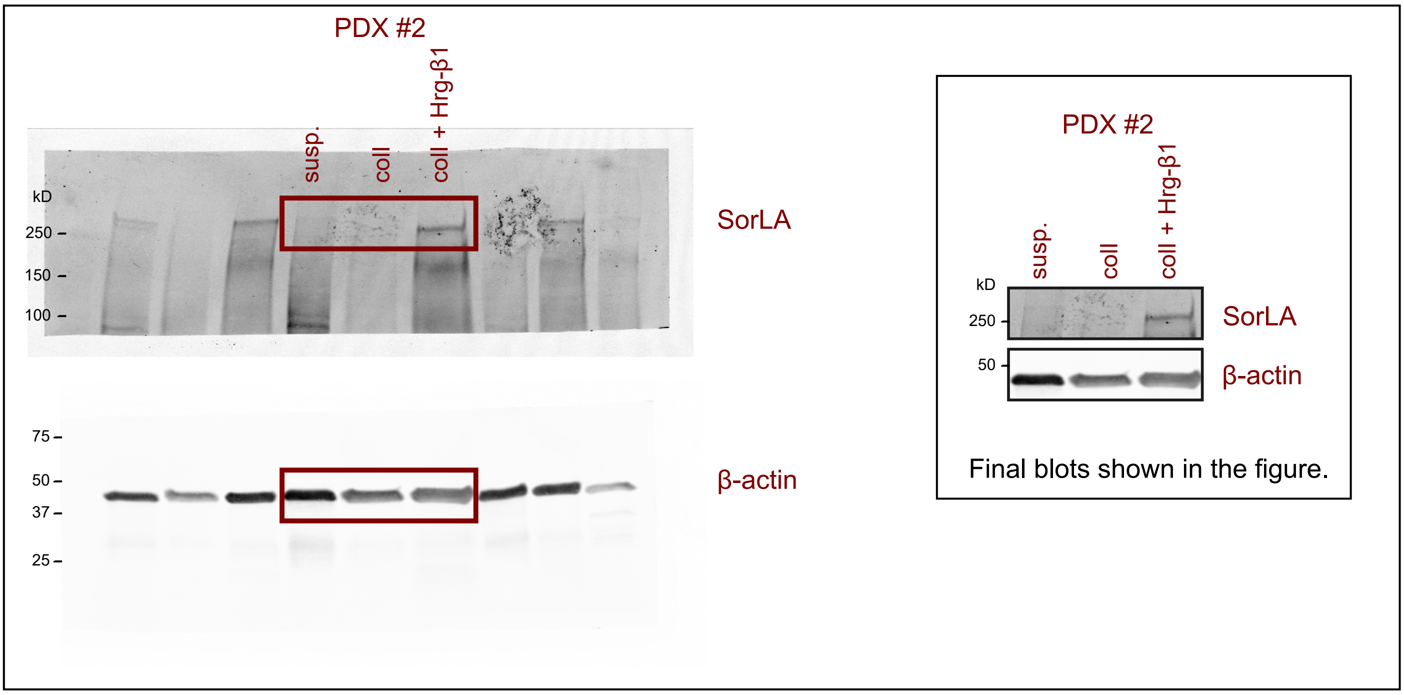

Supplement: Supplementary file 1 [file 41467_2026_75127_MOESM1_ESM.pdf]
