# Supplementary material for: Signaling downstream of tumor–stroma interaction regulates mucinous colorectal adenocarcinoma apicobasal polarity
Source: Nat Commun. 2026 Jul 4;17:8303. doi: 10.1038/s41467-026-75127-0 (PMC13469138; doi:10.1038/s41467-026-75127-0)
Supplement: Supplementary file 2 [file 41467_2026_75127_MOESM2_ESM.pdf]

Description of Additional Supplementary Files

Supplementary Movie 1: Live imaging of PDX#3 tumoroid polarity reversion following embedding in fluorescently labelled collagen over 15 hours. Tumoroids were stained with SiR-actin for visualization. Related to Figure 1C and S2J.

Supplementary Movie 2: Live imaging of PDX#3 polarity reversion following embedding in fluorescently labelled collagen over 15 hours, treated with AIIB2 (1 µg/ml), starting from timepoint 0 (collagen embedding). Tumoroids were stained with SiR-actin for visualization. Related to Figure S2I and S2J.
