# Supplementary material for: Signaling downstream of tumor–stroma interaction regulates mucinous colorectal adenocarcinoma apicobasal polarity
Source: Nat Commun. 2026 Jul 4;17:8303. doi: 10.1038/s41467-026-75127-0 (PMC13469138; doi:10.1038/s41467-026-75127-0)
Supplement: Supplementary file 3 [file 41467_2026_75127_MOESM5_ESM.pdf]

nature portfolio

Corresponding author(s): Johanna Ivaska

Last updated by author(s): Jun 5, 2026

Reporting Summary

Nature Portfolio wishes to improve the reproducibility of the work that we publish. This form provides structure for consistency and transparency in reporting. For further information on Nature Portfolio policies, see our Editorial Policies and the Editorial Policy Checklist.

Statistics

For all statistical analyses, confirm that the following items are present in the figure legend, table legend, main text, or Methods section.

n/a	Confirmed	
☐	☒	The exact sample size (*n*) for each experimental group/condition, given as a discrete number and unit of measurement
☐	☒	A statement on whether measurements were taken from distinct samples or whether the same sample was measured repeatedly
☐	☒	The statistical test(s) used AND whether they are one- or two-sided<br>*Only common tests should be described solely by name; describe more complex techniques in the Methods section.*
☒	☐	A description of all covariates tested
☐	☒	A description of any assumptions or corrections, such as tests of normality and adjustment for multiple comparisons
☐	☒	A full description of the statistical parameters including central tendency (e.g. means) or other basic estimates (e.g. regression coefficient) AND variation (e.g. standard deviation) or associated estimates of uncertainty (e.g. confidence intervals)
☐	☒	For null hypothesis testing, the test statistic (e.g. *F*, *t*, *r*) with confidence intervals, effect sizes, degrees of freedom and *P* value noted<br>*Give P values as exact values whenever suitable.*
☒	☐	For Bayesian analysis, information on the choice of priors and Markov chain Monte Carlo settings
☒	☐	For hierarchical and complex designs, identification of the appropriate level for tests and full reporting of outcomes
☒	☐	Estimates of effect sizes (e.g. Cohen's *d*, Pearson's *r*), indicating how they were calculated

Our web collection on statistics for biologists contains articles on many of the points above.

Software and code

Policy information about availability of computer code

Data collection

Data was collected as indicated in the materials and methods. All instruments used were commercially available and controlled using software provided by the manufacturer, unless otherwise stated.

Data analysis

Data analyses were performed as described in the methods. GraphPad Prism (versions 9.3.1 and 10.1.1) was used primarily for statistical analyses.

Normalized gene expression values were used to perform unsupervised hierarchical clustering using Euclidean distance and Ward.D2 linkage, and heatmaps were generated with the pheatmap R package with row scaling.
For differential expression analysis, the limma package was applied to the same ITGB1?trafficking gene set, comparing cells cultured on collagen versus cells in suspension after filtration. Linear models were fitted with empirical Bayes moderation, and genes were considered significantly differentially expressed at a false discovery rate (FDR) ≤ 0.1 after a moderated t-test and the Benjamini-Hochberg adjustment method was implemented in the R package limma. Volcano plots were generated from the limma output.

The custom image processing pipeline used to analyse the patient IHC data is available on Zenodo (https://doi.org/10.5281/zenodo.20156260).

For manuscripts utilizing custom algorithms or software that are central to the research but not yet described in published literature, software must be made available to editors and reviewers. We strongly encourage code deposition in a community repository (e.g. GitHub). See the Nature Portfolio guidelines for submitting code & software for further information.

Data

Policy information about availability of data

All manuscripts must include a data availability statement. This statement should provide the following information, where applicable:

- Accession codes, unique identifiers, or web links for publicly available datasets
- A description of any restrictions on data availability
- For clinical datasets or third party data, please ensure that the statement adheres to our policy

Data and material availability:
Data supporting the findings of this study are available within the paper and its supplementary information files or from the corresponding author upon reasonable request. The microarray data are from a previous publication (REF21) and are available on the Gene Expression Omnibus with the accession number GSE152299. WES data have been deposited in the NCBI Sequence Read Archive (SRA) under accession number PRJNA1473190.
Source data are provided with this paper.

Code availability:
The custom image processing pipeline used to analyse the patient IHC data is available on Zenodo (https://doi.org/10.5281/zenodo.20156260).

Research involving human participants, their data, or biological material

Policy information about studies with human participants or human data. See also policy information about sex, gender (identity/presentation), and sexual orientation and race, ethnicity and racism.

Reporting on sex and gender	Human samples were not collected specifically for this work.<br><br>PDX samples were from a previous study (Julien et al., 2012, Clinical Cancer Research, https://doi.org/10.1158/1078-0432.CCR-12-0372). This is indicated in the main text and methods section.<br><br>The patient data can be found in Table 1 of Julien et al., 2012.
Reporting on race, ethnicity, or other socially relevant groupings	NA
Population characteristics	See Table 1 in the Julien et al., 2012, Clinical Cancer Research, publication; https://doi.org/10.1158/1078-0432.CCR-12-0372
Recruitment	As indicated in this publication (Julien et al., 2012), CRC samples were collected, after patient's informed consent, in three medical centers: Curie Institute (Paris, France), Gustave Roussy Institute (Villejuif, France), and Lariboisière Hospital (Paris, France). Tumours were processed immediately after surgery.<br><br>In addition, the PDOs used in this study were initially derived from fresh tumor tissue samples collected within two precision medicine studies: ORGANOTREAT (NCT05267912) and Gustave Roussy Cancer Profiling STING trial (NCT04932525), both approved by the Gustave Roussy institutional review board and ethics committee (Cartry et al., 2023; Boilève et al., 2024), in compliance with the Declaration of Helsinki. All patients gave signed and informed consent for tumor sampling and collection and for genomic analysis.
Ethics oversight	reported in Julien et al., 2012, Clinical Cancer Research, https://doi.org/10.1158/1078-0432.CCR-12-0372. Patients gave signed and informed consent for tumor sampling and collection and for genomic analysis.

Note that full information on the approval of the study protocol must also be provided in the manuscript.

Field-specific reporting

Please select the one below that is the best fit for your research. If you are not sure, read the appropriate sections before making your selection.

☒ Life sciences ☐ Behavioural & social sciences ☐ Ecological, evolutionary & environmental sciences

For a reference copy of the document with all sections, see nature.com/documents/nr-reporting-summary-flat.pdf

Life sciences study design

All studies must disclose on these points even when the disclosure is negative.

Sample size	Experiments were performed at least three times, unless otherwise stated. For imaging data, representative images are shown. For experiments with < 3 independent replicates, sufficient sample sizes (cells, measurements, etc) were used to ensure results are scientifically

	relevant.
Data exclusions	NA
Replication	To ensure reproducibility, experiments were replicated at least three times (unless otherwise stated). Key experiments were performed at different times and with different batches of cells, for example.
Randomization	The experiments were not randomized. However, animals were randomly assigned to cages by the animal facility staff. Mice assigned to different experimental conditions were run in parallel and all animals were maintained under the same condition and were at the same developmental stage. For IF imaging, images were taken of multiple fields from the same sample.
Blinding	The experiments were not performed in a blinded fashion, with the exception of the analysis of the in vivo models of PDX#3 tumors treated with trastuzumab/pertuzumab or IgG control. In this case, the analyst was blind to the treatment condition. Analysis software/statistical packages were used as detailed in the methods for robust data analysis, removing user bias. In addition, appropriate controls were included in experiments and control versus treated samples were analyzed in the same fashion.

Reporting for specific materials, systems and methods

We require information from authors about some types of materials, experimental systems and methods used in many studies. Here, indicate whether each material, system or method listed is relevant to your study. If you are not sure if a list item applies to your research, read the appropriate section before selecting a response.

Materials & experimental systems

n/a	Involved in the study	
☐	☒	Antibodies
☐	☒	Eukaryotic cell lines
☒	☐	Palaeontology and archaeology
☐	☒	Animals and other organisms
☒	☐	Clinical data
☒	☐	Dual use research of concern
☒	☐	Plants

Methods

n/a	Involved in the study	
☒	☐	ChIP-seq
☒	☐	Flow cytometry
☒	☐	MRI-based neuroimaging

Antibodies

Antibodies used	All antibody information including name, source, dilution, cat. No. etc., are provided in Supplementary Table 2, which has been referenced in the Methods.
Validation	Antibodies were not separately validated by us but have been validated by the manufacturer and used routinely by others and us. The manufacturer catalogue numbers are provided in Supplementary Table 2. In addition, siRNA knockdown experiments included in the manuscript were used as a form of validation of the anti-SorLA antibody.

Eukaryotic cell lines

Policy information about cell lines and Sex and Gender in Research

Cell line source(s)	Cell lines were acquired from ATCC (LS513)
Authentication	None of the cell lines were separately authenticated by the authors
Mycoplasma contamination	Cell lines used in this study were regularly tested and confirmed negative for mycoplasma.
Commonly misidentified lines (See ICLAC register)	No commonly misidentified cell lines were used (ICLAC register version 13).

Animals and other research organisms

Policy information about studies involving animals; ARRIVE guidelines recommended for reporting animal research, and Sex and Gender in Research

Laboratory animals	NOD.Cg-Prkdcscid Il2rgtm1WjI/SzJ, female, 6-8 weeks old
Wild animals	NA
Reporting on sex	Although only female mice were used, gender was not a consideration in this study and no gender-specific claims are being made.

Field-collected samples	NA
Ethics oversight	Animal experiments performed in France were compliant with French legislation and EU Directive 2010/63. The project was validated by the Ethical Committee (CEEA) no. 26 and was then granted French government authorizations under number 517-2015042114005883 and 2734-2015111711418501. Animal experiments performed in Finland were in accordance with the Finnish Act on Animal Experimentation (animal license numbers ESAVI/12558/2021 and ESAVI/6253/2024). Mice were obtained from Charles River France and Germany, housed and bred at the Gustave Roussy animal core facility (accreditation number E-94-076-11) and at TCDM (Turku Center for Disease Modelling). Mice were housed in standard conditions (12-hour light/dark cycle; ambient temperature, 21oC; 50% humidity ± 8%) with food and water available ad libitum. Animals were euthanized according to endpoints that were validated by the Ethical Committee, the French government (Ministère de l'Enseignement Supérieur, de la Recherche et de l'Innovation) and the Finnish government (Etelä-Suomen Aluehallintovirasto, hankelupatoimikunta).

Note that full information on the approval of the study protocol must also be provided in the manuscript.

Plants

Seed stocks	NA
Novel plant genotypes	NA
Authentication	NA
