# Supplementary material for: Signaling downstream of tumor–stroma interaction regulates mucinous colorectal adenocarcinoma apicobasal polarity
Source: Nat Commun. 2026 Jul 4;17:8303. doi: 10.1038/s41467-026-75127-0 (PMC13469138; doi:10.1038/s41467-026-75127-0)
Supplement: Supplementary file 4 [file 41467_2026_75127_MOESM6_ESM.pdf]

Peer Review File

Signaling downstream of tumor-stroma interaction regulates mucinous colorectal adenocarcinoma apicobasal polarity

Corresponding Author: Professor Johanna Ivaska

This file contains all reviewer reports in order by version, followed by all author rebuttals in order by version.

Version 0:

Reviewer comments:

Reviewer #1

(Remarks to the Author)
In this manuscript, Nicolas et al. investigate the underlying mechanisms by which tumor-stroma interactions regulate apical-basal polarity in mucinous colorectal carcinoma (MUC CRC). The authors demonstrate that collagen–α2β1-integrin interaction activates Src and ERK/MAPK signaling, leading to the upregulation of SorLA. This upregulation supports the maintenance of apical-in polarity by promoting integrin recycling, thereby establishing a positive feedback loop that influences tumor progression. The study further shows that blocking HER2/HER3 reverses apical-in polarity and reduces adhesion to the peritoneum.
Overall, the manuscript presents an interesting and generally robust piece of work. Several conclusions could be further strengthened with additional experimental support.
1. Based on the Ezrin and F-actin staining in Figure 1A and 1D, the collagen-embedded PDX#3 organoids display Ezrin and F-actin staining both in the inner lumen and the outer membrane. This suggests that they are not typical apical-in organoids. Instead, it appears that the organoids form multiple layers of cell clusters, where the inner cell layers exhibit apical-in polarity, while the outer cell layers display apical-out polarity. Please clarify the definition of apical-in polarity.
2. To support the conclusion in the abstract that “Collagen–α2β1-integrin interaction activates Src and ERK/MAPK signaling,” the authors should assess the levels of p-Src and p-ERK in AIIB2- or P1E6-treated PDX#3 samples from Figure 2D–E. This would help to strengthen whether α2β1-integrin mediates the activation of Src and ERK/MAPK signaling.
3. Fig.S2D. It is not obvious that expression of integrin α2 or β1 is altered in response to collagen I culture from these graphs. Please provide quantification.
4. Fig. 1C and Video 1 shows that clear formation of lumen-like structures and protrusions could be observed as early as 5 hours post-embedding. It would be important to determine how quickly upregulation of SorLA and maturation of integrin β1 occurs to contribute to the phenotype.
5. Page6, “Indeed, we detected significantly elevated ERK phosphorylation in PDX#3 tumoroids in collagen (Fig. 3H and S3C) and collagen-induced SorLA upregulation was sensitive to integrin function-blocking antibodies (Fig. 3I and S3D).” This conclusion could be strengthened by inhibiting ERK and confirming that the ERK pathway is responsible for the upregulation of SorLA mRNA.
6. Page 6, “These data suggest the possibility that the PDX#3 tumoroids in collagen switch from RCP-mediated long-loop integrin recycling to a SorLA-driven rapid recycling pathway in a manner that is dependent on initial matrix-induced integrin signaling.” requires additional experimental support. The authors should examine the colocalization of RCP or SorLA with integrin proteins in suspension vs. collagen-embedded organoids to support this conclusion.
7. Page 8, “Conversely, HER2/HER3 activation with the HER3 ligand Heregulin- β1 was sufficient to trigger collagen-induced polarity reversion in the thus far matrix agnostic PDX#2 tumoroids (Fig. 5H-I). Upon HER3 stimulation with Heregulin- β1, SorLA was also found to be upregulated in PDX#2 (Fig. 5J and S5E). The PDX#1 tumoroids did not respond, presumably due to their lack of HER3 expression (Fig. 5B and S5C-D).” Since the authors suggest that HER2/HER3 activation or collagen embedding induces polarity reversion by upregulating SorLA, could SorLA overexpression alone trigger collagen-induced polarity reversion in collagen-embedded PDX#1 or PDX#2 organoids?
8. Figure 6G does not clearly display apical-in polarity. Please provide representative images that better illustrate the apical-in phenotype.

Reviewer #2

(Remarks to the Author)
In this paper, the author investigated the mechanism of polarity control in MUC CRC tumors. The authors found that sensing collagen by integrin α2β1 promotes the expression of SorLA, which in turn promotes integrin recycling. HER2/HER3 upregulates the expression of SorLA. The mechanisms of polarity switching in tumors are of great interest to cell biologists. However, there are concerns that the authors should clarify in this study.

Major comments
1. This paper consists of two different themes. One is the collagen sensing system in CRC cell clusters. Another is the biological differences between CRC clusters with apical-in and apical-out polarity. In the collagen-sensing system part, the authors found that integrin α2β1 promotes SorLA expression, contributing to integrin rearrangement. In characterizing apical-in and apical-out polarity, the authors found differences in collagen fiber rearrangements and HER2/HER3 signaling. Both are interesting results but should be studied more deeply as independent studies, as they have very different content. The earlier subject is the sensing and execution of polarity switching, while the later subject is the consequence of polarity switching. It is difficult to summarize as an 'axis'.
2. It is an excellent discovery that integrin α2β1 is a sensor of collagen for polarity switching from apical-out to apical-in in PDX#3 tumoroids, but it may be specific to this case with exceptionally high β1 integrin levels. Indeed, it is a good model for studying the sensing system but the universality of the findings is not convincingly demonstrated.
Individual comments
3. The scoring system for quantifying polarity status should be described in detail in the Materials and Methods section, or it should be cited from a previous publication. According to the results section, the authors used Ezrin to calculate the polarity score. In this paper, either F-actin or Ezrin, or both, are used as indicators of the apical membrane. However, in many images, especially Figure 5H, the localization of F-actin and Ezrin is not the same. Please discuss the discrepancy of the staining of F-actin and Ezrin, and also show the score by F-actin.

4. The polarity score for the PDX#3 tumoroids embedded in collagen was mostly 2. In most of the control images of PDX#3 tumoroids in collagen, F-actin and Ezrin were detected in both the apical and inner membranes. Do the images represent the score? It would be better to show a score in each image.

5. Only three CRC PDX lines (2 polarity un-switchable, 1 polarity switchable) were used in this study. Due to the small number of samples, it is extremely important to validate the findings. Multiple organoid lines should be investigated for validation. It may be difficult to obtain additional PDX lines, but please explain why this study should be conducted with PDX tumoroids or PDOs rather than with more readily available CRC organoid lines.

6. To show that 'SorLA-dependent Integrin-β1 trafficking is induced by HER2/HER3 signaling', it should be shown that inhibiting HER2/3 suppresses the expression of SorLA and integrin β1 in PDX#3. In addition, it should be shown whether HRG induces integrin β1 in PDX#2.

7. For evaluating the interaction between CRC tumoroids and the peritoneum, the model here (Fig. 6) is not suitable. In the process of epithelial cells becoming malignant, invasion into the basement membrane is an important process. On the other hand, peritoneal invasion of colorectal cancer is a completely different phenomenon. Cancer cells first come into contact with peritoneal cells, and then invade the basal membrane after pushing them away. Therefore, the model should be peritoneal cells, not the basement membrane.

8. It should be shown that SorLA-silenced or HER2/3-inhibited PDX#3 show apical-out phenotype in vivo.

9. Overall, the discussion is not well written. Discussion should not be a repetition of results. Some of the contents of the Discussion have already been described in the results section.
10. Please define the terms 'tumoroid', 'spheres', and 'PDO' in the text or Materials and Methods section.
11. Introduction: 'the TGF-β pathway has been implicated (Okuyama et al., 2016, Onuma et al., 2021, Canet-Jourdan et al., 2022).' Canet-Jourdan et al. argued that the TGF-β pathway is involved in polarity switching and that Rho is downstream of this, but Okuyama and Onuma et al. do not mention TGF-β, and instead clarified that Rho is involved in polarity switching.
12. Materials and methods: Generation of tumoroids. They cited Sato et al, 2011, but there is no description of tumoroid preparation in the reference. Rather, it seems to have been first described in doi/10.1073/pnas.1015938108.
13. P4 line 9: What does 'dynamic contact' mean?

14. Bright field images in Figure 1C and Figure S2E are too dark to see the details.

15. P5 line 3: 'In PDX#3 tumoroids, the localization of β1-integrins (detected with the P5D2 antibody) shifted from lateral (cell-cell) to basal (cell-ECM)' is not true. The staining is clearly seen in basal in suspension.

16. Figure 2A: The pseudo color for 12G10 should not be yellow because the colocalized staining of red (P5D2) and green (F-actin) will be also yellow.

17. The change of staining pattern of β1 integrin should be shown in all the latter results.

18. Figures 2F and 4F should be removed because some of the data for each scheme is not displayed in the respective figures. This is related to the logical problem, syllogisms, in this manuscript. Contents of the scheme, if any, should be restricted to the contents of the figure.

19. P3 last line: ‘66% of patient-derived TSIPs retain their apico-basolateral polarity’. Does this mean ‘66% of patient-derived TSIPs derived from MUC CRC patients retain their apico-basolateral polarity’?

20. P7 second paragraph: Please explain what are ‘the technical challenges associated with performing cell biology with PDX models’ in detail. Are you referring to the ‘cell-surface biotinylation-based integrin uptake assay’? If so, please explain why it cannot be performed with PDOs.

21. P7 second paragraph: ‘secrete’ is not an appropriate verb here. ‘discharge’?

22. The last paragraph is not an appropriate conclusion in this section.

23. Figure 5B: In the Western blotting shown, both HER2 and HER3 are elevated in PDX#2. The quantitative analysis states that there is no significant difference in HER3, but as the number of trials is small and the values fluctuate greatly, it is difficult to draw conclusions based on the significance of this experiment alone. It would be better to discuss the possibility that factors other than HER2/HER3 are involved in this phenomenon.

24. Figure 5G: The difference is marginal and there is a synergistic effect when Tz and Pz are used together. From these results, it is difficult to say ‘indicating a functional role for HER2/HER3 signaling, but not HER2/HER2 homodimers’.

25. Figure 5J, 7D, 7E: There are strange shapes in the graph.

26. P10 first paragraph is not an appropriate conclusion in this section.

27. Fig.7A: Staining of apical markers should be shown.

Reviewer #3

(Remarks to the Author)
Pasquier and colleagues present an intriguing model aimed at explaining polarity dynamics in mucinous colorectal adenocarcinoma. Overall, the experiments seem to be well-conducted, although it is important to note that the majority of observations supporting the main conclusions of the paper stem from a single patient-derived xenograft (PDX) model that aligns with the authors' proposed model. I have several questions regarding the screening of different integrins in the PDX models and the validation of their findings in human samples:

1. In relation to the data presented in Figure S2, which positive control was included to substantiate the claim that several immunodetections for the various integrins are indeed negative?
2. Concerning the data shown in Figure 7 and Figure S7 for human tumors, I do not recognize the histology as mucinous, particularly in the apical-in image. Could the authors provide images that include larger areas of tissue to clearly demonstrate that these tumors are indeed mucinous? Additionally, it would be helpful to see the original IHC images without normalization.
3. If I am correct, HER2 or HER3 expression should not be elevated in normal colonic tissue, and such expression is not commonly observed in colon adenocarcinoma. The authors should address this issue.
4. While the authors have validated their model using 25 mucinous adenocarcinomas, it would be logical to also include non-mucinous tumors for comparison.

Reviewer #4

(Remarks to the Author)

Mucinous CRC tumors are in general a poor-prognosis tumor subtype because of many different reasons. In this study, the authors focus on studying the mechanisms that support a phenotype that is present in this particular cancer subtypes, which is the presence of an inverted polarity (polarity-out) that can be altered upon interaction of tumor cells with the stroma. Although I can understand that the study is relevant, I have major concerns that should be taken into consideration:

1- Most of the study is done using 3 different PDX lines, with only one of them showing the phenotype of interest, which is the reversion of the polarity-out phenotype upon interaction with collagen/stroma. I would suggest having two more PDX lines displaying the same phenotype as PDX3 to be able to draw robust conclusions from the observations.

2- In addition, I cannot agree with the conclusion that PDX3 flips from apical-out to apical-in when in contact with collagen. From the results shown, the apical-out polarity is clearly maintained in all conditions and in all PDXs, with the only difference being that small lumens seem to appear in PDX3 when adhered to the plate/collagen matrix (although only one spheroid is shown), and in the human tumors shown in Figures 7 and S7. In my opinion, the main difference between PDX1-2 and PDX3 is the ability to adhere to the plate in the collagen cultures which should be further explored. Therefore, I don’t think the concept of apical-out to apical-in shouldn't be used.

3- Although I can understand that the study is relevant, I'm not entirely convinced that culturing cells in collagen is sufficient to mimic the interaction between cancer cells and the stroma. Additional matrixes need to be added in the experiments. In

addition, the collagen cultures contain 10% FBS, which in our hands is sufficient to promote organoid adhesion and spreading on plastic plates in the absence of collagen. Have the authors tested whether PDX3 needs collagen to acquire the adhesive/apical-in phenotype? Moreover, it is not clear from the methods what the non-adhesive medium contains. Is it the same medium as the one used to grow cells in collagen?

4- Authors have performed whole genome sequencing of the three PDXs. Since TGFb is involved not only in the polarity responses to the stromal environment, as the authors explain, but also in migration and adhesion processes, is any gene involved in the TGFb pathway mutated in the PDXs that could explain the differences in behavior on collagen culture?

5- Regarding the secretion of mucins, only one (not very conclusive) image of a lectin staining is shown in S1B without any quantification. Additional IF/IHC for specific mucins and some quantification could support authors' conclusions. I have also noticed that the mucin staining is no longer shown in the experimental conditions tested in the other figures, which would be informative and supportive.

6- In Figure 3, authors perform RNA-seq to identify putative regulators of integrin b1 trafficking. However, western blot levels of RCP poorly represent what is obtained by RNA-seq (RCP expression is much higher in PDX1 than in the others) and the western blot shown does not match with the values in the quantification. Moreover, the KD of SorLA is minimal and the WB is of very low quality. The same problem is found in the WB analyses in 5A-B, where the GAPDH control is absolutely variable between samples, making the quantification presented scarcely credible. In general, some of the conclusions drawn are not entirely correct, which does not invalidate the results obtained, but makes it difficult to follow the story (a constant comparison of what is said in the text and what is shown in the figures is necessary).

7- Then p-ERK appears in the scenario as a downstream effector of integrin/ECM engagement, but only p-ERK levels from PDX3 are shown. The authors should show p-ERK in the other PDXs to be able to conclude that this is something specific to the PDX3 phenotype and not something unrelated.

8- HER2-3 could be better integrated in the final model since the authors have previously shown the role of SorLA in HER2 regulation in breast cancer.

In general, although I find the study interesting, the data presented do not support the authors' conclusions.

Reviewer #5

(Remarks to the Author)
I co-reviewed this manuscript with one of the reviewers who provided the listed reports. This is part of the Nature Communications initiative to facilitate training in peer review and to provide appropriate recognition for Early Career Researchers who co-review manuscripts.

Version 1:

Reviewer comments:

Reviewer #1

(Remarks to the Author)
The revision has addressed several points raised in previous review. It is understandable that some concerns, such as Point #7 is technically challenging to address. However, two key points #4 and #6 remain unanswered and requires additional data support. These two points are critical to support the main model that collagen-induced α2β1-integrin engagement upregulates SorLA, which drives rapid β1-integrin recycling to trigger polarity reversion. Current data show that SorLA is involved in collagen-induced polarity reversion, but it is somewhat uncertain whether this is due to the role of SorLA in integrin recycling. Answering even one of these two points could provide some much-needed support.

Previous point #4: Fig. 1C and Video 1 shows that clear formation of lumen-like structures and protrusions could be observed as early as 5 hours post-embedding. It would be important to determine how quickly upregulation of SorLA and maturation of integrin β1 occurs to contribute to the phenotype.
This is indeed an interesting point. Our data show that only PDXs and PDOs with initially high integrin and SorLa levels (PDX#3 vs the others, PDO#2 vs PDO#1) are able to engage the matrix and generate protrusions. Therefore, the initial response is most likely mediated by the already high integrin, and, then, reinforced by SorLA upregulation and integrin maturation in long-term culture.
As shown in Fig. 3E, even though PDX#3 has high integrin α2β1, the expression of SorLA is dependent on collagen binding as PDX#3 in suspension shows low SorLA expression similar to in PDX #1 and PDX#2. Could integrin α2β1 and SorLA levels be monitored by IF following collagen embedding to support this claim? To conclude that SorLA upregulation by integrin α2β1 activation is required for polarity reversion, it is critical to determine the spatiotemporal pattern of integrin α2β1 and SorLA protein post-embedding to promote polarity reversion. This is especially critical since the current data do not directly show that SorLA regulates β1-integrin recycling in PDX#3, therefore missing the link between SorLA in β1-integrin recycling and SorLA in polarity reversion.

Previous Point 6. Page 6, “These data suggest the possibility that the PDX#3 tumoroids in collagen switch from RCPmediated long-loop integrin recycling to a SorLA-driven rapid recycling pathway in a manner that is dependent on initial matrix-induced integrin signaling.” requires additional experimental support. The authors should examine the colocalization of RCP or SorLA with integrin proteins in suspension vs. collagen-embedded organoids to support this conclusion.
We agree with the reviewer that this conclusion was not fully supported by the data. We have unfortunately been unable to obtain high-quality immunofluorescence staining of RCP in the collagenembedded tumoroids. In response to the criticism, we have edited the text to: “These data suggest the possibility that the polarity switching of PDX#3 tumoroids in collagen is supported by ERK-induced SorLA expression” (page 10).
Since the role of SorLA in integrin recycling was only demonstrated in CRC cell line LS513, but not in PDX#3, the conclusion “Apical-in polarity in collagen is established through SorLA-dependent β1-integrin recycling” requires such experimental support. RCP IF is not feasible, but SorLA IF appears to be feasible and could be used to study its colocalization with integrin proteins in suspension vs. collagen-embedded condition.

Reviewer #2

(Remarks to the Author)
The authors responded appropriately to my comments. Following are my additional comments.
I commend the authors' efforts to add other organoids and present organoids exhibiting a similar response to PGX#3. However, the number remains small. The authors should confine their assertion to stating that ‘this mechanism exists in some MUC CRC patients.
The differences of the polarity score are marginal in some of the experiments. In such cases, other pathways are also likely to be involved. The authors should state that the molecular mechanisms underpinning polarity reversal may be more complex and diverse.
I commend the authors' efforts in adding trastuzumab and pertuzumab treatment in vivo. Indeed, changes in polarity and cell death are observed. Although the pattern of change is localized, I consider it worthy of future investigation to understand why such a patterned change occurs.
I still cannot agree to the use of the collagen infiltration assay as a model for peritoneal seeding. I fully concur that exposure of the peritoneal basement membrane promotes dissemination. It would be preferable to present the basement membrane infiltration assay as it is, without using it as a peritoneal seeding model, and to discuss it solely as a factor promoting peritoneal seeding within the discussion section.

Reviewer #3

(Remarks to the Author)
I thank the authors for addressing my comments. I would just recommend that the authors interpret the data from the Human Protein Atlas with caution. Not all clones that have been tested are performing well. For instance, the HER3 IHC that was included in the reply to the Reviewers clearly lacks specificity.

Reviewer #4

(Remarks to the Author)
In this revised version of the manuscript, the authors have included additional data to address some of the initial concerns raised by the reviewers. They have provided a more detailed definition of the apical-in phenotype and incorporated an additional model with characteristics similar to PDX3. However, in my view, several critical issues still require adequate clarification and resolution.
PDX models and phenotype:
While it is understandable that increasing the number of PDX models may not always be feasible, particularly when dealing with rare or specific phenotypes, it remains unclear how many PDXs have been analyzed beyond those presented in the manuscript. It would be important to know what proportion of the total tumors evaluated display this specific phenotype, in order to assess its prevalence and biological relevance.
Culture conditions:
Based on our experience, when cultured in DMEM supplemented with 10% FBS, even PDOs growing in 3D tend to sink and adhere to the plate, thereby resembling the phenotype shown by the authors and defined here as “apical-in”. Although our observations were made using Matrigel, it would be helpful to include lower-magnification images to provide a more comprehensive view of what is occurring in these cultures. This concern also applies to Figure 1, where no spheres appear to be present in the non-coated cultures. To my knowledge, differences should primarily be reflected in the number of adhered cells or spheres, rather than in the total number of cells present in the cultures.
Alcian Blue staining:
It is positive that the authors have included Alcian Blue staining to visualize mucins, although the original request was to assess specific mucins (that could be selected based on RNA-seq data). That said, I have serious concerns regarding the quality of the staining presented. The background signal is extremely high, making it difficult to clearly discern what is being quantified. We have performed similar staining in CRC tumors, and I find it difficult to understand why such a high background is present in these samples. Could the authors optimize the staining protocol to improve the quality and interpretability of the mucin signal?
Western blot analyses:
I continue to have concerns regarding the western blot data, particularly given that the authors have not attempted to improve the quality of the results shown (although they say they tried). While I appreciate that sample availability may be limited, the

authors have had sufficient time to generate new material and lysates to provide more robust and reliable data to support their conclusions. In addition, the manner in which several blot panels have been cropped is problematic, as it hinders assessment of data consistency across replicates. Overall, the quality of the western blot data does not appear to meet the standards expected for publication in Nature Communications.
p-ERK results:
Regarding the p-ERK data, it is plausible that p-ERK is induced in PDX3, as suggested by the western blot shown in the initial submission. However, given the high variability observed between replicates, now clearly evident from the PDX1 and PDX2 data, it is reasonable to question whether p-ERK levels in PDX3 are truly less variable. To support this conclusion, equivalent biological replicate data for PDX3 should be presented.
Overall assessment:
In summary, several experimental results and their interpretations require further validation and clarification before robust conclusions can be drawn.

Reviewer #5

(Remarks to the Author)
I co-reviewed this manuscript with one of the reviewers who provided the listed reports. This is part of the Nature Communications initiative to facilitate training in peer review and to provide appropriate recognition for Early Career Researchers who co-review manuscripts.

Version 2:

Reviewer comments:

Reviewer #1

(Remarks to the Author)
This revision has addressed several critical concerns and weakened several statements based on exisiting results. The revision is satisfactory.

Reviewer #2

(Remarks to the Author)
The authors addressed my concerns quite appropriately.

Reviewer #3

(Remarks to the Author)
I have no further comments.

Reviewer #4

(Remarks to the Author)
The authors have now addressed all of my previous concerns.

Reviewer #5

(Remarks to the Author)
I co-reviewed this manuscript with one of the reviewers who provided the listed reports. This is part of the Nature Communications initiative to facilitate training in peer review and to provide appropriate recognition for Early Career Researchers who co-review manuscripts.

Open Access This Peer Review File is licensed under a Creative Commons Attribution 4.0 International License, which permits use, sharing, adaptation, distribution and reproduction in any medium or format, as long as you give appropriate credit to the original author(s) and the source, provide a link to the Creative Commons license, and indicate if changes were made.
In cases where reviewers are anonymous, credit should be given to 'Anonymous Referee' and the source.
The images or other third party material in this Peer Review File are included in the article's Creative Commons license, unless indicated otherwise in a credit line to the material. If material is not included in the article's Creative Commons license and your intended use is not permitted by statutory regulation or exceeds the permitted use, you will need to obtain permission directly from the copyright holder.
To view a copy of this license, visit https://creativecommons.org/licenses/by/4.0/

Point-by-point response to reviewer comments

Reviewer #1 (Remarks to the Author):

In this manuscript, Nicolas et al. investigate the underlying mechanisms by which tumor-stroma interactions regulate apical-basal polarity in mucinous colorectal carcinoma (MUC CRC). The authors demonstrate that collagen–α2β1-integrin interaction activates Src and ERK/MAPK signaling, leading to the upregulation of SorLA. This upregulation supports the maintenance of apical-in polarity by promoting integrin recycling, thereby establishing a positive feedback loop that influences tumor progression. The study further shows that blocking HER2/HER3 reverses apical-in polarity and reduces adhesion to the peritoneum.
Overall, the manuscript presents an interesting and generally robust piece of work. Several conclusions could be further strengthened with additional experimental support.

We thank the reviewer for carefully evaluating our work and for these encouraging and positive comments.

1. Based on the Ezrin and F-actin staining in Figure 1A and 1D, the collagen-embedded PDX#3 organoids display Ezrin and F-actin staining both in the inner lumen and the outer membrane. This suggests that they are not typical apical-in organoids. Instead, it appears that the organoids form multiple layers of cell clusters, where the inner cell layers exhibit apical-in polarity, while the outer cell layers display apical-out polarity. Please clarify the definition of apical-in polarity.

Thank you for this valuable comment. We fully agree that these are not typical apical-in organoids. Depending on the size of the patient-derived CRC organoids, we often found organoids with multiple cell layers. As such, their polarity differs from the typical example of one-cell layered MDCK cysts with a single lumen in the middle. Nevertheless, we definitely observe a spectrum of different polarity phenotypes that we scored using a single “polarity index” per organoid. This single measure is based on three parameters, defining three sub-scores: Sub-score 1) The presence/absence of lumen, set to 0 in case of lumen absence, or 1 in case of the presence of one or more lumen. Sub-score 2) Circularity, obtained through the shape descriptor function on ImageJ. A value of 0 would be obtained for a perfect sphere, whereas a value closer to 1 translates to a higher number of protrusions. The segmentation here is based on the actin fluorescence signal. Sub-score 3) The apicobasal ezrin signal ratio, which is the ratio of the ezrin luminal signal (or the mean of luminal signals in case of multiple lumen) to the total ezrin fluorescence (luminal+cortical). This value varies from 0 (complete cortical signal) to 1 (complete luminal signal). The sum of these three sub-scores is the total polarity score (PS) varying from 0 (perfect apical-out) to 3 (perfect apical-in). In case of non-polarized structures (cytoplasmic ezrin signal), we set the polarity score to 1.5 per default.

The presence of ezrin at the outer membranes (as correctly observed by the reviewer) is thus taken into account in our polarity index measurement. To clarify this, we have now defined apical-in polarity in the text (page 4) as follows “.......whereas PDX#3 tumoroids in collagen revert their polarity into a multilayered structure with features of apical-in polarity such as Ezrin-positive lumens and collagen-facing protrusions (Fig. 1A,B). While these multi-layered structures are not typical one-cell layered apical-in spheroids with a single and well-defined lumen, they will be referred to as apical-in from hereon”.

2. To support the conclusion in the abstract that “Collagen–α2β1-integrin interaction activates Src and ERK/MAPK signaling,” the authors should assess the levels of p-Src and p-ERK in AIIB2- or P1E6-treated

PDX#3 samples from Figure 2D–E. This would help to strengthen whether α2β1-integrin mediates the activation of Src and ERK/MAPK signaling.

We would like to thank the reviewer for suggesting this important experiment. We have embedded the PDX#3 tumoroids into collagen in the presence and absence of the β1-integrin blocking antibody AIIB2. Concordant with our conclusion, we observed significantly lower p-Src in the integrin-inhibited samples (manuscript, new Figure 2H and below for your convenience). However, total and p-ERK levels were highly variable between experiments and there was no significant difference between control and AIIB2-treated samples (Figure 1 for reviewer 1 below). We have edited the conclusion in the abstract accordingly: "Collagen–α2β1-integrin interaction activates Src".

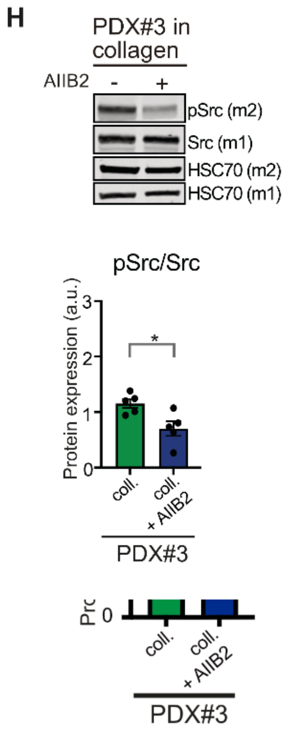

Manuscript FIG2.

(H) Western blot of pSrc, Src, and HSC70 (loading control) in PDX#3-generated tumoroids in collagen ± AIIB2 (1µg/ml), and corresponding quantification of Src phosphorylation ratio (n=5 biological replicates, unpaired t test with Welch's correction, p-value = 0.0230).

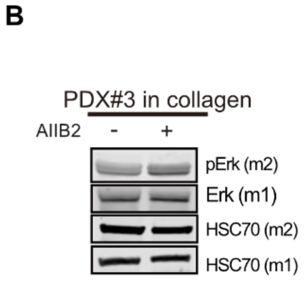

FIG1 for Reviewer#1.
(A) Western blot quantification of Erk phosphorylation ratio in PDX#3 in collagen ± AIIB2 (1µg/ml) (n=6 biological replicates, unpaired t-test, p-value=0.5293).
(B) Corresponding western-blot showing pErk, Erk and HSC70 (loading control) in PDX#3 collagen ± AIIB2.

3. Fig.S2D. It is not obvious that expression of integrin α2 or β1 is altered in response to collagen I culture from these graphs. Please provide quantification.

In the original manuscript, the quantification was provided in Fig 2C. We are sorry that this was not clearly indicated. In the revised version, quantification for Fig S2G is shown in Fig 2C and S2H.

4. Fig. 1C and Video 1 shows that clear formation of lumen-like structures and protrusions could be observed as early as 5 hours post-embedding. It would be important to determine how quickly upregulation of SorLA and maturation of integrin β1 occurs to contribute to the phenotype.

This is indeed an interesting point. Our data show that only PDXs and PDOs with initially high integrin and SorLa levels (PDX#3 vs the others, PDO#2 vs PDO#1) are able to engage the matrix and generate protrusions. Therefore, the initial response is most likely mediated by the already high integrin, and, then, reinforced by SorLA upregulation and integrin maturation in long-term culture.

5. Page6, "Indeed, we detected significantly elevated ERK phosphorylation in PDX#3 tumoroids in collagen (Fig. 3H and S3C) and collagen-induced SorLA upregulation was sensitive to integrin function-blocking

antibodies (Fig. 3I and S3D).” This conclusion could be strengthened by inhibiting ERK and confirming that the ERK pathway is responsible for the upregulation of SorLA mRNA.

This is an excellent point. To explore this, we treated PDX#3 tumoroids with the MEK-inhibitor, trametinib, and analysed SorLA expression and imaged tumoroid polarity. Trametinib treatment significantly reduced ERK phosphorylation and SorLA expression (new Fig. 3J and S3E-F, and below), reduced lumen formation suggesting inhibition of apical-in switching and was concomitant with apparent cell death in PDX#3 tumoroids embedded in collagen (new Fig. 3K, and below).

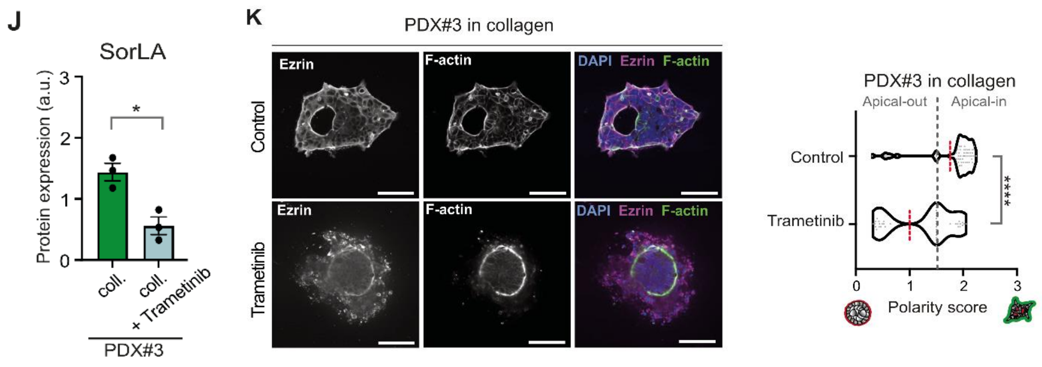

Manuscript FIG3.

(J) Western blot quantification of SorLA expression in PDX#3-generated tumoroids in collagen ± trametinib (250 nM) (n=3 biological replicates, unpaired t test with Welch’s correction, p-value = 0.0122). (K) Representative immunofluorescence images of PDX#3-generated tumoroids in collagen ± trametinib, stained for Ezrin, F-actin and DAPI. Scale bars 10 µm. Corresponding polarity score of PDX#3-generated tumoroids in collagen ± trametinib. Data are displayed as violin plots with mean indicated by a red line. Dashed line (at polarity score 1.5) indicates the mid-point between apical-in and apical-out characteristics [n= 52 (control) and 43 (trametinib); Mann-Whitney test, p-value<0.0001].

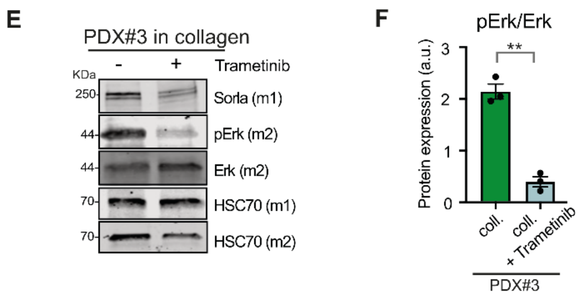

Manuscript FIGS3.

(E, F) Representative western blot of SorLA, β1-integrin, ERK and HSC70 (loading control) and quantification of Erk phosphorylation (pERK/total ERK ratio) (F) in PDX#3 in collagen ± trametinib (250 nM) (n=3 biological replicates, unpaired t test with Welch’s correction, p-value=0.0010).

6. Page 6, “These data suggest the possibility that the PDX#3 tumoroids in collagen switch from RCP-mediated long-loop integrin recycling to a SorLA-driven rapid recycling pathway in a manner that is

dependent on initial matrix-induced integrin signaling." requires additional experimental support. The authors should examine the colocalization of RCP or SorLA with integrin proteins in suspension vs. collagen-embedded organoids to support this conclusion.

We agree with the reviewer that this conclusion was not fully supported by the data. We have unfortunately been unable to obtain high-quality immunofluorescence staining of RCP in the collagen-embedded tumoroids. In response to the criticism, we have edited the text to: "These data suggest the possibility that the polarity switching of PDX#3 tumoroids in collagen is supported by ERK-induced SorLA expression" (page 10).

7. Page 8, "Conversely, HER2/HER3 activation with the HER3 ligand Heregulin- β1 was sufficient to trigger collagen-induced polarity reversion in the thus far matrix agnostic PDX#2 tumoroids (Fig. 5H-I). Upon HER3 stimulation with Heregulin- β1, SorLA was also found to be upregulated in PDX#2 (Fig. 5J and S5E). The PDX#1 tumoroids did not respond, presumably due to their lack of HER3 expression (Fig. 5B and S5C-D)." Since the authors suggest that HER2/HER3 activation or collagen embedding induces polarity reversion by upregulating SorLA, could SorLA overexpression alone trigger collagen-induced polarity reversion in collagen-embedded PDX#1 or PDX#2 organoids?

This is indeed an interesting aspect to explore. We have made numerous attempts to overexpress SorLA in the PDX#2 tumoroids. SORL1-GFP is a very large construct and at the limit of what is feasible for lentivirus particle packing. Albeit we have succeeded in SORL1-GFP transduction in standard breast cancer cell lines in the past (Pietilä et al., 2019 Nat. Communs), we regrettably were unable to achieve the same in these primary PDXs and thus are unable to provide an answer to this interesting question. Furthermore, we would like to emphasize that we have not made any claims in the manuscript that SorLA alone would be sufficient to trigger polarity reversion.

8. Figure 6G does not clearly display apical-in polarity. Please provide representative images that better illustrate the apical-in phenotype.
We thank the reviewer for this point and realise that we have not been sufficiently clear in explaining this experiment.

The tumoroids in this experiment have spread on the 2D peritoneum surface and have thus lost their 3D structure and lumens. To clarify we have added "adhering to **and spreading on** " to the sentence on page 19.

Reviewer #2 (Remarks to the Author):

In this paper, the author investigated the mechanism of polarity control in MUC CRC tumors. The authors found that sensing collagen by integrin α2β1 promotes the expression of SorLA, which in turn promotes integrin recycling. HER2/HER3 upregulates the expression of SorLA. The mechanisms of polarity switching in tumors are of great interest to cell biologists. However, there are concerns that the authors should clarify in this study.

We thank the reviewer for their positive evaluation of our study and for excellent suggestions of how to further strengthen our manuscript.

Major comments

1. This paper consists of two different themes. One is the collagen sensing system in CRC cell clusters. Another is the biological differences between CRC clusters with apical-in and apical-out polarity.

In the collagen-sensing system part, the authors found that integrin α2β1 promotes SorLA expression, contributing to integrin rearrangement. In characterizing apical-in and apical-out polarity, the authors found differences in collagen fiber rearrangements and HER2/HER3 signaling. Both are interesting results but should be studied more deeply as independent studies, as they have very different content. The earlier subject is the sensing and execution of polarity switching, while the later subject is the consequence of polarity switching. It is difficult to summarize as an 'axis'.

We agree that our study consists of both mechanistic investigation of the mechano-chemical pathways involved in collagen sensing and regulation of the apical-in vs. apical-out polarity as well as functional studies on the biological relevance of CRC tumoroid polarity. However, to us these two aspects are closely linked. We uncover here how integrin-mediated collagen binding in PDX#3 (and new data with additional PDO model#2) triggers polarity switching and how signaling pathways involving Src, ERK and HER2/HER3 are necessary to maintain apical-in polarity. Based on these data we explore the functional outcome of inhibiting these pathways in vitro (and new data in vivo) as well as their correlation with polarity phenotypes in clinical patient samples. All together our study uncovers molecular details that correlate with therapy responses in vivo (our new data) and may be predictive of CRC behavior and invasive potential in the clinical setting.

2. It is an excellent discovery that integrin α2β1 is a sensor of collagen for polarity switching from apical-out to apical-in in PDX#3 tumoroids, but it may be specific to this case with exceptionally high β1 integrin levels. Indeed, it is a good model for studying the sensing system but the universality of the findings is not convincingly demonstrated.

This is a valid criticism. To begin to address this, we have obtained two additional CRC models (see response to point 5 below). One of these patient-derived organoid lines (PDO#1) resembles PDX#1 and #2 in maintaining apical-out polarity in collagen and the other one (PDO#2) is similar to PDX#3 and reverts to apical-in in collagen. Supporting our observation with the PDX models, we found that β1-integrin level (both immature and mature forms) is significantly higher in PDO#2 compared to PDO#1 (Figure S2C, and below for your convenience). Together with the other pieces of data using the new PDO models, this finding suggests that the newly described polarity regulator pathway involving β1 integrin, HER2/HER3 and SorLA may be a common mechanism in CRC.

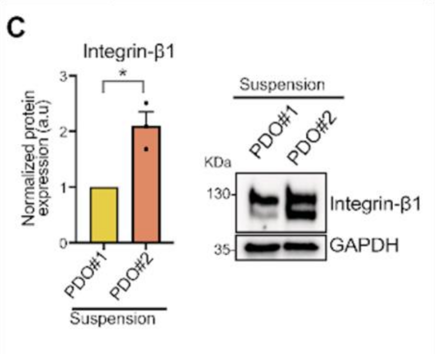

Manuscript FIGS2

(C) Western blot analysis and corresponding blot of integrin-β1 in PDO#1- and PDO#2-generated spheres in suspension after 24h, relative to PDO#1 (n=3 biological replicates, ratio paired t test, p=0.0245).

Individual comments

3. The scoring system for quantifying polarity status should be described in detail in the Materials and Methods section, or it should be cited from a previous publication. According to the results section, the authors used Ezrin to calculate the polarity score. In this paper, either F-actin or Ezrin, or both, are used as indicators of the apical membrane. However, in many images, especially Figure 5H, the localization of F-actin and Ezrin is not the same. Please discuss the discrepancy of the staining of F-actin and Ezrin, and also show the score by F-actin.

Thank you for this important comment. We have included a better description of the polarity score in the methods and copied it here: The computation of the polarity score used throughout the manuscript is based on three parameters, defining three sub-scores: Sub-score 1) The presence/absence of lumen, set to 0 in case of lumen absence, or 1 in case of the presence of one or more lumen. Sub-score 2) Circularity, obtained through the shape descriptor function on ImageJ. A value of 0 would be obtained for a perfect sphere, whereas a value closer to 1 translates to a higher number of protrusions. The segmentation here is based on the actin fluorescence signal. Sub-score 3) The apicobasal ezrin signal ratio, which is the ratio of the ezrin luminal signal (or the mean of luminal signals in case of multiple lumen) to the total ezrin fluorescence (luminal+cortical). This value varies from 0 (complete cortical signal) to 1 (complete luminal signal). The sum of these three sub-scores is the total polarity score (PS) varying from 0 (perfect apical-out) to 3 (perfect apical-in). In case of non-polarized structures (cytoplasmic ezrin signal), we set the polarity score to 1.5 per default.

4. The polarity score for the PDX#3 tumoroids embedded in collagen was mostly 2. In most of the control images of PDX#3 tumoroids in collagen, F-actin and Ezrin were detected in both the apical and inner membranes. Do the images represent the score? It would be better to show a score in each image.
The polarity scores are calculated on multiple spheroids (minimum 20 per experiment), therefore there is an inherent variability due to the inevitable mix of polarity phenotypes within a model depending on the experimental condition (suspension, collagen, drugs). More than the absolute value of PS, we focus here on the PS variation across models or conditions to determine the impact of culture conditions or the presence of drugs on the polarity phenotype. We have provided some examples below to clarify the link between the scores and the images.

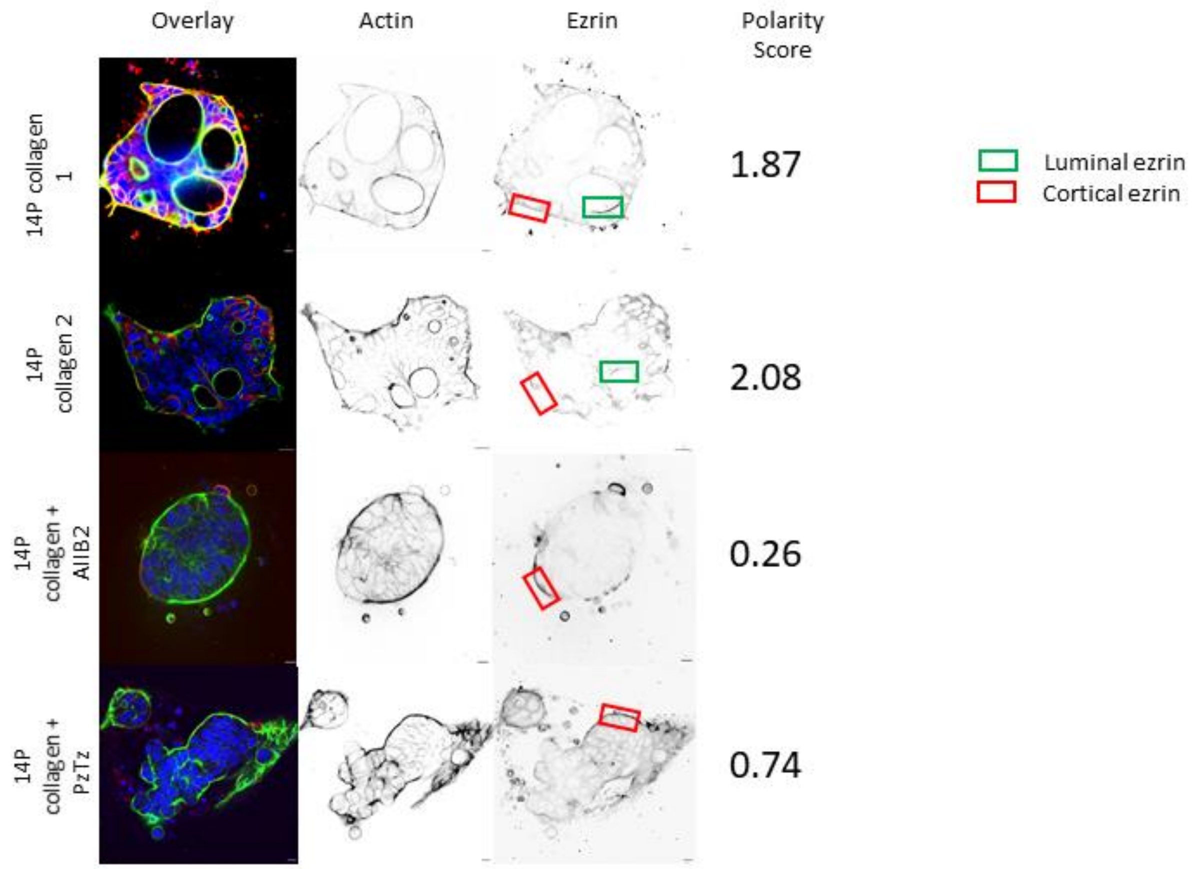

FIG1 for Reviewer#2. Example images of control and integrin antibody-treated tumoroids embedded in collagen, and stained as indicated. The red zones indicate cortical ezrin and green zones luminal ezrin. Corresponding polarity scores are provided on the right.

5. Only three CRC PDX lines (2 polarity un-switchable, 1 polarity switchable) were used in this study. Due to the small number of samples, it is extremely important to validate the findings. Multiple organoid lines should be investigated for validation. It may be difficult to obtain additional PDX lines, but please explain why this study should be conducted with PDX tumoroids or PDOs rather than with more readily available CRC organoid lines.

We agree with this point. We have collected additional samples from patient-derived CRC tumors, and isolated patient-derived organoids (PDO) that behave either like PDX1 and PDX2 (CRC0089; referred to in the revised MS as PDO#1), or like PDX3 (CGR0116, referred to in the revised MS as PDO#2) upon interaction with collagen (Fig. S2A and S2B in the manuscript and below for your convenience). We feel that these additional clinically-relevant models provide important further validation of our data. We have also explored the possibility of using classical CRC cell lines. In supplementary Fig. S4 we had already provided data that the CRC cell line LS513 resembles PDX#3 in being apical-out in suspension and apical-in in collagen. In addition, we have observed that HT29 cells do revert the localisation of integrins and form focal adhesions in collagen, suggesting an apical-in phenotype (not shown). However, we have not been able to identify cell lines that would be matrix agnostic like PDX#1 and #2 and the new model PDO#1, limiting our ability to conduct studies also with cell lines.

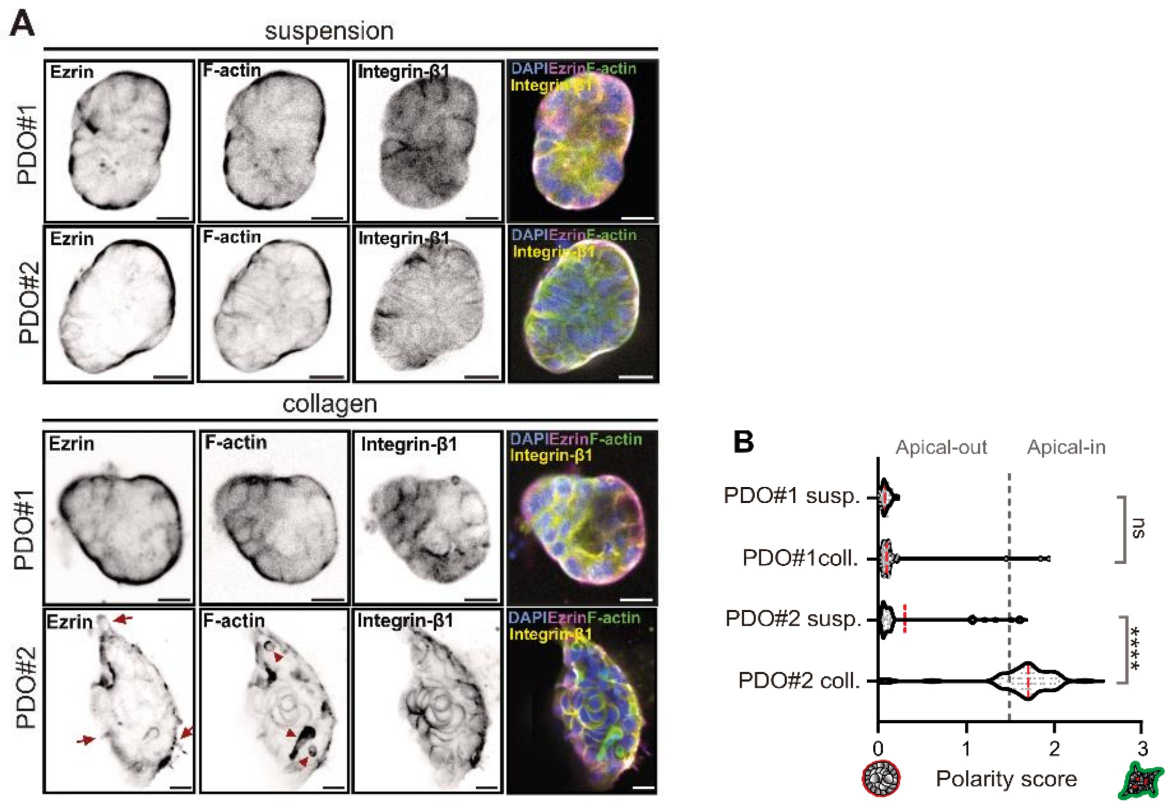

Manuscript FIGS2

(A) Representative immunofluorescence images of PDO#1- and PDO#2-generated spheres in suspension and in collagen, showing Ezrin, F-actin, β1-integrin and DAPI. In the PDO#2 collagen condition, arrows indicate protrusions and arrow heads indicate lumens. Scale bars 20 µm. (B) Polarity score of PDO#1- and PDO#2-generated spheres before and after inclusion in collagen for 3 days. Data are displayed as violin plots with mean indicated by a red line. Dashed line (at polarity score 1.5) indicates the mid-point between apical-in and apical-out characteristics. Mann-Whitney test for PDO#1: p value=0.107; n=55 (suspension); 59 (collagen) from four biological replicates, and for PDO#2: p value<0.0001; n=51 (suspension); 53 (collagen) from three biological replicates.

6. To show that 'SorLA-dependent Integrin-β1 trafficking is induced by HER2/HER3 signaling', it should be shown that inhibiting HER2/3 suppresses the expression of SorLA and integrin β1 in PDX#3. In addition, it should be shown whether HRG induces integrin β1 in PDX#2.

This is a valid point. We tested this by treating PDX#3 with trastuzumab/pertuzumab and analysed SorLA and integrin β1 expression. SorLA expression was significantly downregulated upon HER2/HER3 inhibition in PDX#3 (new fig 5C and S5E, and below for your convenience). These data are in line with HER2/HER3 inhibition blocking apical-in polarity reversion of PDX#3 in collagen. However, it is important to distinguish between increased integrin expression and increased receptor trafficking. Our data indicate that SorLA silencing attenuates β1-integrin recycling to the plasma membrane and apical-in polarity (Fig. 4C-D). However, there are no significant changes in integrin β1 expression. In line with this, we do not observe significant changes in integrin β1 when treating PDX#3 with HER2/HER3 inhibiting trastuzumab/pertuzumab or PDX#2 with HRG (figure 2 for reviewer#2 below)

C

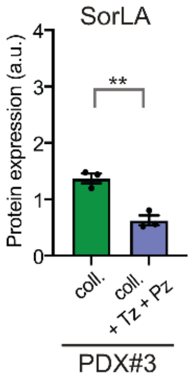

Manuscript FIG5.

(C) Western blot quantification of SorLA expression in PDX#3-generated tumoroids in collagen ± a combination of trastuzumab and pertuzumab (Tz + Pz; both 10 µg/ml) (n=3 biological replicates, unpaired t test with Welch's correction, p-value=0.0039).

E

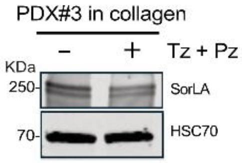

Manuscript FIG S5.

(E) Western blot showing SorLA and HSC70 (loading control) in PDX#3 in collagen ± a combination of trastuzumab and pertuzumab (Tz + Pz; both 10 µg/ml).

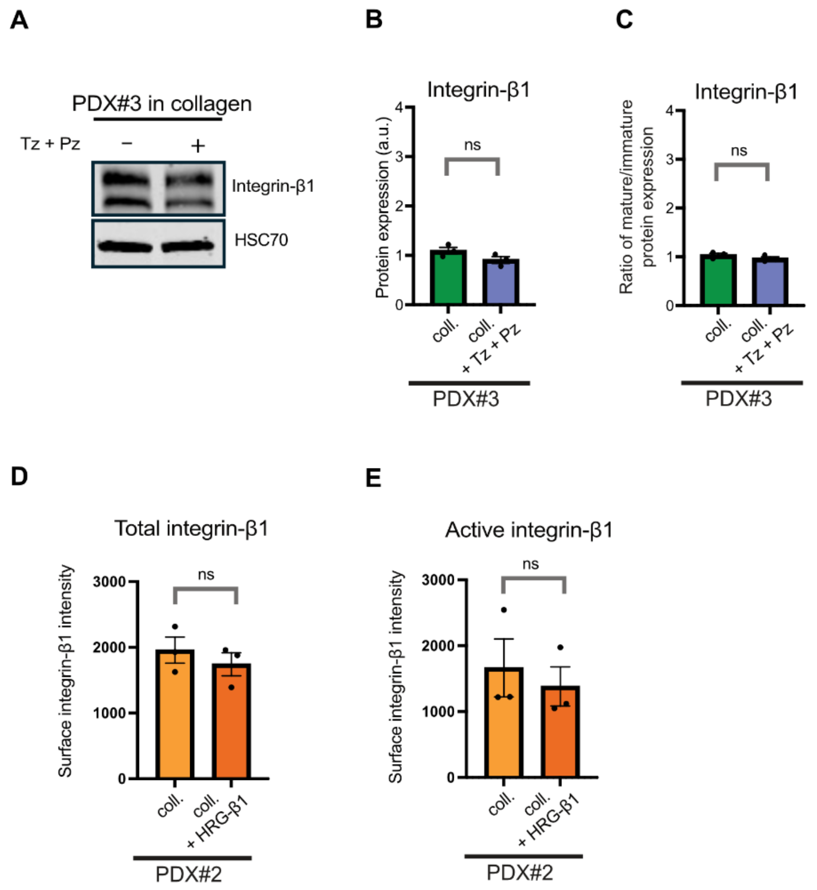

FIG2 for Reviewer #2.

(A) Western blot of SorLA, β1-integrin and HSC70 (loading control) in PDX#3 in collagen ± a combination of trastuzumab and pertuzumab (Tz + Pz; both 10 µg/ml). (B and C) Quantifications of total (B) and maturation ratio of (C) β1-integrin in PDX#3 in collagen ± a combination of trastuzumab and pertuzumab (Tz + Pz; both 10 µg/ml) (n=3 biological replicates, unpaired t-test, p-value$_{\text{total β1-integrin}}$= 0.1298, p-value$_{\text{maturation ratioβ1- integrin}}$= 0.2385).

(D and E) Flow cytometry analysis of cell-surface total (P5D2-488) (D) and active (12G10-647) β1-integrin (E) geometric mean signal intensities in PDX#2-generated organoids in collagen ± Heregulin-β1 (Hrg-β1, 20 ng/ml). n=3 biological replicates, unpaired t-test, p-value$_{\text{total β1-integrin}}$= 0.4665, p-value$_{\text{active β1-integrin}}$= 0.6246.

7. For evaluating the interaction between CRC tumoroids and the peritoneum, the model here (Fig. 6) is not suitable. In the process of epithelial cells becoming malignant, invasion into the basement membrane is an important process. On the other hand, peritoneal invasion of colorectal cancer is a completely different phenomenon. Cancer cells first come into contact with peritoneal cells, and then invade the basal membrane after pushing them away. Therefore, the model should be peritoneal cells, not the basement membrane.

The exact mechanism by which peritoneal invasion by CRC clusters occurs remains poorly understood.

Nevertheless, histological studies have helped to draw conclusions on the pattern of invasion of peritoneal metastasis from ovarian and colorectal cancers. The typical pattern of peritoneum invasion for ovarian cancer cells after adhesion and breaching of the mesothelial layer is the formation of numerous small clusters that remain confined to the peritoneal surface (PMID: 29164988). On the contrary, CRC peritoneal metastases, while also starting with superficial implantation, are characterized by a greater tendency for deeper invasion into the sub-mesothelial tissue associated with advanced stromal remodelling (PMID: 27678351). Interestingly, this difference in invasion pattern renders CRC surgical removal much more difficult to achieve than in the case of ovarian cancer cells.

Also, the thin layer of mesothelial cells is very often degraded during laparoscopic CRC surgery, due to pneumoperitoneum (air injected into the peritoneal cavity) (PMID: 10347302), or altered by chemotherapeutic drugs used for CRC treatment (PMID: 34916522,PMID: 39948828), leaving the peritoneum basement membrane directly exposed to tumor cell clusters. Finally, at the invasive front of primary mucinous CRC, clusters of CRC cells are often observed as being detached from the primary tumors. From here, they are thought to cross the conjunctive tissue, the muscle, and the serosa before reaching the peritoneal cavity (Zajac et al, 2018).

For all these reasons, we believe that CRC cell clusters become exposed to decellularized peritoneum ECM early on during the primary invasion process (arrival to the peritoneum cavity) or during the metastatic invasion of a secondary peritoneal site (leaving the peritoneum cavity), supporting our use of this model.

8. It should be shown that SorLA-silenced or HER2/3-inhibited PDX#3 show apical-out phenotype in vivo.

We thank the reviewer for this excellent suggestion that has enabled us to significantly strengthen the manuscript and extend our in vitro treatment models to the in vivo setting. We have treated PDX#3 xenograft bearing mice with control antibody or the combination of trastuzumab and pertuzumab for 3 weeks. The tumors were resected and submitted for immunohistochemistry analysis. HER2/HER3 inhibition resulted in a significant loss of polarized apical-in structures (based on H&E, alcian blue and pan-CK IHC and ezrin immunofluorescence staining). In addition, the treatment induced marked cell death in the tumors (Fig. 5G-K, supplementary figure S6 and below).

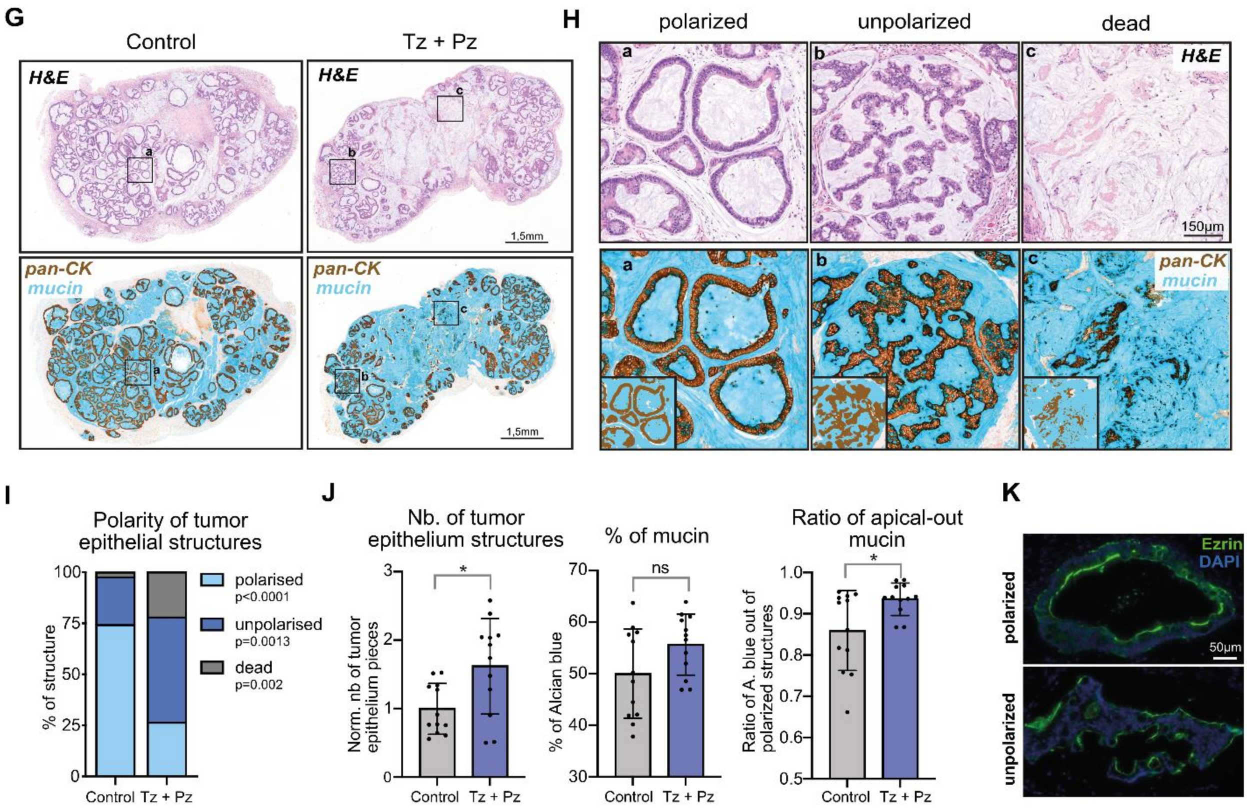

Manuscript FIG5

(G-H) Representative images (G) and zoomed-in insets (H) of Hematoxylin & Eosin (H&E), Alcian Blue and Pan-Cytokeratin (Pan-CK) stainings in PDX#3 tumors extracted from the flank of control vs treated (Tz+Pz, 10 mg/kg) mice. Scale bar, 1.5mm. Insets illustrate what was characterized as polarized, unpolarized and dead tumor epithelial structures based on the stainings. Insets on the Alcian blue images show the segmentation results used in quantitative analysis (See FigS6B). Scale bar 150 µm. (I) Qualitative classification of polarized, unpolarized and dead tumor epithelial structures in control and treated tumors shown as a percentage of the whole tumor. Statistical analysis was performed between the conditions for each indicated feature, p values shown in the graph (n=12, Welch's t test). (J) Segmentation-based quantification of the number of epithelial structures (Pan-CK) (p value=0.0139), percentage of mucin (Alcian blue) (p value=0.0771) and the ratio of apical-out mucin (p value=0.022) (mucin outside the closed tumor epithelial structures) comparing control and treated tumors (n=12). Mean +/- SD are shown as bar graphs with single data points displayed as black circles. Unpaired t-test with Welch's correction statistical test. (K) Representative images of polarized and unpolarized tumor epithelial structures based on ezrin immunofluorescence staining. Scale bar, 50µm.

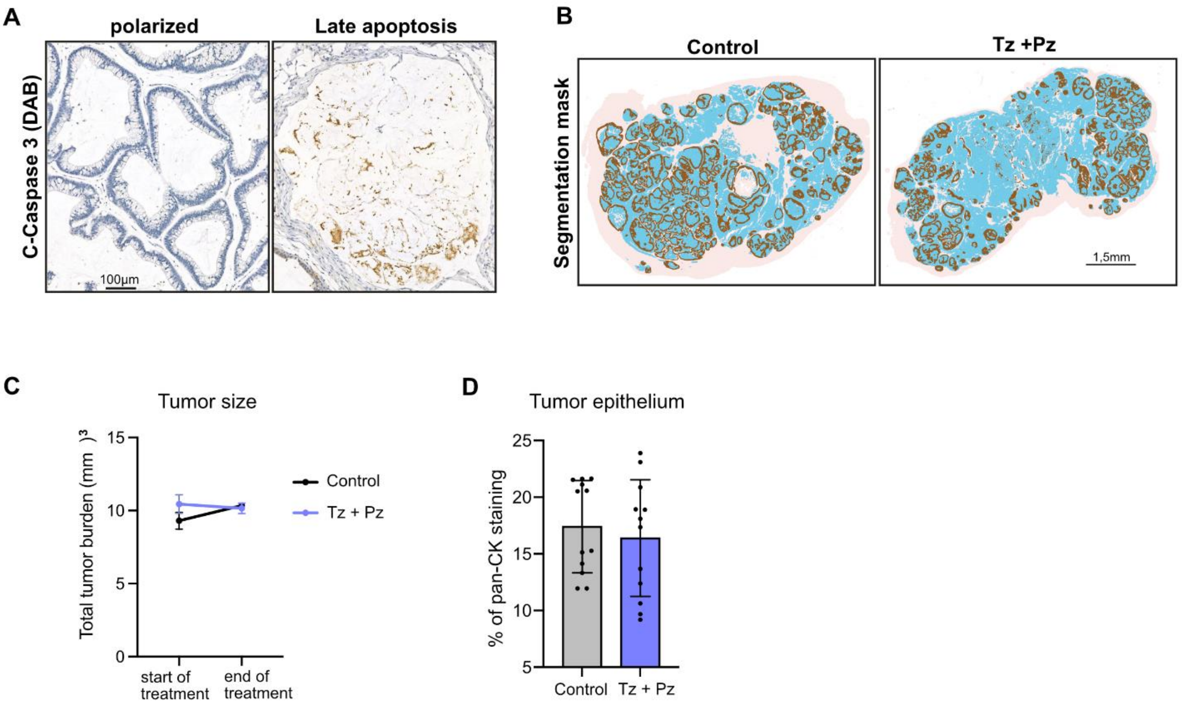

Manuscript FIGS6

(A) Representative images of tumor epithelial structures characterized as polarized (left) and dead (right), stained with cleaved caspase-3 antibody to show late apoptotic traces in the “dead” class. (B) Segmentation mask obtained from the representative images shown in Fig.2H. (C) Total tumor burden in control and trastuzumab and pertuzumab combination (Tz + Pz) treated mice. Average of total tumor size from both flanks is plotted (n=2 mice /group). (D) Quantification of the percentage of tumor epithelial structures (Pan-Cytokeratin mask) over the total size of the sample cut, comparing control and treated tumors (n=12). Mean +/- SD are shown as bar graphs with single data points displayed as black circles. Mann-Whittney statistical test, non-significant.

9. Overall, the discussion is not well written. Discussion should not be a repetition of results. Some of the contents of the Discussion have already been described in the results section.

Thank you for this comment. We have now significantly edited the discussion by reducing the reiteration of the results and including new discussion points.

10. Please define the terms ‘tumoroid’, ‘spheres’, and ‘PDO’ in the text or Materials and Methods section.

These terms have been defined in the methods on pages 25-26.

<u>Sphere</u>: Cluster of tumor cells.

<u>Tumor Sphere with Inverted Polarity (TSIP)</u>: specific cluster of tumor cells showing enrichment of apical domain markers at its peripheral surface and basolateral markers on its internal cell-cell contacts.

Tumoroid: Small Pieces of patient-derived xenograft (PDX) tumors maintained no more than 3 days in culture

Organoid: Cell line of tumor cells maintained as spheres

Patient-Derived Xenograft Organoid (PDXO): PDX-derived cell line maintained as spheres

Patient-Derived Organoid (PDO): Cell line derived from freshly collected patient tumors and maintained as cell clusters

11. Introduction: 'the TGF-β pathway has been implicated (Okuyama et al., 2016, Onuma et al., 2021, Canet-Jourdan et al., 2022).' Canet-Jourdan et al. argued that the TGF-β pathway is involved in polarity switching and that Rho is downstream of this, but Okuyama and Onuma et al. do not mention TGF-β, and instead clarified that Rho is involved in polarity switching.

Edited accordingly on p 2 "although the TGF-β and Src pathway have been implicated (Okuyama et al., 2016, Onuma et al., 2021, Canet-Jourdan et al., 2022)."

12. Materials and methods: Generation of tumoroids. They cited Sato et al, 2011, but there is no description of tumoroid preparation in the reference. Rather, it seems to have been first described in doi/10.1073/pnas.1015938108.

We apologize for this mistake and have corrected it with the proper reference (https://doi.org/10.1242/jcs.259256) in the text.

13. P4 line 9: What does 'dynamic contact' mean?
We apologize for this unclear terminology and have changed this to transient interactions.

14. Bright field images in Figure 1C and Figure S2E are too dark to see the details.
These have been adjusted.

15. P5 line 3: 'In PDX#3 tumoroids, the localization of β1-integrins (detected with the P5D2 antibody) shifted from lateral (cell-cell) to basal (cell-ECM)' is not true. The staining is clearly seen in basal in suspension.

We have edited this on page 6 "In PDX#3 tumoroids, the localization of β1-integrins (detected with the P5D2 antibody) shifted from both basal and lateral (cell-ECM and cell-cell) to basal…"

16. Figure 2A: The pseudo color for 12G10 should not be yellow because the colocalized staining of red (P5D2) and green (F-actin) will be also yellow.
We apologize if the labelling of the colours was unclear. P5D2 in the figure is in magenta and the resulting combination is white.

17. The change of staining pattern of β1 integrin should be shown in all the latter results.
We appreciate this comment and realise in hindsight that inclusion of integrin staining would have been a good idea. Unfortunately, it is not feasible (or ethically in line with the 3R principle) to redo all the experiments to obtain the integrin staining. However, to address this important point we have included β1-integrin staining in the new experiments with the two additional PDO models in suspension, in collagen and with the treatments.

18. Figures 2F and 4F should be removed because some of the data for each scheme is not displayed in the respective figures. This is related to the logical problem, syllogisms, in this manuscript. Contents of the scheme, if any, should be restricted to the contents of the figure.
We agree and have removed these.

19. P3 last line: ‘66% of patient-derived TSIPs retain their apico-basolateral polarity’. Does this mean ‘66% of patient-derived TSIPs derived from MUC CRC patients retain their apico-basolateral polarity’?

We apologize for the lack of clarity regarding this sentence. We replaced it in the manuscript with “66% of MUC CRC patients’ peritoneal effusions contained TSIPs that retained their inverted polarity when transferred to collagen gels (cf Fig 1B of Canet-Jourdan et al, 2022: ‘*Quantification of TSIPs polarity in suspension (from peritoneal effusions) and after 3 to 6 days in collagen-I gels’).*

20. P7 second paragraph: Please explain what are ‘the technical challenges associated with performing cell biology with PDX models’ in detail. Are you referring to the ‘cell-surface biotinylation-based integrin uptake assay’? If so, please explain why it cannot be performed with PDOs.
Yes indeed. These experiments require a lot of cells and the ability to efficiently downregulate SorLA with RNAi transfection. This we were able to achieve with the LS513 cell line but not with the primary PDX-derived tumoroids or the PDOs. However, since the LS513 cell line reverts in collagen similarly to PDX#3, we are confident that this is a valid experimental model system for these biochemical experiments.

21. P7 second paragraph: ‘secrete’ is not an appropriate verb here. ‘discharge’?
Indeed, thank you for pointing this out. We have replaced the word ‘secrete’ with ‘release’

22. The last paragraph is not an appropriate conclusion in this section.
We have removed this conclusion and the related figure 4F.

23. Figure 5B: In the Western blotting shown, both HER2 and HER3 are elevated in PDX#2. The quantitative analysis states that there is no significant difference in HER3, but as the number of trials is small and the values fluctuate greatly, it is difficult to draw conclusions based on the significance of this experiment alone. It would be better to discuss the possibility that factors other than HER2/HER3 are involved in this phenomenon.
This is a valid point. We have moved these data to the supplement Fig. S5 and edited the results section to tone down the emphasis on the significance of the data. “Although HER2 is significantly upregulated in both PDX#2 and PDX#3 in collagen, HER3 induction was more variable in PDX#2 and reached significance only in the collagen-responsive PDX#3 (Fig. 5A-B)” (page 13). Furthermore, we have included the following sentence “However, we cannot exclude the possibility of additional factors contributing to this response. “ (page 16).

24. Figure 5G: The difference is marginal and there is a synergistic effect when Tz and Pz are used together. From these results, it is difficult to say ‘indicating a functional role for HER2/HER3 signaling, but not HER2/HER2 homodimers’.
We have removed the sentence: ‘but not HER2/HER2 homodimers’.

25. Figure 5J, 7D, 7E: There are strange shapes in the graph.
We apologize for this and have edited the graphs

26. P10 first paragraph is not an appropriate conclusion in this section.
edited

27. Fig.7A: Staining of apical markers should be shown.
We agree that inclusion of ezrin staining would have been valuable. However, we have been unable to obtain more samples from these clinical specimens and have therefore been unable to carry out new stainings. However, we have included ezrin staining in our new in vivo data showing that HER2/HER3 inhibition regulate polarity in the PDX#3 tumors (Figure 5K)..

Reviewer #3 (Remarks to the Author):

Pasquier and colleagues present an intriguing model aimed at explaining polarity dynamics in mucinous colorectal adenocarcinoma. Overall, the experiments seem to be well-conducted, although it is important to note that the majority of observations supporting the main conclusions of the paper stem from a single patient-derived xenograft (PDX) model that aligns with the authors' proposed model. I have several questions regarding the screening of different integrins in the PDX models and the validation of their findings in human samples:

We would like to thank the reviewer for their careful and insightful evaluation of our work.

1. In relation to the data presented in Figure S2, which positive control was included to substantiate the claim that several immunodetections for the various integrins are indeed negative?

We apologise for the colour coding (based in linear values) of the panel leaving the reader with the impression that most integrins are not expressed at all. We have modified it as shown below (and in FigS2D). Logarithmic values are used in order to more carefully display the expression of other integrins.

D

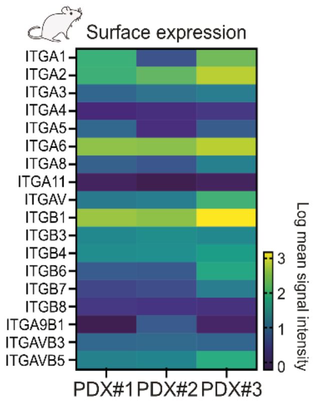

Manuscript FIGS2

(D) A heatmap representing logarithmic mean cell-surface expression of the indicated integrins in PDX#1, PDX#2 and PDX#3 tumors dissected from mice.

2. Concerning the data shown in Figure 7 and Figure S7 for human tumors, I do not recognize the histology as mucinous, particularly in the apical-in image. Could the authors provide images that include larger areas of tissue to clearly demonstrate that these tumors are indeed mucinous? Additionally, it would be helpful to see the original IHC images without normalization.

Thank you for this very valid comment. Please find below (Fig1 for reviewer#3) larger areas of the original images in grey scale (these are not traditional IHC but rather immunofluorescence performed on FFPE) where mucinous areas are highlighted in yellow.

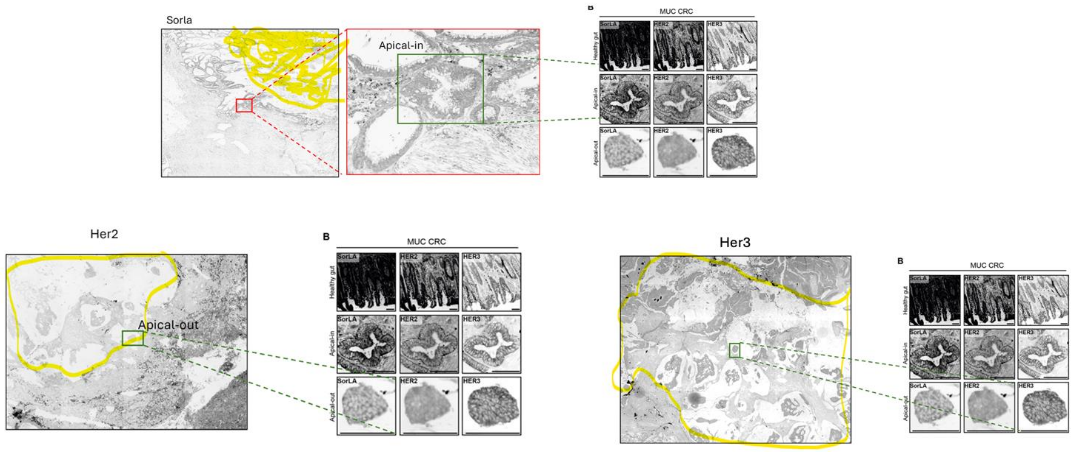

Fig 1 for Reviewer#3.

Immunofluorescence staining of SorLa, HER2 and HER3 on FFPE MUC CRC patient samples. Larger areas shown to indicate the mucinous histology of tumors (highlighted in yellow).

3. If I am correct, HER2 or HER3 expression should not be elevated in normal colonic tissue, and such expression is not commonly observed in colon adenocarcinoma. The authors should address this issue.

To the best of our understanding from reviewing the literature and publicly available data, both are expressed in normal colon and in CRC. HER2 amplification is relatively rare in CRC but has been reported, and also in mucinous CRC. The FDA has recently approved HER2-targeted therapies for HER2-positive CRC, demonstrating both functional and clinical relevance for high HER2 expression in a subset of patients. Some publicly available data addressing these points are collected below.

HER2 in normal colon

Data from Human Protein Atlas: https://www.proteinatlas.org
Conclusion: HER2 is present in colon at protein and mRNA level. IHC levels "low" in colon, mRNA (RNA-seq) levels "moderate-to-high".

Mean HER2 mRNA and protein expression in GI tract vs other organs

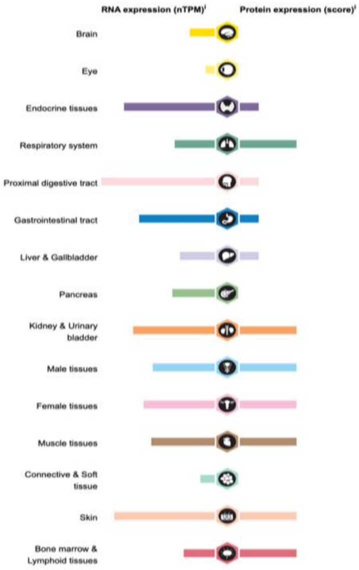

Representative HER2 IHC of human colon

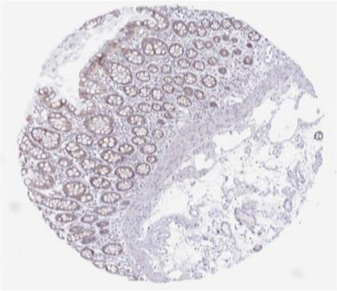

HER3 in normal colon

Data from Human Protein Atlas: https://www.proteinatlas.org
Conclusion: HER3 is present in colon at protein and mRNA level. IHC levels "high", mRNA levels "moderate-to-high".

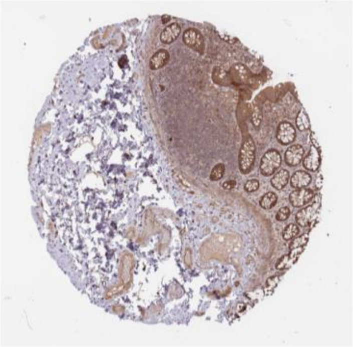

Mean HER3 mRNA and protein expression in GI tract vs other organs

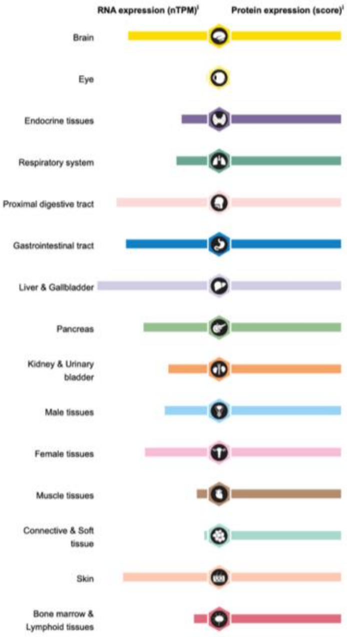

Representative HER3 IHC of human colon

HER2 in mucinous CRC

Conclusion: Both HER2 amplification (ref 1) and protein expression in the absence of amplification (low expression)
(refs 2-4) have been reported in subsets of patients with mucinous CRC.

1) Wang et al., 2022. Oncologist. 2022 Mar 4;27(2):104-109
https://pubmed.ncbi.nlm.nih.gov/35641204/

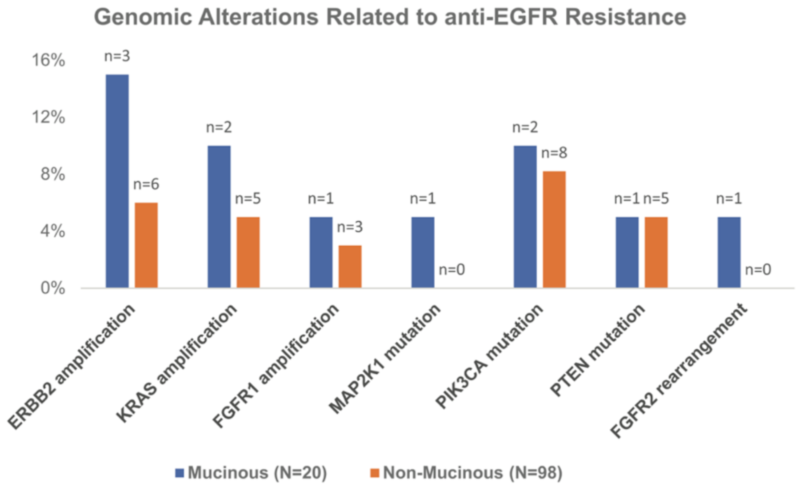

2) Hashimoto et al., 2023. Br J Cancer. 2023 Oct;129(7):1176-1183
https://pubmed.ncbi.nlm.nih.gov/37543670/

Table 1. Clinicopathological and molecular features of *HER2*-amplified, HER2-low, and HER2-negative colorectal carcinoma.

	All patients (*N* = 1009)	*HER2*-amplified (*n* = 25)	HER2-low (*n* = 46)	HER2-negative (*n* = 938)	Amp vs. neg	Low vs. neg
Age					0.93	0.40
Median	64	66	60	64		
Range	17–92	37–83	38–87	17–92		
Sex					0.74	0.15
Male	608 (60%)	16 (64%)	23 (50%)	569 (61%)		
Female	401 (40%)	9 (36%)	23 (50%)	369 (39%)		
Location					0.18[a]	0.024[a]
Right colon	287 (28%)	4 (16%)	20 (43%)	263 (28%)		
Left colon	423 (42%)	14 (56%)	18 (39%)	391 (42%)		
Rectum	299 (30%)	7 (28%)	8 (17%)	284 (30%)		
Stage					0.23[b]	0.74[b]
I	95 (9%)	2 (8%)	2 (4%)	91 (10%)		
II	387 (38%)	7 (28%)	19 (41%)	361 (39%)		
III	323 (32%)	13 (52%)	19 (41%)	291 (31%)		
IV	204 (20%)	3 (12%)	6 (13%)	195 (21%)		
Tumour grade					0.87	0.13
Low	964 (96%)	24 (96%)	46 (100%)	894 (95%)		
High	45 (4%)	1 (4%)	0	44 (5%)		
Mucinous component					0.043	0.84
<10%	871 (86%)	25 (100%)	40 (87%)	806 (86%)		
≥10%	138 (14%)	0	6 (13%)	132 (14%)		

Conclusion. High HER3 expression is seen in about 80% of primary CRC, corresponding lymph node metastases and liver metastases. There is a correlation between HER3 expression in primary tumour and metastases in CRC.

3) Shabbir et al., 2016. BMC Cancer. 2016 Nov 7;16(1):855
https://pubmed.ncbi.nlm.nih.gov/27821098/

			Intensity of Her-2 staining *n*(%)				*p*-value
			Negative	Weak	Mod	Strong	
Gender	F	44	7 (15.9)	9 (20.5)	20 (45.5)	8 (18.2)	0.353*
	M	51	13 (25.5)	10 (19,6)	15 (29.4)	13 (25.5)	
Age	≤60 year	70	15 (21.4)	12 (17.1)	25 (35.7)	18 (25.7)	0.438*
	>60 year	25	5 (20.0)	8 (32.0)	8 (32.0)	4 (16.0)	
Grade	I	14	3 (21.4)	4 (28.6)	4 (28.6)	3 (21.4)	0.514*
	II	59	11 (18.6)	9 (15.3)	23 (39.0)	16 (27.1)	
	III	22	6 (27.3)	6 (27.3)	8 (36.4)	2 (9.1)	
Type	Mucinous	13	5 (38.5)	3 (23.1)	4 (30.8)	1 (7.7)	0.294**
	Nonmucinous	82	15 (18.3)	16 (19.5)	31 (37.8)	20 (24.4)	

4) Shin & Sung 2014. Gut Liver. 2014 Jan;8(1):13-23
https://pubmed.ncbi.nlm.nih.gov/24516696/

Factor	Cathepsin D		p-value	p53		p-value	COX-2		p-value	EGFR		p-value	C-erbB-2		p-value
	Positive (n=103)	Negative (n=163)		Positive (n=162)	Negative (n=104)		Positive (n=100)	Negative (n=166)		Positive (n=95)	Negative (n=171)		Positive (n=80)	Negative (n=186)	
Sex			0.788			0.711			0.826			0.823			0.563
Male	63 (61.2)	97 (59.5)		96 (59.3)	64 (61.5)		61 (61.0)	99 (59.6)		58 (61.1)	102 (59.6)		46 (57.5)	114 (61.3)	
Female	40 (38.8)	66 (40.5)		66 (40.7)	40 (38.5)		39 (39.0)	67 (40.4)		37 (38.9)	69 (40.4)		34 (42.5)	72 (38.7)	
Diagnosis			0.037			0.855			0.038			0.258			0.634
Right side	39 (37.9)	42 (25.8)		50 (30.9)	31 (29.8)		38 (38.0)	43 (25.9)		33 (34.7)	48 (28.1)		26 (32.5)	55 (29.6)	
Left side	64 (62.1)	121 (74.2)		112 (69.1)	73 (70.2)		62 (62.0)	123 (74.1)		62 (65.3)	123 (71.9)		54 (67.5)	131 (70.4)	
Histologic type			0.984			0.225			0.215			0.390			0.760
Nonmucinous	98 (95.1)	155 (95.1)		152 (93.8)	101 (97.1)		93 (93.0)	160 (96.4)		92 (96.8)	161 (94.2)		77 (96.2)	176 (94.6)	
Mucinous	5 (4.9)	8 (4.9)		10 (6.2)	3 (2.9)		7 (7.0)	6 (3.6)		3 (3.2)	10 (5.8)		3 (3.8)	10 (5.4)	

HER3 in mucinous CRC

Conclusion: HER3 is present in LS174T mucinous CRC cell line in vitro (ref 5). No IHC published specifically for mucinous CRC in PubMed. However, HER3 protein is present in high percentage of unclassified CRCs, probably including mucinous subtype (ref 6).

5) Kuracha et al., 2023. Int J Mol Sci. 2023 Aug 2;24(15):12331
https://pubmed.ncbi.nlm.nih.gov/37569713/

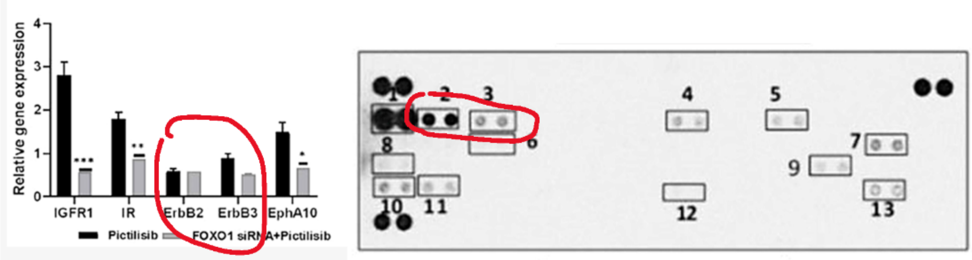

6) Ledel et al., 2015. Acta Oncol. 2015 Apr;54(4):480-6
https://pubmed.ncbi.nlm.nih.gov/25601452/

Furthermore, we have analyzed the level of HER2, HER3 and SORLA using immunohistochemistry on a new cohort containing 3 healthy colon and 20 CRC samples The three proteins are expressed both in non-cancerous and cancerous colon tissues. Although HER2 expression is weaker, some moderately expressing CRC are observed (Fig. for reviewer 3).

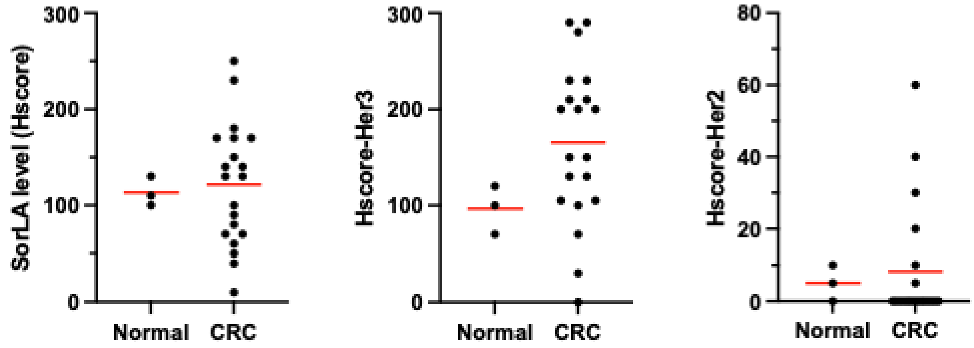

Figure 2 for reviewer #3: Level of expression (determined by the h-score) of SorLA, HER3 and HER2 in normal (n=3) vs cancerous colon (CRC, n=20) samples.

4. While the authors have validated their model using 25 mucinous adenocarcinomas, it would be logical to also include non-mucinous tumors for comparison.

This is a valid point. Unfortunately we did not have the possibility to include a similarly-sized cohort of non-mucinous tumors in our manuscript. Nevertheless, we were able to test HER2, HER3 and SorLA expression in 10 non-mucinous samples and compare their level with 10 mucinous adenocarcinomas. We see that their expression is globally similar in mucinous vs non-mucinous CRC (Figure 3 for Reviewer#3).

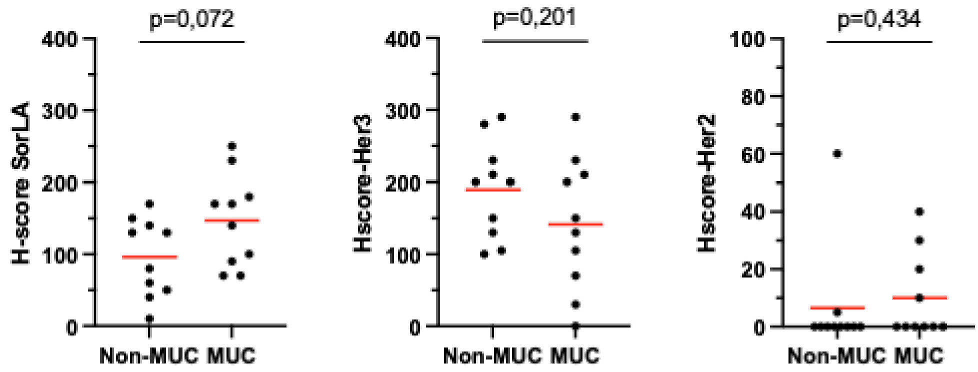

Fig 3 for Reviewer#3. Expression of HER2, HER3 and SorLA in a new cohort of 10 MUC CRC vs 10 Non-Mucinous CRCs.

In addition, we have now included in the revised manuscript data from an additional patient-derived-organoid line (CGR0116; PDO#2 in the manuscript), which is not derived from mucinous CRC. Yet, PDO#2 shares the same collagen-induced response as the mucinous PDX#3, suggesting the mechanism of polarity reversion we propose applies also to non-mucinous tumor spheres.

Reviewer #4 (Remarks to the Author):

Mucinous CRC tumors are in general a poor-prognosis tumor subtype because of many different reasons. In this study, the authors focus on studying the mechanisms that support a phenotype that is present in this particular cancer subtypes, which is the presence of an inverted polarity (polarity-out) that can be altered

upon interaction of tumor cells with the stroma. Although I can understand that the study is relevant, I have major concerns that should be taken into consideration:

1- Most of the study is done using 3 different PDX lines, with only one of them showing the phenotype of interest, which is the reversion of the polarity-out phenotype upon interaction with collagen/stroma. I would suggest having two more PDX lines displaying the same phenotype as PDX3 to be able to draw robust conclusions from the observations.

We thank the reviewer for raising this important point. We have collected additional samples from patient-derived CRC tumors, and isolated patient-derived organoids (PDO) that behave either like PDX1 and PDX2 (CRC0089; referred to in the revised MS as PDO#1), or like PDX3 (CGR0116, referred to in the revised MS as PDO#2) upon interaction with collagen. Importantly, in PDO#2, AIIB2 treatment prevents polarity reversal upon collagen inclusion supporting our previous findings in PDX3 that show CRC tumor cluster polarity is controlled by β1-integrin sensing the collagen matrix. These data are included in the revised manuscript in figures S2A-B and Fig2F-H, also below for your convenience. Furthermore, upon β1-integrin inhibition or HER2/HER3 inhibition PDO#2 fails to change polarity in collagen (similar to PDX#3) and upon HRG stimulation, PDO#1 adopts an apical-in polarity in collagen (like HRG-stimulated PDX#2). These data are included in the revised manuscript in figures S5G-H and below for your convenience. We feel that these additional clinically-relevant models provide important further validation of our data.

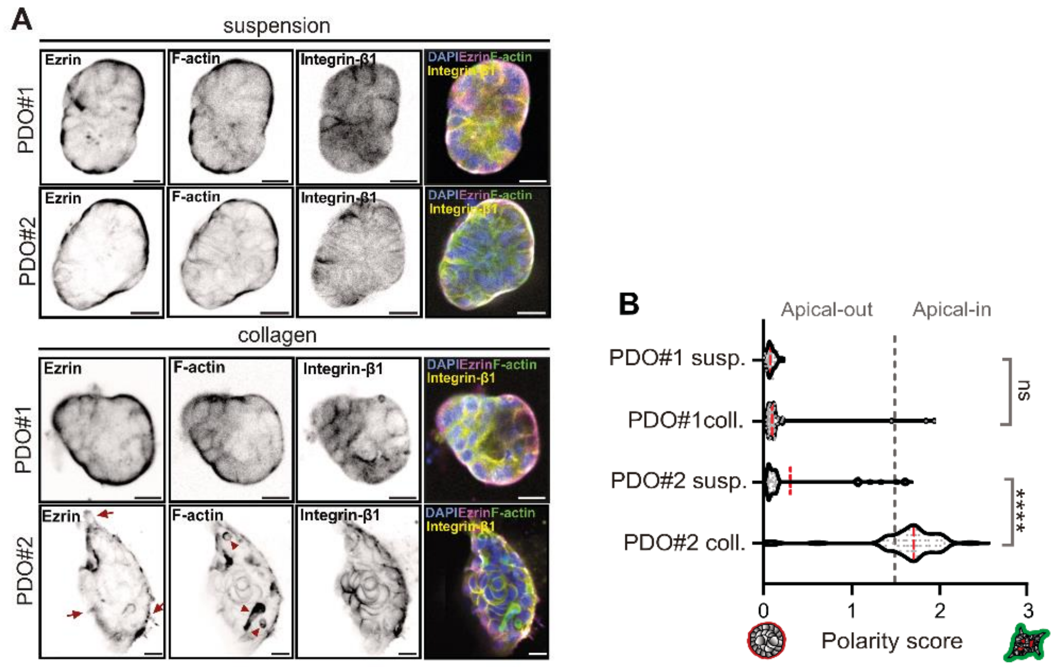

Manuscript FIGS2

(A) Representative immunofluorescence images of PDO#1- and PDO#2-generated spheres in suspension and in collagen, showing Ezrin, F-actin, β1-integrin and DAPI. In the PDO#2 collagen condition, arrows indicate protrusions and arrow heads indicate lumens. Scale bars 20 µm. (B) Polarity score of PDO#1- and PDO#2-generated spheres before and after inclusion in collagen for 3 days. Data are displayed as violin plots with mean indicated by a red line. Dashed line (at polarity score 1.5) indicates the mid-point between apical-in and apical-out characteristics. Mann-Whitney test for PDO#1: p value=0.107; n=55 (suspension); 59 (collagen) from four biological replicates, and for PDO#2: p value<0.0001; n=51 (suspension); 53 (collagen) from three biological replicates.

F

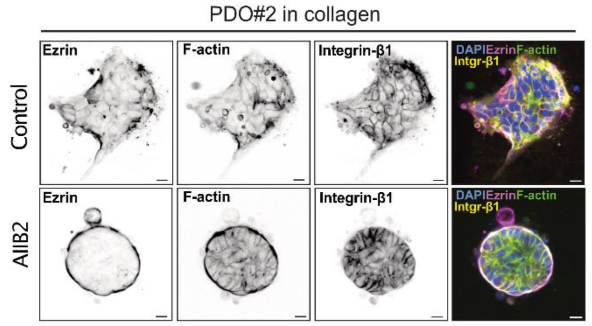

G

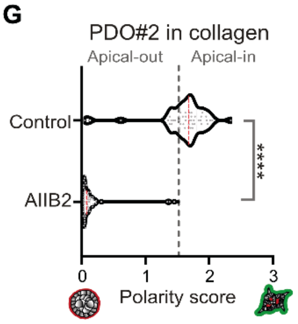

Manuscript FIG2

(F) Representative immunofluorescence images of PDO#2-generated spheres treated with AIIB2 and stained for the apical marker Ezrin, F-actin and β1-integrin. Scale bar 20 µm. (G) Polarity score of PDO#2-generated spheres in collagen ± AIIB2. Data are displayed as violin plots with mean indicated by a red line. Dashed line (at polarity score 1.5) indicates the mid-point between apical-in and apical-out characteristics [(n=45 (control); 50 (AIIB2) from two biological replicates, Mann-Whitney test; p-value=0.0001)].

G

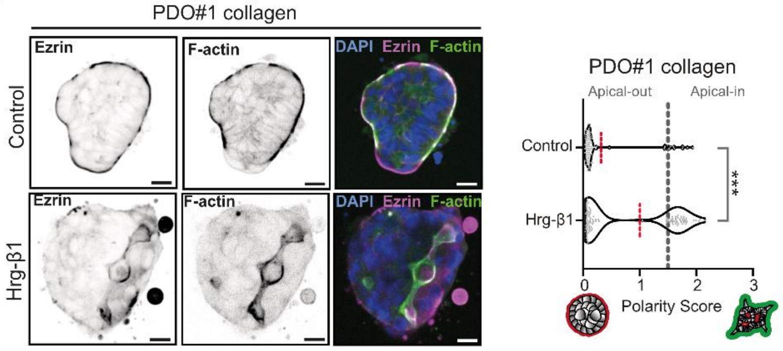

H

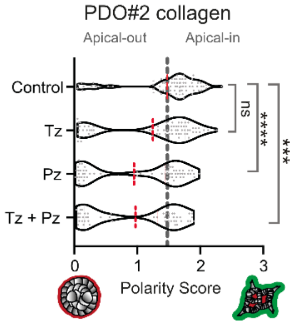

Manuscript FIGS5

(G) Immunofluorescence (Ezrin staining) and polarity score of PDO#1-generated spheres in collagen ± Hrg-β1 treatment (20 ng/ml). Scale bar 20 µm. For polarity score data are displayed as violin plots with mean indicated by a red line. Dashed line (at polarity score 1.5) indicates the mid-point between apical-in and apical-out characteristics (n_{Ctrl}=85 and $n_{Hrg\text{-}\beta1}$=121 from four biological replicates, Mann Whitney test, p-value=0.0003. (H) Comparison of polarity scores of PDO#2-generated spheres in collagen treated with HER2-blocking antibody (Tz), HER3-blocking antibody (Pz) or combination of both (Tz+Pz). Data are displayed as violin plots with mean indicated by a red line. Dashed line (at polarity score 1.5) indicates the mid-point between apical-in and apical-out

characteristics (Kruskal-Willis test with Dunn's multiple comparison tests: $p_{\text{ctrl vs Tz}}$=0,098 (ns), $p_{\text{ctrl vs Pz}}$<0,0001, $p_{\text{ctrl vs Tz+Pz}}$=0,0001; n(control)=67, n(Tz)=63, n(Pz)=69, n(Tz+Pz)=49 from three biological replicates).

2- In addition, I cannot agree with the conclusion that PDX3 flips from apical-out to apical-in when in contact with collagen. From the results shown, the apical-out polarity is clearly maintained in all conditions and in all PDXs, with the only difference being that small lumens seem to appear in PDX3 when adhered to the plate/collagen matrix (although only one spheroid is shown), and in the human tumors shown in Figures 7 and S7.

Thank you for this valuable comment. We fully agree that these are not typical apical-in organoids. Depending on the size of the patient-derived CRC organoids, we often found organoids with multiple layers of cells. As such, their polarity differs from the typical example of one-cell layered MDCK cysts with a single lumen in the middle. Nevertheless, we definitely observe a spectrum of different polarity phenotypes which we have scored using a single "polarity index" per organoid. Please note that even though only individual structures are shown, the polarity index provides unbiased quantification of multiple spheroids from several independent biological replicates and these are calculated in each experiment. The polarity index is a single measure but it reflects the number of protrusions (indicative of basally-localised integrin engagement with collagen), presence of lumens inside organoids (indicative of an apical-in organisation) and the ratio of the intensity of an apical marker (ezrin) on the membranes facing the lumen compared to the outer membranes. To clarify this, we have now defined apical-in polarity in text (page 4) as follows "whereas PDX#3 tumoroids in collagen revert their polarity into a multilayered structure with features of apical-in polarity, collagen-facing protrusion and Ezrin-positive lumens (Fig. 1A,B). While these multi-layered structures are not typical one-cell layered apical-in spheroids, they will be referred to as apical-in from hereon"

In my opinion, the main difference between PDX1-2 and PDX3 is the ability to adhere to the plate in the collagen cultures which should be further explored. Therefore, I don't think the concept of apical-out to apical-in shouldn't be used.

We apologise if the methodology was not clearly explained. The PDX tumoroids are not plated on collagen in plates but are rather embedded in a 3D collagen gel after being in suspension for 3 days. In these conditions, PDX#3 and PDO#2 extend protrusions, engage with the matrix, generate different sized lumens and partially display apical-in polarity.

3- Although I can understand that the study is relevant, I'm not entirely convinced that culturing cells in collagen is sufficient to mimic the interaction between cancer cells and the stroma. Additional matrixes need to be added in the experiments.

To address this important concern, we have tested the relevance of our observation of HER2/HER3 signaling regulating PDX#3 apical-in polarity in the context of the cancer cell stroma interaction in vivo. We have treated PDX#3 xenograft-bearing mice with control antibody or the combination of trastuzumab and pertuzumab for 3 weeks. The tumors were resected and submitted for immunohistochemistry analysis. HER2/HER3 inhibition resulted in a significant loss of polarized apical-in structures (based on H&E, alcian blue and pan-CK IHC and ezrin immunofluorescence staining). In addition, the treatment induced marked cell death in the tumors (Fig. 5G-K, supplementary figure S6 and below).

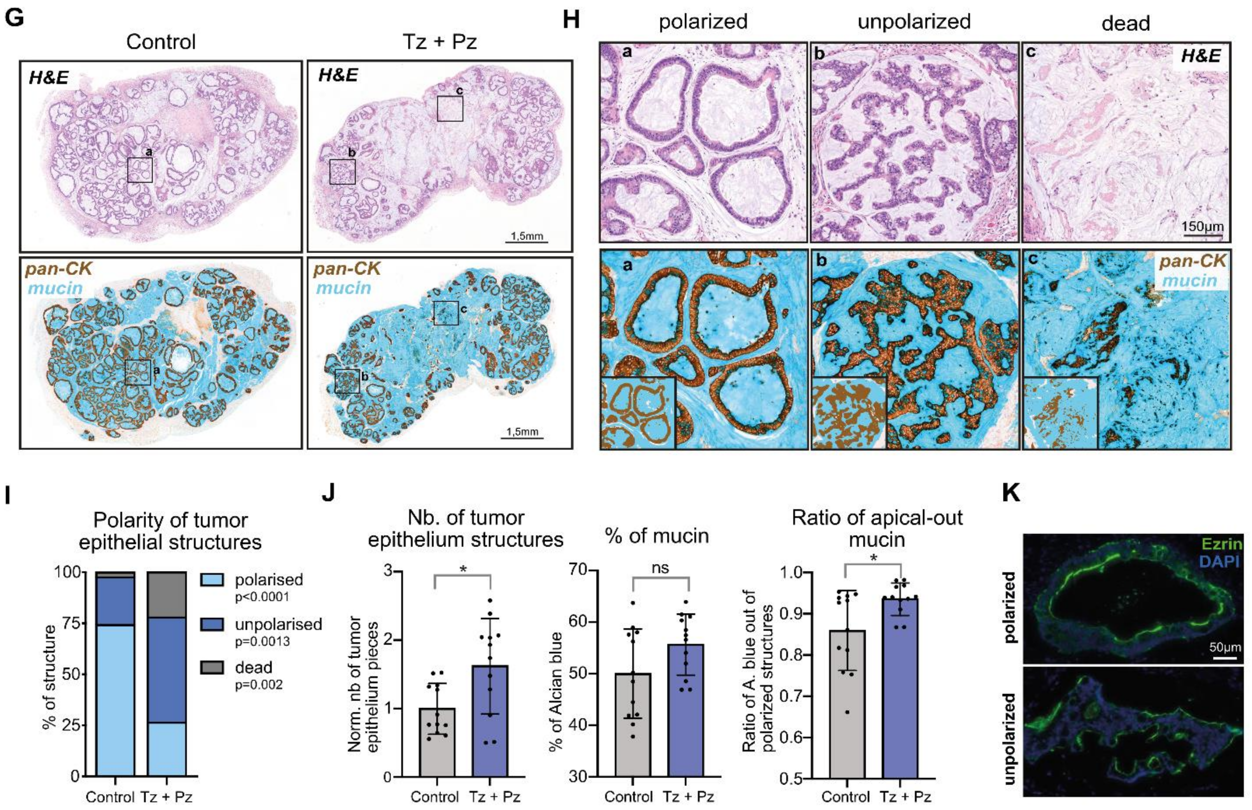

Manuscript FIG5

(G-H) Representative images (G) and zoomed-in insets (H) of Hematoxylin & Eosin (H&E), Alcian Blue and Pan-Cytokeratin (Pan-CK) stainings in PDX#3 tumors extracted from the flank of control vs treated (Tz+Pz, 10 mg/kg) mice. Scale bar, 1.5mm. Insets illustrate what was characterized as polarized, unpolarized and dead tumor epithelial structures based on the stainings. Insets on the Alcian blue images show the segmentation results used in quantitative analysis (See FigS6B). Scale bar 150 µm. (I) Qualitative classification of polarized, unpolarized and dead tumor epithelial structures in control and treated tumors shown as a percentage of the whole tumor. Statistical analysis was performed between the conditions for each indicated feature, p values shown in the graph (n=12, Welch's t test). (J) Segmentation-based quantification of the number of epithelial structures (Pan-CK) (p value=0.0139), percentage of mucin (Alcian blue) (p value=0.0771) and the ratio of apical-out mucin (p value=0.022) (mucin outside the closed tumor epithelial structures) comparing control and treated tumors (n=12). Mean +/- SD are shown as bar graphs with single data points displayed as black circles. Unpaired t-test with Welch's correction statistical test. (K) Representative images of polarized and unpolarized tumor epithelial structures based on ezrin immunofluorescence staining. Scale bar, 50µm.

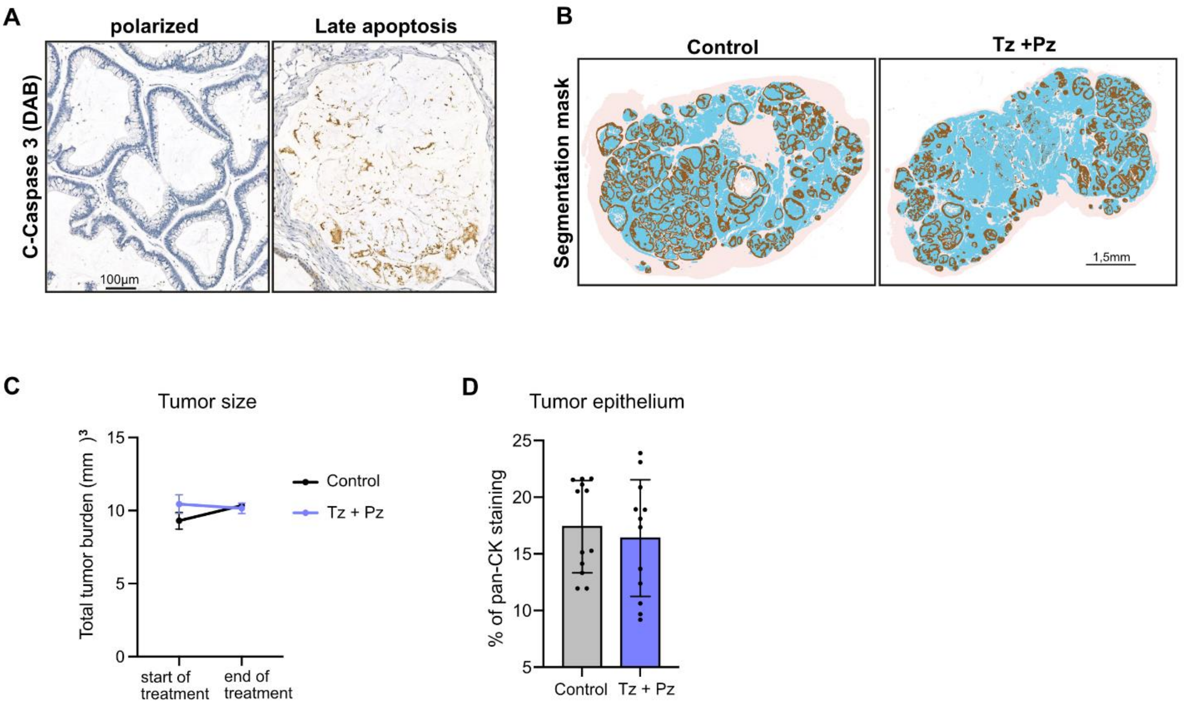

Manuscript FIGS6

(A) Representative images of tumor epithelial structures characterized as polarized (left) and dead (right), stained with cleaved caspase-3 antibody to show late apoptotic traces in the "dead" class. (B) Segmentation mask obtained from the representative images shown in Fig.2H. (C) Total tumor burden in control and trastuzumab and pertuzumab combination (Tz + Pz) treated mice. Average of total tumor size from both flanks is plotted (n=2 mice /group). (D) Quantification of the percentage of tumor epithelial structures (Pan-Cytokeratin mask) over the total size of the sample cut, comparing control and treated tumors (n=12). Mean +/- SD are shown as bar graphs with single data points displayed as black circles. Mann-Whitney statistical test, non-significant.

In addition, the collagen cultures contain 10% FBS, which in our hands is sufficient to promote organoid adhesion and spreading on plastic plates in the absence of collagen. Have the authors tested whether PDX3 needs collagen to acquire the adhesive/apical-in phenotype? Moreover, it is not clear from the methods what the non-adhesive medium contains. Is it the same medium as the one used to grow cells in collagen?

This is an interesting point even though we have not used 2D plating but rather 3D gels in our experiments. We tested whether 10% FBS alone was sufficient to promote adhesion and spreading on plastic in the absence of collagen. We detect adhesion and spreading on collagen-coated but not on uncoated plates (see figure 1 for reviewer #4). The composition of the suspension condition has been clarified in the text (p28); "culture medium (DMEM supplemented with 10% FBS)."

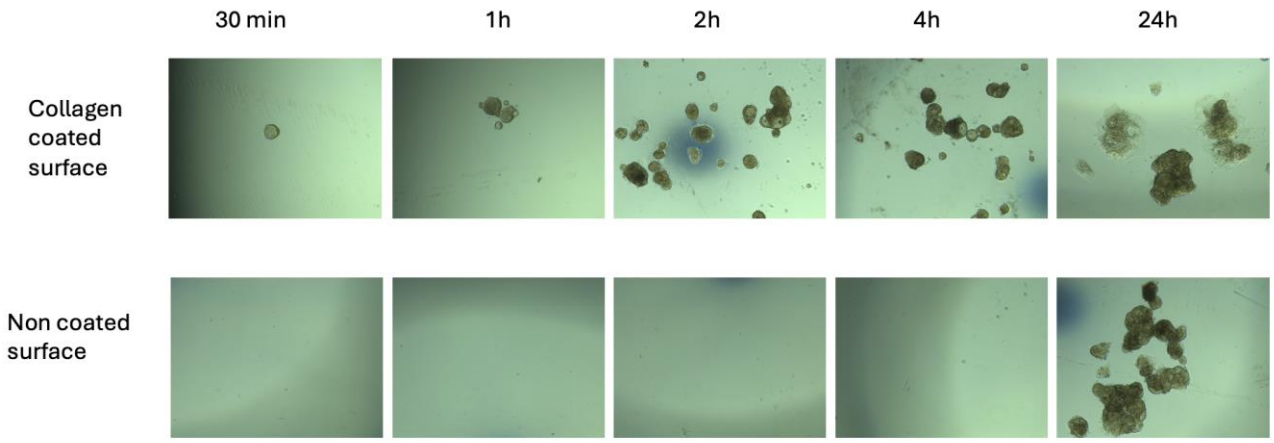

FIG1 for Reviewer#4.

PDX#3-generated organoids spreading over time on collagen-coated (10 ug/ml) and uncoated surfaces.

4- Authors have performed whole genome sequencing of the three PDXs. Since TGFb is involved not only in the polarity responses to the stromal environment, as the authors explain, but also in migration and adhesion processes, is any gene involved in the TGFb pathway mutated in the PDXs that could explain the differences in behavior on collagen culture?

Thank you for this interesting question. In our previous publication, we reported the following: "Exome sequencing of TGFβ receptors and effectors identified that all three PDX models harbored the TGFBR2 K153fs frame-shift mutation reported to decrease TGFβ signaling (de Miranda et al., 2015), whereas only PDX#2 displayed a Smad4 W268* mutation, truncating half of the protein (Fig. 4C). This likely explains why this tumor is insensitive to TGFβ stimulation' (PMID: 35703098)." The study shows that TGFβ stimulation induces an increasingly apical-in polarity in PDX#1 in collagen but interestingly, not in PDX#2, which in this study is shown to respond to HRG stimulation (unlike the unresponsive PDX#1). The study also demonstrates that PDX#3 polarity is sensitive to TGFβ inhibition even though the cells harbour the mutation in TGFBR2 (K153 frame shift mutation) that decreases its signaling.

Therefore, while TGFβ signaling plays a role in these cells, it is insufficient to explain the biology. Here we uncover three novel players in CRC organoid polarity determination in collagen: SorLA, HER2 and HER3.

5- Regarding the secretion of mucins, only one (not very conclusive) image of a lectin staining is shown in S1B without any quantification. Additional IF/IHC for specific mucins and some quantification could support authors' conclusions. I have also noticed that the mucin staining is no longer shown in the experimental conditions tested in the other figures, which would be informative and supportive.
This is a valid question. These PDXs are from tumors that were classified as mucinous based on their high mucin content. In addition, we now show with new in vivo data that PDX#3 has a very high mucin content (alcian blue staining of the tissue). While we agree that investigating the exact mucin content of these models would be interesting, we feel that it falls beyond the scope of this study. However, we have acknowledged this in the text (page 4). "However, we have not analysed which specific mucins are secreted and if this impacts the phenotype"

6- In Figure 3, authors perform RNA-seq to identify putative regulators of integrin b1 trafficking. However, western blot levels of RCP poorly represent what is obtained by RNA-seq (RCP expression is much higher in PDX1 than in the others) and the western blot shown does not match with the values in the quantification.

We agree that mRNA and protein levels do not match fully. However, this is the case with many proteins, where mRNA levels do not fully reflect protein levels due to complex posttranscriptional regulation including protein stability and degradation rates. Furthermore, we would like to point out that the loading is different in the example WB and since the quantifications are normalized to the loading, the RCP signal in the representative blot and the quantifications look different.

Moreover, the KD of SorLA is minimal and the WB is of very low quality.

We agree that the KD efficiency is not optimal. However, these shRNA experiments in primary PDX tumoroids isolated from the mouse are extremely challenging and in spite of our efforts we have unfortunately not been able to improve the WB quality. Quantification shows 50% reduction of SorLA levels and the polarity scores also highlight two distinct populations where the pool of tumoroids remaining apical-in are likely to represent the non-silenced subpopulation.

The same problem is found in the WB analyses in 5A-B, where the GAPDH control is absolutely variable between samples, making the quantification presented scarcely credible. In general, some of the conclusions drawn are not entirely correct, which does not invalidate the results obtained, but makes it difficult to follow the story (a constant comparison of what is said in the text and what is shown in the figures is necessary).
This is a valid point. These are technically challenging experiments. Material from the PDX tumors is limited. Furthermore, isolation of the PDX tumoroids from collagen results in different degrees of collagen carryover, which complicates normalisation based on protein content resulting in uneven loading. As we have been unable to perfect this, albeit attempting multiple times to reload and re-run samples to obtain more even loading, we have moved these data to the supplement and edited the results section to tone down the emphasis on the significance of the data. "Although HER2 is significantly upregulated in both PDX#2 and PDX#3 in collagen, HER3 induction was more variable in PDX#2 and reached significance only in the collagen-responsive PDX#3 (Fig. 5A-B).

We have also carefully edited the manuscript text to make sure the conclusions are in line with the results obtained.

7- Then p-ERK appears in the scenario as a downstream effector of integrin/ECM engagement, but only p-ERK levels from PDX3 are shown. The authors should show p-ERK in the other PDXs to be able to conclude that this is something specific to the PDX3 phenotype and not something unrelated.

Thank you for this interesting suggestion. We have embedded PDX#1 and PDX#2 in collagen and analysed their pERK levels similarly to what was shown with PDX#3 in Fig 3H and S3C. We find that pERK levels are not induced in these PDXs in collagen (Figure 2 for reviewer#4 below).

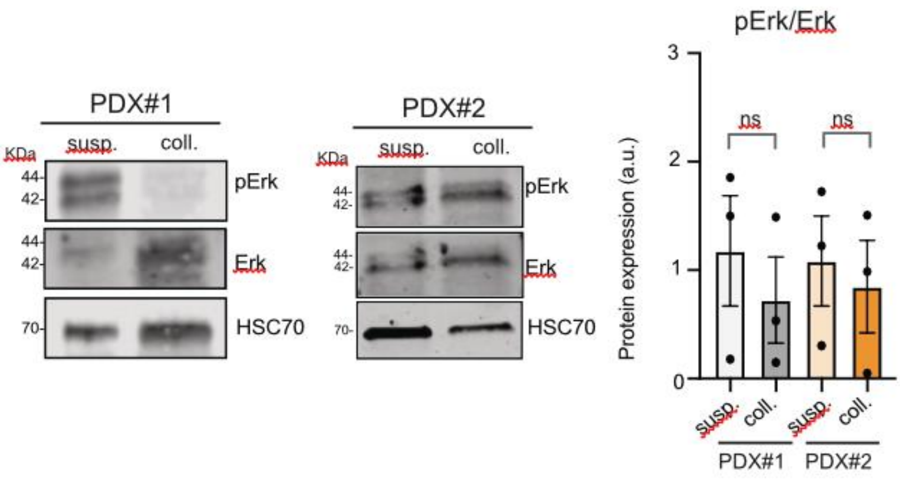

FIG2 for Reviewer#4.
Western blot of Erk, pErk and HSC70 (loading control) and corresponding quantifications of Erk phosphorylation in PDX#1- and PDX#2-generated tumoroids in suspension and in collagen (n=3 biological replicates, unpaired t-test, non significant).

8- HER2-3 could be better integrated in the final model since the authors have previously shown the role of SorLA in HER2 regulation in breast cancer.
Thank you for this point. We have indicated now clearly in the model in Fig 7F HER2/HER3 signaling and the crosstalk with SorLA.

In general, although I find the study interesting, the data presented do not support the authors' conclusions.
We hope that all the additional experimentations and models used, and our responses to the other reviewers now convince this reviewer that our data support the conclusions drawn.

Reviewer #5 (Remarks to the Author):

I co-reviewed this manuscript with one of the reviewers who provided the listed reports. This is part of the Nature Communications initiative to facilitate training in peer review and to provide appropriate recognition for Early Career Researchers who co-review manuscripts.

Point-by-point response to reviewer comments

Reviewer #1 (Remarks to the Author):

The revision has addressed several points raised in previous review. It is understandable that some concerns, such as Point #7 is technically challenging to address. However, two key points #4 and #6 remain unanswered and requires additional data support. These two points are critical to support the main model that collagen-induced α2β1-integrin engagement upregulates SorLA, which drives rapid β1-integrin recycling to trigger polarity reversion. Current data show that SorLA is involved in collagen-induced polarity reversion, but it is somewhat uncertain whether this is due to the role of SorLA in integrin recycling. Answering even one of these two points could provide some much-needed support.
We are grateful to the reviewer for their constructive comments and appreciation of the technical challenges of mechanistic cell biology experiments using PDXs and PDOs in a 3D matrix setting. We felt that both of the points raised are important and focused our efforts on addressing both of them with new experiments and with textual revisions.

Previous point #4: Fig. 1C and Video 1 shows that clear formation of lumen-like structures and protrusions could be observed as early as 5 hours post-embedding. It would be important to determine how quickly upregulation of SorLA and maturation of integrin β1 occurs to contribute to the phenotype.
This is indeed an interesting point. Our data show that only PDXs and PDOs with initially high integrin levels (PDX#3 vs the others; PDO#2 vs PDO#1) are able to engage the matrix and generate protrusions. The cells secrete high amounts of mucin that very quickly forms a physical barrier surrounding the structures, blocking their access to the surrounding collagen. Based on this, we conclude that immediately after embedding there is a short window of opportunity for cells to access the ECM and only the high-integrin PDXs and PDOs are able to generate the kind of adhesive and force transmitting protrusions seen for example in Video 1 , which are absent in the integrin-blocking antibody condition in Video 2. Therefore, the initial response to the matrix is most likely mediated by the already high integrin, and, then, reinforced by SorLA upregulation and integrin maturation in long-term culture.

As shown in Fig. 3E, even though PDX#3 has high integrin α2β1, the expression of SorLA is dependent on collagen binding as PDX#3 in suspension shows low SorLA expression similar to in PDX #1 and PDX#2. Could integrin α2β1 and SorLA levels be monitored by IF following collagen embedding to support this claim? To conclude that SorLA upregulation by integrin α2β1 activation is required for polarity reversion, it is critical to determine the spatiotemporal pattern of integrin α2β1 and SorLA protein post-embedding to promote polarity reversion. This is especially critical since the current data do not directly show that SorLA regulates β1-integrin recycling in PDX#3, therefore missing the link between SorLA in β1-integrin recycling and SorLA in polarity reversion.

We thank the reviewer for these important comments. To further investigate the spatiotemporal relationship of SorLA and α2-integrin during collagen-induced polarity reversion, we performed a time-course immunofluorescence analysis (0-72h) following collagen embedding using PDX#3-generated organoids (PDXO#3).

Consistent with our previous observations, SorLA expression was low in PDXO#3 in suspension (0h collagen) and remained weak during the early stages of collagen embedding. Over time, however, the SorLA signal increased progressively. Quantification showed a 2.5-fold increase (± 0.73 s.e.m) in SorLA signal 16h post collagen embedding, while at 24h and 72h the signal increased by 5-fold (± 0.71 s.e.m) and 5.8-fold (± 1.0 s.e.m) respectively, compared with spheroids in suspension. We have included this in the new Fig S4F (and below) and updated the text on page 13: "*We observed increasing SorLA levels upon collagen-embedding during 72 hours (Fig. S4F)*". At the later time point, clear accumulation of SorLA was evident particularly in the protrusive regions of the collagen-embedded spheroids. Taken together, these observations further support the upregulation of SorLA following collagen embedding in PDX#3-generated organoids.

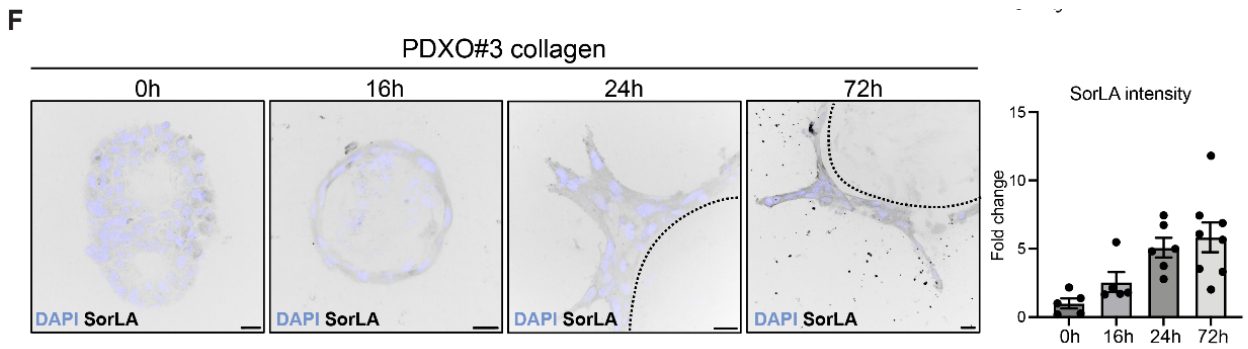

Manuscript FIGS4.

(F) Representative immunofluorescence images of PDX#3-generated organoids (PDXO#3) fixed in suspension (0 h in collagen) or embedded in collagen (16-72 h) and stained for SorLA and DAPI. Lumens in 24 h and 72 h PDXO#3 are indicated with a dashed line. Scale bars 20 µm. All images were acquired using identical microscope setting, and the same brightness and contrast parameters were applied to allow visualization of the relative fluorescence signals across the collagen-embedding time course in comparison to the average of 0 h.

Previous Point 6. Page 6, "These data suggest the possibility that the PDX#3 tumoroids in collagen switch from RCPmediated long-loop integrin recycling to a SorLA-driven rapid recycling pathway in a manner that is dependent on initial matrix-induced integrin signaling." requires additional experimental support. The authors should examine the colocalization of RCP or SorLA with integrin proteins in suspension vs. collagen-embedded organoids to support this conclusion.
We agree with the reviewer that this conclusion was not fully supported by the data. We have unfortunately been unable to obtain high-quality immunofluorescence staining of RCP in the collagen-embedded tumoroids. In response to the criticism, we have edited the text to: "*These*

data suggest the possibility that the polarity switching of PDX#3 tumoroids in collagen is supported by ERK-induced SorLA expression" (page 10).

Since the role of SorLA in integrin recycling was only demonstrated in CRC cell line LS513, but not in PDX#3, the conclusion "Apical-in polarity in collagen is established through SorLA-dependent β1-integrin recycling" requires such experimental support.
To further explore the relationship between α2-integrin and SorLA, we performed a time-course immunofluorescence analysis (0-72h) after embedding PDXO#3 spheres in collagen (new Fig. S4G and below). Consistent with our previous observations, and the data provided above, SorLA expression was low in PDX#3-generated organoids in suspension (0h collagen). Following collagen embedding a colocalization of SorLA and α2-integrin was detected at 16h time point. This overlap became more evident at later time points, at 24 and 72h. Based on these findings the text was edited on page 13: "*We observed increasing SorLA levels upon collagen-embedding during 72 hours (Fig. S4F) and detected overlapping vesicular puncta of α2-integrin and SorLA (Fig. S4G)*". Together, these observations provide additional spatiotemporal evidence linking SorLA and α2-integrin following collagen embedding, further supporting the connection between integrin signaling, SorLA and polarity regulation in PDX#3-generated spheres.

G

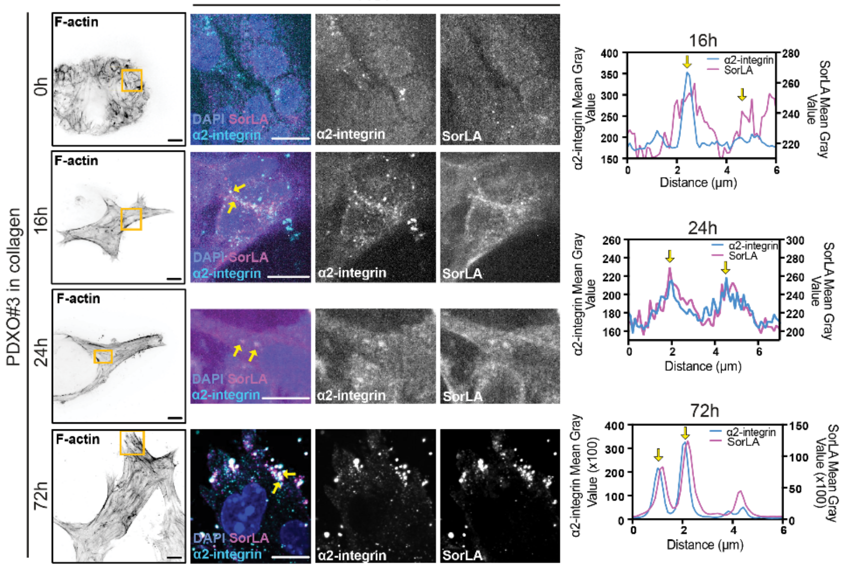

Manuscript FIGS4.

(G) Left: Representative immunofluorescence images of PDXO#3 fixed in suspension (0 h in collagen) or embedded in collagen over time (16-72h) and stained for SorLA, α2-integrin, F-actin and DAPI. F-actin maximum intensity projections show sphere morphology and protrusions. Yellow boxes indicate the zoomed-in insets (ROI) and yellow arrows highlight overlapping SorLA and α2-integrin signals plotted in the graphs on the right. Scale bar 20 μm (main),10 μm (insets). All images were acquired using identical microscope settings. Since signal intensities varied between early and late time points, brightness and contrast were adjusted individually for each image to visualize SorLA and α2-integrin staining patterns.

Reviewer #2 (Remarks to the Author):

The authors responded appropriately to my comments. Following are my additional comments. I commend the authors' efforts to add other organoids and present organoids exhibiting a similar response to PGX#3. However, the number remains small. The authors should confine their assertion to stating that ‘this mechanism exists in some MUC CRC patients.
We thank the reviewer for their positive and supportive comment. We fully agree with the point raised about the small numbers. To acknowledge this, we have included the following statement in the text on page 8.

“Taken together, these data indicate that α2β1-integrin-mediated cell interaction with collagen-I, and the ensuing downstream signaling, are required for the phenotypic polarity reversion in at least some MUC CRCs. However, given the limited number of models examined, these findings cannot yet be generalized to all MUC CRC patients.”

The differences of the polarity score are marginal in some of the experiments. In such cases, other pathways are also likely to be involved. The authors should state that the molecular mechanisms underpinning polarity reversal may be more complex and diverse.
I commend the authors' efforts in adding trastuzumab and pertuzumab treatment in vivo. Indeed, changes in polarity and cell death are observed. Although the pattern of change is localized, I consider it worthy of future investigation to understand why such a patterned change occurs.
I still cannot agree to the use of the collagen infiltration assay as a model for peritoneal seeding. I fully concur that exposure of the peritoneal basement membrane promotes dissemination. It would be preferable to present the basement membrane infiltration assay as it is, without using it as a peritoneal seeding model, and to discuss it solely as a factor promoting peritoneal seeding within the discussion section.
We fully agree and have now included the following in the beginning of the discussion on page 22:

“While, our data show that perturbation of these pathways inhibits collagen-induced polarity reversal, the molecular mechanisms underpinning polarity reversal may be more complex and diverse.”

We have also revised the text to present the basement membrane infiltration assay as suggested. We removed this statement from the end of the results section “*and spreading onto the peritoneum*” in addition we have edited this section in the results to remove any claims about in vivo seeding of the peritoneum.

Earlier version:

“To study CRC peritoneal invasion with high granularity and in response to various treatments, we set up an in vitro model that recapitulates advanced CRC invasion of the peritoneum. In the peritoneal cavity, free floating cancer cell clusters usually adhere onto the mesothelial cell layer or, more often, directly to the underlying ECM, as the peritoneal cell lining is usually damaged

during laparoscopic surgery and early cancer treatment (Cortés-Guiral et al, 2021). To mimic this late stage of CRC invasive progression, we isolated and de-cellularized mouse peritoneum, a technique previously applied to the mouse mesentery (Glentis et al., 2017), which was shown to recapitulate the organization and characteristics of the in vivo basement membrane (Varinelli et al., 2022)."

Edited version (page 18):

"To study CRC interaction with the peritoneal basement membrane with high granularity and in response to various treatments, we set up an in vitro model. We isolated and de-cellularized mouse peritoneum, a technique previously applied to the mouse mesentery (Glentis et al., 2017), which was shown to recapitulate the organization and characteristics of the in vivo basement membrane (Varinelli et al., 2022). We plated the PDX tumoroids on the de-cellularized peritoneum (Fig. 6F)"

Reviewer #3 (Remarks to the Author):

I thank the authors for addressing my comments. I would just recommend that the authors interpret the data from the Human Protein Atlas with caution. Not all clones that have been tested are performing well. For instance, the HER3 IHC that was included in the reply to the Reviewers clearly lacks specificity.

We thank the reviewer for this important comment and fully agree that the data from the Human protein Atlas must be interpreted with caution.

Reviewer #4 (Remarks to the Author):

In this revised version of the manuscript, the authors have included additional data to address some of the initial concerns raised by the reviewers. They have provided a more detailed definition of the apical-in phenotype and incorporated an additional model with characteristics similar to PDX3. However, in my view, several critical issues still require adequate clarification and resolution.
PDX models and phenotype:
While it is understandable that increasing the number of PDX models may not always be feasible, particularly when dealing with rare or specific phenotypes, it remains unclear how many PDXs have been analyzed beyond those presented in the manuscript. It would be important to know what proportion of the total tumors evaluated display this specific phenotype, in order to assess its prevalence and biological relevance.
To begin to address this concern, we have included the following statement in the text on page 8:

"Taken together, these data indicate that α2β1-integrin-mediated cell interaction with collagen-I, and the ensuing downstream signaling, are required for the phenotypic polarity reversion in at least some MUC CRCs. However, given the limited number of models examined, these findings cannot yet be generalized to all MUC CRC patients."

Previous publication reported that PDOs from 2 out of 7 mucinous CRC samples display only apical-in phenotype (Canet-Jourdan et al, 2022). A screen of 6 additional novel muc-CRC samples revealed the presence of one apical-in only tumor (PDO#2). Taken together these two screens highlight that a non-negligible fraction of muc-CRC (23%) display the specific full apical-in phenotype reported for PDX#3. We chose to restrict our study to the analysis of these tumors in order to decipher the underpinning mechanism of ECM-based polarity reversion.

In addition, we noticed in these two screens, but also in another cohort including 36 patients (published in Canet-Jourdan et al, 2022, Fig 7A), that most tumors generate both apical-in and apical-out PDOs. We reported in that publication that 30 out of 36 mucinous CRC tumors (83%) presented with at least 25% of apical-in clusters, *in situ* (Canet-Jourdan et al, 2022, Fig 7B). The co-existence of both phenotypes could be explained either by genetic diversity within the tumor or due to spatial heterogeneity in the microenvironment.

This is mentioned in the discussion of the manuscript (page 22) as follows: "*In this study we focused our investigation on PDXs and PDOs with only apical-in or apical-out phenotypes in order to decipher the underpinning mechanism of ECM-based polarity reversion and the features distinguishing these distinct polarities. However, in a cohort including 36 MUC CRC patients (Canet-Jourdan et al, 2022), it was observed that most tumors show marked heterogeneity and generate both apical-in and apical-out PDOs. These may reflect either genetic diversity within the tumor, spatial heterogeneity in the microenvironment or plasticity of polarity at different stages of tumor development; this however remains to be investigated in the future.*"

Culture conditions:
Based on our experience, when cultured in DMEM supplemented with 10% FBS, even PDOs growing in 3D tend to sink and adhere to the plate, thereby resembling the phenotype shown by the authors and defined here as “apical-in”. Although our observations were made using Matrigel, it would be helpful to include lower-magnification images to provide a more comprehensive view of what is occurring in these cultures.
Thank you for this valuable comment. We agree that sphere adhesion to the plate can occur under certain culture conditions. However, we have optimized our culture system to ensure that tumoroids, PDXOs and PDOs remain in Matrigel or collagen-I and do not sink or attach to the plastic surface. Specifically, once organoids are generated and passaged in Matrigel, the culture plates are placed upside down during Matrigel polymerization to prevent the organoids from settling at the bottom. We have now included this information in the Methods section (page 27): “*During polymerization, the cell culture plate was placed upside down to prevent the PDXOs from settling at the bottom.*” Additionally, we have updated the description of the collagen-embedding protocol (page 28) to specify that wells were precoated with a layer of neutralized and polymerized collagen-I gel to prevent any direct contact between the spheres and the plastic surface.

It is important to note that all experiments throughout the study were performed in fully 3D conditions. As shown in FIG1 for Reviewer #4, orthogonal projection of immunofluorescence images confirmed that spheres were fully embedded within the collagen and thus were not able to adhere to the plate.

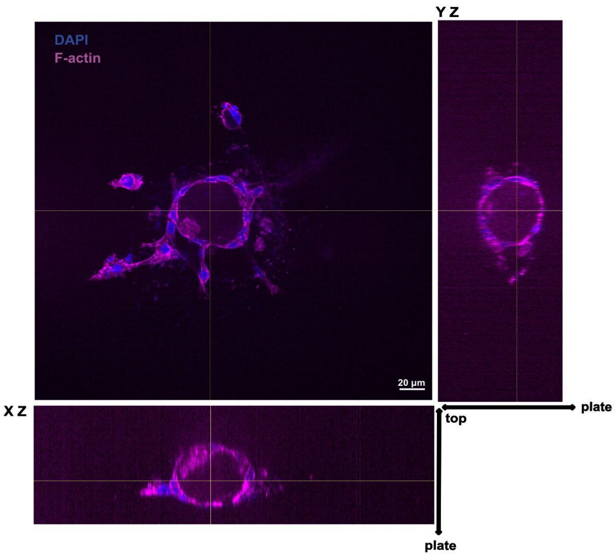

FIG1 for Reviewer #4.

Representative immunofluorescence image of PDX#3-generated sphere (PDXO#3) in collagen. Orthogonal projections (XY, XZ and YZ) are shown with arrows indicating the position of the sphere within the collagen.

This concern also applies to Figure 1, where no spheres appear to be present in the non-coated cultures. To my knowledge, differences should primarily be reflected in the number of adhered cells or spheres, rather than in the total number of cells present in the cultures.
Following the previous comment from Reviewer#4, we performed the requested adhesion experiment under 2D conditions using both collagen-coated and non-coated surfaces in the presence of 10% FBS in the culture medium. We detect adhesion and spreading on collagen-coated but not on uncoated plates at early time points (30 min-4h). These results were shown in Figure 1 (for Reviewer #4, previous rebuttal letter). These data demonstrated that the presence of FBS was not sufficient to promote organoid adhesion and spreading to the plastic surface. We sincerely apologize for not providing sufficient details about the experimental setup earlier in the rebuttal letter. Equal amount of PDX#3-generated organoids were plated on both collagen-coated (10 ug/ml) and non-coated wells (in DMEM supplemented with 10% FBS), having individual wells for each timepoint. Before imaging, wells were gently washed with PBS to remove non-attached spheres, and only the adhered (or partially adhered) spheres were imaged.

Alcian Blue staining: It is positive that the authors have included Alcian Blue staining to visualize mucins, although the original request was to assess specific mucins (that could be selected based on RNA-seq data). That said, I have serious concerns regarding the quality of the staining presented. The background signal is extremely high, making it difficult to clearly discern what is being quantified. We have performed similar staining in CRC tumors, and I find it difficult to understand why such a high background is present in these samples. Could the authors optimize the staining protocol to improve the quality and interpretability of the mucin signal?
Thank you for this concern. The Alcian Blue staining in these samples has been further confirmed by a pathologist (Dr. M. Bani) from Gustave Roussy, who verified that the staining is indeed specific. As shown in the FIG2 for Reviewer #4, magnified regions without mucin remain negative for Alcian Blue, supporting the specificity of the staining. Moreover, comparable mucin staining patterns can be observed in Canet-Jourdan et al. (2022) in Figure 7A. More importantly, it is relevant to highlight that the tumors generated by PDX#3 are highly mucinous.

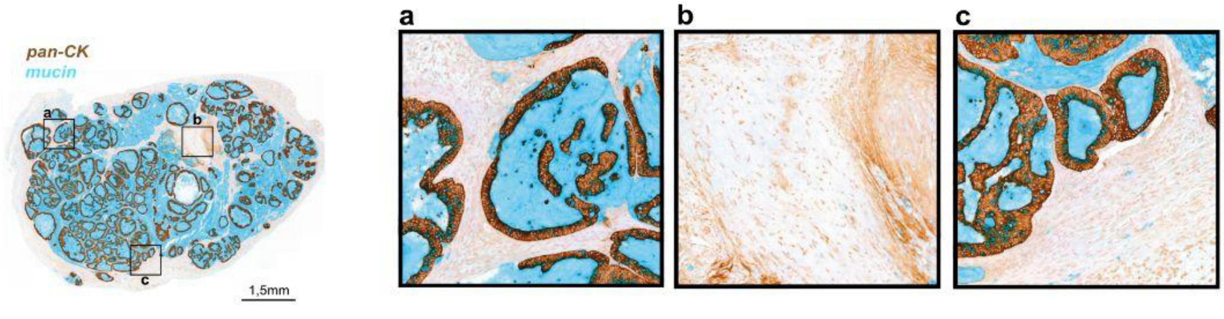

FIG2 for Reviewer #4.

Representative image and zoomed-in insets (a-c) of Alcian Blue and Pan-Cytokeratin (Pan-CK) stainings in PDX#3 tumors extracted from the flank of control mice. Magnified regions highlight the Alcian blue-positive mucinous and Alcian blue-negative, mucin-free, regions of the tumor.

Western blot analyses:
I continue to have concerns regarding the western blot data, particularly given that the authors have not attempted to improve the quality of the results shown (although they say they tried). While I appreciate that sample availability may be limited, the authors have had sufficient time to generate new material and lysates to provide more robust and reliable data to support their conclusions. In addition, the manner in which several blot panels have been cropped is problematic, as it hinders assessment of data consistency across replicates. Overall, the quality of the western blot data does not appear to meet the standards expected for publication in Nature Communications.
p-ERK results: Regarding the p-ERK data, it is plausible that p-ERK is induced in PDX3, as suggested by the western blot shown in the initial submission. However, given the high variability observed between replicates, now clearly evident from the PDX1 and PDX2 data, it is reasonable to question whether p-ERK levels in PDX3 are truly less variable. To support this conclusion, equivalent biological replicate data for PDX3 should be presented.
To alleviate the reviewer's concern and in line with journal policy, we have now provided the original uncropped images of all western blots in the manuscript. It also came to our attention that the image quality was negatively impacted by the export settings we had used and for which we apologize. In addition, the data were mean +/- SD. In the current figure, we are now displaying the s.e.m similar to the data we showed for PDX#1 and PDX#2 in the previous rebuttal letter and below for your convenience.

In respect to the specific concern regarding the ERK phosphorylation data and conclusions for PDX#3, we have included all the western blot repeats (n = 4) for the reviewer below (FIG3 for Reviewer #4). Retrieval of cells from the collagen is a continued challenge often resulting in lower loading in these samples (they are normalized for protein content but the carryover from the collagen gels complicates this). However, as shown below, we consistently see in each PDX#3 experiment increased pERK/ERK ratio in collagen. In contrast, the experiments with PDX#1 and #2 show no consistent trend or response to collagen (for more details please see notes in the figure). We believe that these data are clear and in line with our conclusions. As appreciated by the reviewer, sample generation is a limiting factor and we had chosen to focus on performing the new experiments requested in the revision process, while respecting the 3R principle. We hope that the data below convince the reviewer that our observations are indeed robust.

ERK phosphorylation in PDX#3 in suspension versus collagen

Graph in main Figure 3H and representative blots in Supplementary Figure 3C

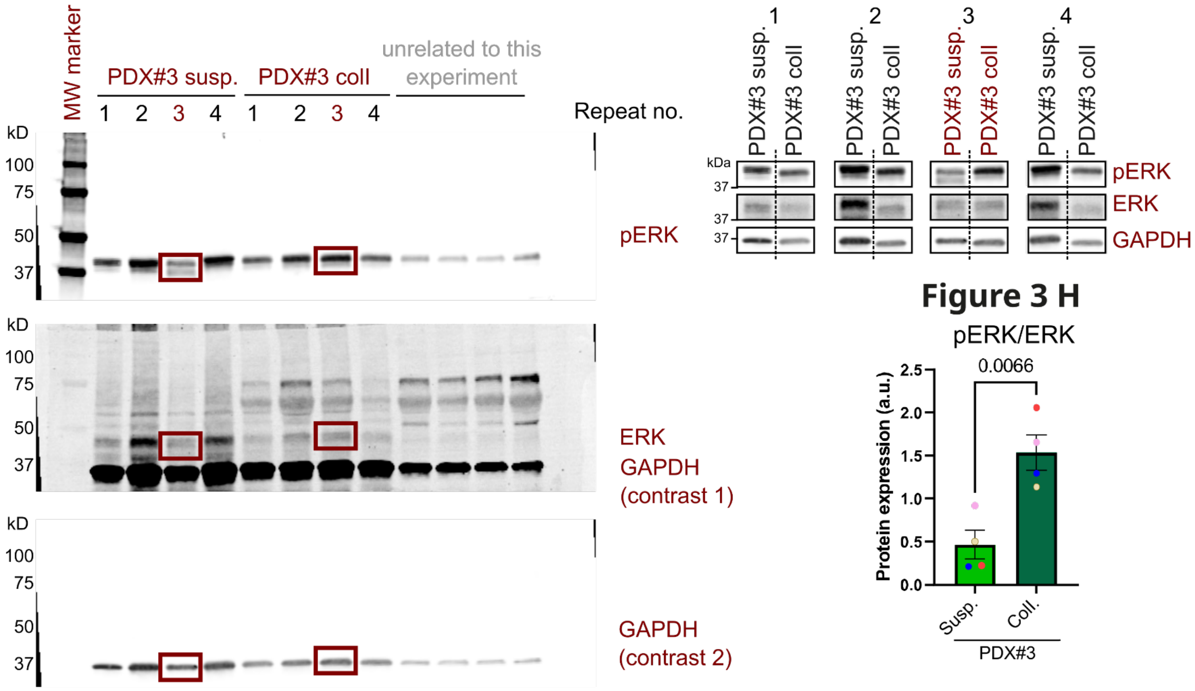

PDX#3 data: Low total ERK in repeats 1, 2 and 4 in the collagen condition due to loading (reflected in GAPDH loading). Despite this, pERK band is robustly detected in all collagen condition repeats.

Repeat 3 was chosen for display purposes in Supplementary Figure 3C as the loading was more equivalent between the conditions.

ERK and pERK were normalised to loading control to take into account the difference in protein loading.

The ratio of pERK to ERK was then calculated (Figure 3H; data are mean +/- s.e.m; Each biological repeat is represented by a different colour). Note, in all repeats, pERK levels are increased in the collagen versus suspension condition.

ERK phosphorylation in PDX#1 and #2 in suspension versus collagen

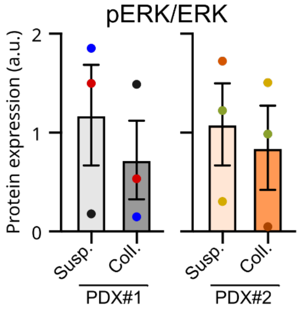

PDX#1 and #2 data included in previous rebuttal: As noted by the reviewer, pERK/ERK ratios are quite variable in the experiments with PDX#1 and PDX#2. There is no discernable pattern when comparing suspension versus collagen (each repeat is represented by a different coloured symbol). Given the robust results with PDX#3, this suggests no effect on pERK levels (data are mean +/- s.e.m) and repeating this experiment is unlikely to change the result.

FIG3 for Reviewer #4.

Western blot data for ERK phosphorylation.

Reviewer #5 (Remarks to the Author):

I co-reviewed this manuscript with one of the reviewers who provided the listed reports. This is part of the Nature Communications initiative to facilitate training in peer review and to provide appropriate recognition for Early Career Researchers who co-review manuscripts.

Thank you for your feedback to improve this study.
